# Supplementary material for: Gestational Hypoxia Disrupts Medial Ganglionic Eminence Progenitor Dynamics and Interneuron Development in Schizophrenia
Source: Adv Sci (Weinh). 2026 Aug 12:e76936. Online ahead of print. doi: 10.1002/advs.76936 (PMC13464456; doi:10.1002/advs.76936)
Supplement: Supplementary file 1 [file ADVS-9999-e76936-s002.docx]

**Figure S1. Characterization and genomic validation of iPSC lines**

***Related to Figure 1***

(A) Normal karyotypes of all iPSCs at passage 20. (B) Bright-field and immunofluorescence images show typical iPSC morphology and expression of OCT4 and TRA-1-60. Teratomas, stained with hematoxylin and eosin, reveal differentiated tissues representative of all three germ layers: endoderm (endo, gut epithelium), mesoderm (meso, cartilage), and ectoderm (ecto, neural tissue) White scale bars = 100 μm, black scale bar = 200 μm. (C-D) Representative flow cytometry plots showing expression of pluripotency surface markers SSEA4 (C) and TRA-1-81 (D) across different iPSC lines. Each color represents a distinct iPSC line, indicating consistent expression of both markers among lines. (E-F) Bar plot showing the relative mRNA levels of *OCT4* (E) and *NANOG* (F) in HC and FES iPSC lines. Expression was normalized to GAPDH and is presented as mean ± SEM. ** *p* < 0.01, *** *p* < 0.001，**** *p* < 0.0001. H1, positive control; hMSCs, negative control. H1: n = 3 batches; HC: n = 16 batches; FES: n = 12 batches; hMSCs: n = 3 batches. (G) Analysis of residual exogenous gene copies using qPCR, color-coded by cell lines. The value below 0.1 indicates the absence of exogenous gene integration. (H) PCA of PBMCs (circles) and matched iPSCs (triangles) from all subjects based on the WGS data (color-coded by cell lines). (I) Upset plot presentation of the matrix layout of mutant genes in PBMCs. The lateral color columns presented the number of mutant genes in each line (color-coded by cell lines). The black vertical columns showed the number of mutant genes specific in each sample (single dot without vertical lines) or the number of shared mutant genes cross samples (dots stringed by black lines).

**Figure S2. Phenotypic and functional characterization of MGE-derived spheroids from HC and FES iPSCs**

***Related to Figure 1***

(A) Immunostaining of neuronal and MGE progenitor markers in dissociated cells from 3-week-old spheroids. Yellow scale bar = 25 μm, white scale bar = 100 μm. (B) Immunostaining of MGE-derived cIN markers in dissociated cells from 8-week-old spheroids. Yellow scale bar = 25 μm, white scale bar = 100 μm. (C) Immunostaining of markers of other neuronal cells including astrocytes (GFAP), dopaminergic neurons (TH), oligodendrocytes (OLIG2), and microglia (IBA1). Yellow scale bar = 50 μm. (D) Schematic representation of migration from Matrigel-embedded spheroids *in vitro*, where 'Proximal' indicates migration near the spheroid border and 'Distal' refers to migration extending farther away from the spheroid. (E) Immunostaining of the transplanted cells *in vivo*. The yellow dashed line represents the edge of the graft, and the white dashed line indicates a 400 μm distance from the graft edge. White scale bar = 100 μm. (F) Impaired neural activities were indicated by Calcium imaging in the spheroids from the FES group. White scale bar = 200 μm. (G) Images show spheroid placement on MEA chips from HC and FES groups. Yellow scale bar = 200 μm.

**Figure S3. Overview of Data Quality, Integration, and Reproducibility of Single-Cell Transcriptomic Profiles**

*Related to Figure 2*

(A-B) t-SNE visualization representing the number of expressive detected genes (A, n = 2, 745) and total transcripts UMIs (B, n = 8, 906) in each cell. (C-D) Violin plots showing the number of detected genes (C) and total RNA transcripts (D) per cell in HC and FES groups. (E) Integration of transcriptome and cells of FES3 at 0W were colored by 10x Genomics and SCRB-seq. (F) Pearson’s correlation analysis of gene expression in successive batches from the same line (FES1) at weeks 3, 8, and 16. (G) Heatmap of hierarchical clustering based on Pearson’s correlation coefficients of gene expression across different time points (3W, 8W, 16W) and replicates (FES1A and FES1B). Color intensity represents correlation strength, with blue indicating lower and red indicating higher similarity.

**Figure S4. Extended data for gene expression and trajectory for scRNA-seq data**

***Related to Figure 2***

(A) Heatmap showing the expression of the top 100 DEGs among all cell types. Gene expression levels were averaged and scaled based on natural logarithm-scaled normalized counts. Color intensity ranges from blue (low expression) to red (high expression). (B) Heatmap showing the relative expression level of selected marker genes in nine cell types from the embryonic human brain. Human embryonic data are obtained from a published dataset.^52^ Color indicates relative gene expression levels, with red denoting high and blue denoting low expression. (C) The t-SNE visualization shows cell types at different differentiation stages. Cells are colored by cell types. (D) Bar plot showing the cell ratio of five cell types in each sample, with specimens from each developmental stage displayed adjacently. Cells are colored by cell types. (E) Scatter plot showing differential gene expression between FES and HC groups across all cell types. Each point represents a gene, with red indicating significantly upregulated genes and blue indicating significantly downregulated genes in the FES group (adjusted *p* < 0.05). (F) Bar plot showing the number of dysregulated genes within each cluster in the FES group. (G) Bar plot showing the number of shared DEGs across different clusters related to (F). (H) Overview of pseudotime trajectory inferred by Monocle2. Cells are colored by sampling time. (I) The pseudotime trajectory in Figure 2N was split by cell types. (J) Bubble plot showing the result of GO enrichment of dysregulated genes in the FES group along pseudotime. The top 10 terms were plotted based on the *P*-value. The *P*-value < 0.05 and the adjusted *P*-value were calculated using the Benjamini–Hochberg method.

**Figure S5. Extended data for transcriptomic characterization of the developing MGE-derived NPCs**

***Related to Figure 3***

(A-B) The t-SNE visualization shows cell subtypes of NPCs at different differentiation stages (A) and groups (B).Cells are colored by cell types.

**Figure S6. Extended data for transcriptomic characterization of the developing MGE-derived cINs**

***Related to Figures 4***

(A-H, J,K) t-SNE visualization showing the gene expression profiles of genes used for cINs classification, with red indicating high expression and blue indicating low expression. (I) Donut chart showing the cell proportion of stages assigned based on human prenatal MGE stages (GW09, GW12, GW13, GW16, GW18) in cINs across different in both HC and FES group. (L) The t-SNE plots show the cell subtypes of cINs at different differentiation stages. Cells are colored by cell types.

**Figure S7. Extended data on offspring deficits induced by gestational hypoxia and rescue by NAC**

***Related to Figures 5 and 6***

(A) Representative images of embryonic chains collected at embryonic days E12.5, E15.5, and E18.5 from normoxic and hypoxia-exposed pregnant mice. “#” indicate hypoxia-treated groups. (B) Measurement comparison of crown-rump length and maximum width of individual embryos from control and hypoxia groups at each developmental stage. Embryos were aligned along a millimeter ruler for visual assessment of size differences. (C-E) Quantification of embryonic width (C), crown–rump length (D) and body weight (E) at E12.5, E15.5, and E18.5 under normoxic and hypoxic conditions. For embryonic width and crown-rump length, n = 3 independent litters per group; for body weight, n = 8 independent litters per group. (F-G) Immunofluorescence staining (F) and quantification of VZ thickness (G) in the MGE at E12.5 and E15.5. Costaining of OLIG2 and DCX clearly delineates the VZ region, with yellow dashed lines indicating the VZ boundaries. n = 5 embryos from independent litters per group. White scale bar = 100 μm. (H-I) Bar plot showing the relative mRNA levels of *P27* in the ventral forebrain of embryonic mice at E12.5 (H) and E15.5 (I). Expression was normalized to *GAPDH*. n = 6 embryos from independent litters per group. (J–K) TUNEL staining analysis of apoptosis in the MGE region at E12.5, E15.5, and E18.5 under normoxic and hypoxic conditions. Arrows indicate TUNEL-positive cells. n = 6 embryos from independent litters per group. White scale bar = 100 μm. (L) Locomotor tracks of adult mice from control and hypoxia groups corresponding to behavioral quantification shown in Figures 5L–N. (M) Quantification of startle amplitude in adult offspring from control and hypoxia groups corresponding to Figure 5O. (N) Locomotor tracks of adult mice from control, hypoxia, low-dose NAC, and high-dose NAC groups corresponding to Figures 6I–K. (O) Quantification of startle amplitude in adult offspring from control + saline, hypoxia + saline, hypoxia + low-dose NAC, and hypoxia + high-dose NAC groups corresponding to Figure 6N. Detailed statistical results for these analyses are provided in Table S18.

**Figure S8. Effects of gestational hypoxia on maternal care and early postnatal pup outcomes**

***Related to Figure 5***
(A) Representative images showing nest-building performance in control and hypoxia-exposed dams at E18.5 and P1. (B) Quantification of nest-building scores at P1. Nest quality was scored using a 1–5 scale based on nest completeness, with higher scores indicating more complete and enclosed nest construction. (C) Number of pups per litter at P1. (D) Pup survival rate at P7. (E) Representative images showing milk bands in pups from control and hypoxia groups at P1. (F) Quantification of milk band scores at P1. Milk band scores were assigned as follows: 0, no visible milk band; 1, visible but small or poorly filled milk band; and 2, clearly filled milk band, indicating sufficient recent milk intake. (G) Latency to retrieve three pups at P1. (H) Latency to retrieve the first pup at P1. Data are presented as mean ± SEM. For milk band scores and pup retrieval latency, values were averaged within each litter before statistical analysis. Each dot represents one dam or one litter-averaged value. Statistical significance was determined by unpaired t-test. ns, not significant. n = 8 litters per group
