# Supplementary material for: Gestational Hypoxia Disrupts Medial Ganglionic Eminence Progenitor Dynamics and Interneuron Development in Schizophrenia
Source: Adv Sci (Weinh). 2026 Aug 12:e76936. Online ahead of print. doi: 10.1002/advs.76936 (PMC13464456; doi:10.1002/advs.76936)
Supplement: Supplementary file 2 [file ADVS-9999-e76936-s001.zip › Supplementary Figure 8.png]

Statistical methods for pairwise comparisons

Table/Figure number	Analysis Type	Group	Average	SEM	N	Statistical test used	Test Statistic	p value
**Table. S1**	Age	HC	13.17	0.75	6	Unpaired t-test	-0.27	0.7959
		FES	13.50	1.04	4			
	Gender	HC	Female/Male = 3/3		6	Chi-square test	0.02	0.8952
		FES	Female/Male = 3/1		4			
**Fig. 1G**	Proximal migratory cells percentage	HC	15.01	1.81	3 (mean per mouse)	Unpaired t-test	0.17	0.8761
		FES	15.75	4.11	3 (mean per mouse)			
**Fig. 1H**	Distal migratory cells percentage	HC	3.60	0.59	3 (mean per mouse)	Unpaired t-test	3.31	0.0296
		FES	1.31	0.36	3 (mean per mouse)			
**Fig. 1J**	Synaptic cleft (nm)	HC	13.85	2.16	3 (mean per organoid)	Unpaired t-test	3.76	0.0197
		FES	23.30	1.26	3 (mean per organoid)			
**Fig. 5D**	EdU$^+$/NKX2.1$^+$ in VZ at E12.5 (%)	Ctrl	80.06	1.46	6	Unpaired t-test	0.43	0.6731
		Hyp	81.19	2.16	6			
	BrdU$^+$/NKX2.1$^+$ in VZ at E12.5 (%)	Ctrl	74.78	2.01	6	Unpaired t-test	3.54	0.0054
		Hyp	65.07	1.87	6			
**Fig. 5E**	EdU$^+$/NKX2.1$^+$ in SVZ at E12.5 (%)	Ctrl	23.43	1.11	6	Unpaired t-test	0.75	0.4679
		Hyp	25.26	2.15	6			
	BrdU$^+$/NKX2.1$^+$ in SVZ at E12.5 (%)	Ctrl	18.67	1.27	6	Unpaired t-test	6.61	<0.0001
		Hyp	9.55	0.53	6			
**Fig. 5G**	EdU$^+$/NKX2.1$^+$ in VZ at E15.5 (%)	Ctrl	93.89	0.84	6	Unpaired t-test	5.91	0.0001
		Hyp	86.54	0.92	6			
	BrdU$^+$/NKX2.1$^+$ in VZ at E15.5 (%)	Ctrl	30.66	2.80	6	Unpaired t-test	5.23	0.0004
		Hyp	14.13	1.46	6			
**Fig. 5H**	EdU$^+$/NKX2.1$^+$ in SVZ at E15.5 (%)	Ctrl	41.10	1.66	6	Unpaired t-test	2.84	0.0176
		Hyp	35.28	1.21	6			
	BrdU$^+$/NKX2.1$^+$ in SVZ at E15.5 (%)	Ctrl	13.91	0.74	6	Unpaired t-test	10.92	<0.0001
		Hyp	4.66	0.41	6			
**Fig. 5J**	EdU$^+$BrdU$^-$/EdU$^+$ in VZ at E12.5 (%)	Ctrl	49.75	1.29	6	Unpaired t-test	6.84	<0.0001
		Hyp	62.42	1.33	6			

	EdU^+BrdU^-/EdU^+ in SVZ at E12.5 (%)	Ctrl	26.59	1.51	6	Unpaired t-test	12.15	<0.0001
		Hyp	65.39	2.82	6			
Fig. 5K	EdU^+BrdU^-/EdU^+ in VZ at E15.5 (%)	Ctrl	73.05	1.44	6	Unpaired t-test	4.14	0.0020
		Hyp	82.59	1.80	6			
	EdU^+BrdU^-/EdU^+ in SVZ at E15.5 (%)	Ctrl	88.11	1.14	6	Unpaired t-test	4.13	0.0020
		Hyp	93.15	0.42	6			
Fig. 5L	Distance of OFT	Ctrl	29.33	0.95	11	Unpaired t-test	1.24	0.2315
		Hyp	27.56	1.05	8			
Fig. 5M	Central entries of OFT	Ctrl	50.27	1.23	11	Unpaired t-test	2.48	0.0241
		Hyp	45.38	1.59	8			
Fig. 5N	Central distance of OFT	Ctrl	7.48	0.26	11	Unpaired t-test	2.83	0.0117
		Hyp	5.97	0.52	8			
Fig. 5Q	SST/mm2	Ctrl	16.40	0.83	7	Unpaired t-test	0.53	0.6055
		Hyp	17.49	1.81	7			
Fig. 5S	PV/mm2	Ctrl	53.29	2.15	7	Unpaired t-test	7.13	<0.0001
		Hyp	30.58	2.36	7			
Fig.S7H	Relative expression of P27 at E12.5	Ctrl	1.00	0.14	6	Unpaired t-test	3.52	0.0056
		Hyp	0.50	0.06	6			
Fig.S7I	Relative expression of P27 at E15.5	Ctrl	1.00	0.10	6	Unpaired t-test	6.61	<0.0001
		Hyp	0.32	0.04	6			
Fig.S7M	Startle amplitude (a.u.)	Ctrl	0.45	0.05	9	Unpaired t-test	0.11	0.9135
		Hyp	0.46	0.05	7			
Fig.S8B	Nest building score at P1	Ctrl	4.625	0.18	8	Unpaired t-test	0.51	0.6186
		Hyp	4.750	0.16	8			
Fig.S8C	Number of pups at P1	Ctrl	7.25	0.16	8	Unpaired t-test	1.79	0.0950
		Hyp	6.38	0.46	8			
Fig.S8D	Pup survival rate at P7 (%)	Ctrl	89.73	5.03	8	Unpaired t-test	1.52	0.1517
		Hyp	76.43	7.19	8			
Fig.S8F	Milk band score	Ctrl	1.46	0.19	8	Unpaired t-test	0.16	0.8737
		Hyp	1.41	0.18	8			
Fig.S8G	Latency to retrieve the first pup at P1 (s)	Ctrl	36.6	9.25	8	Unpaired t-test	0.20	0.8445
		Hyp	34.50	5.25	8			
Fig.S8H	Latency to retrieve three pups at P1 (s)	Ctrl	118.60	22.78	8	Unpaired t-test	1.25	0.2308
		Hyp	89.25	5.56	8			

Statistical methods for multiple group comparisons

Figure number	Analysis Type	Group	Average	SEM	N	Statistical test used	Comparisons	p value
**Fig. S1E-S1F**	mRNA expression of OCT4	H1	1.00	-	3	one-way ANOVA	-	-
		HC	1.67	0.21	16		vs hMSCs	0.0015
		FES	1.81	0.12	12		vs hMSCs	0.0008
		hMSCs	0.01	<0.01	3		-	-
	mRNA expression of NANOG	H1	1.00	-	3	one-way ANOVA	-	-
		HC	1.11	0.17	16		vs hMSCs	0.0119
		FES	0.95	0.09	12		vs hMSCs	<0.0442
		hMSCs	0.02	<0.01	3		-	-
**Fig. 6B**	$EdU^+/NKX2.1^+$ in VZ at E15.5 (%)	Ctrl+Saline	90.48	0.56	6	one-way ANOVA	vs Hyp+Saline	<0.0001
							vs Hyp+Low NAC	0.1454
							vs Hyp+High NAC	0.5485
		Hyp+Saline	81.28	0.46	6		vs Hyp+Low NAC	<0.0001
							vs Hyp+High NAC	<0.0001
		Hyp+Low NAC	88.81	0.61	6		-	-
		Hyp+High NAC	89.48	0.46	6		-	-
**Fig. 6C**	$BrdU^+/NKX2.1^+$ in VZ at E15.5 (%)	Ctrl+Saline	39.70	0.55	6	one-way ANOVA	vs Hyp+Saline	<0.0001
							vs Hyp+Low NAC	0.4085
							vs Hyp+High NAC	0.6327
		Hyp+Saline	13.78	0.27	6		vs Hyp+Low NAC	<0.0001
							vs Hyp+High NAC	<0.0001
		Hyp+Low NAC	41.00	0.64	6		-	-
		Hyp+High NAC	38.80	0.60	6		-	-
**Fig. 6D**	$EdU^+/NKX2.1^+$ in SVZ at E15.5 (%)	Ctrl+Saline	25.20	0.45	6	one-way ANOVA	vs Hyp+Saline	<0.0001
							vs Hyp+Low NAC	0.0070
							vs Hyp+High NAC	0.7460
		Hyp+Saline	10.66	0.45	6		vs Hyp+Low NAC	<0.0001
							vs Hyp+High NAC	<0.0001
		Hyp+Low NAC	23.00	0.15	6		-	-
		Hyp+High NAC	24.60	0.52	6		-	-
**Fig. 6E**	$BrdU^+/NKX2.1^+$ in SVZ at E15.5 (%)	Ctrl+Saline	22.59	0.55	6	one-way ANOVA	vs Hyp+Saline	<0.0001
							vs Hyp+Low NAC	0.8139
							vs Hyp+High NAC	0.0123
		Hyp+Saline	5.08	0.20	6		vs Hyp+Low NAC	<0.0001
							vs Hyp+High NAC	<0.0001
		Hyp+Low NAC	21.82	0.67	6		-	-
		Hyp+High NAC	25.50	0.83	6		-	-

Figure number	Analysis Type	Group	Average	SEM	N	Statistical test used	Comparisons	p value
**Fig. 6G**	$EdU^{+}BrdU^{-}$/EdU^{+} in VZ at E15.5 (%)	Ctrl+Saline	74.17	0.43	6	one-way ANOVA	-	-
		Hyp+Saline	82.23	0.72	6		vs Ctrl+Saline	<0.0001
		Hyp+Low NAC	79.85	1.11	6		vs Ctrl+Saline	0.0004
		Hyp+High NAC	71.86	0.52	6		vs Ctrl+Saline	0.2070
**Fig. 6H**	$EdU^{+}BrdU^{-}$/EdU^{+} in SVZ at E15.5 (%)	Ctrl+Saline	73.44	1.19	6	one-way ANOVA	-	-
		Hyp+Saline	86.41	0.55	6		vs Ctrl+Saline	<0.0001
		Hyp+Low NAC	72.85	1.07	6		vs Ctrl+Saline	>0.9999
		Hyp+High NAC	71.57	0.78	6		vs Ctrl+Saline	0.6828
**Fig. 6I**	Distance of OFT	Ctrl+Saline	22.43	1.62	6	one-way ANOVA	-	-
		Hyp+Saline	26.07	0.73	6		vs Ctrl+Saline	0.2625
		Hyp+Low NAC	26.15	0.91	6		vs Ctrl+Saline	0.2459
		Hyp+High NAC	27.37	1.59	6		vs Ctrl+Saline	0.0797
**Fig. 6J**	Central entries of OFT	Ctrl+Saline	15.50	1.80	6	one-way ANOVA	-	
		Hyp+Saline	9.17	0.70	6		vs Ctrl+Saline	0.0162
		Hyp+Low NAC	16.50	0.99	6		vs Ctrl+Saline	0.9519
		Hyp+High NAC	12.67	1.48	6		vs Ctrl+Saline	0.4621
**Fig. 6K**	Central distance of OFT	Ctrl+Saline	4.86	0.75	6	one-way ANOVA	-	-
		Hyp+Saline	2.72	0.37	6		vs Ctrl+Saline	0.0269
		Hyp+Low NAC	5.63	0.41	6		vs Ctrl+Saline	0.6791
		Hyp+High NAC	3.18	0.11	6		vs Ctrl+Saline	0.1043
**Fig. 6N**	PV/mm2	Ctrl+Saline	53.63	3.48	12	one-way ANOVA	-	-
		Hyp+Saline	42.28	1.66	12		vs Ctrl+Saline	0.0162
		Hyp+Low NAC	51.34	1.81	12		vs Ctrl+Saline	0.9519
		Hyp+High NAC	56.50	3.59	12		vs Ctrl+Saline	0.4621
**Fig.S7O**	Startle amplitude (a.u.)	Ctrl+Saline	0.45	0.03	6	one-way ANOVA	-	-
		Hyp+Saline	0.40	0.01	6		vs Ctrl+Saline	0.7607
		Hyp+Low NAC	0.39	0.04	6		vs Ctrl+Saline	0.7130
		Hyp+High NAC	0.40	0.06	6		vs Ctrl+Saline	0.7880

Statistical methods for multiple-factor comparisons

Figure number	Assay	Comparision	Time point	Correction method	Adjusted *p* value	Significant after Correction
Fig. 1E	Proximal migration comparison	HC vs FES	Day 3	Šídák	*p* adj = 0.0190	Yes
	Proximal migration comparison	HC vs FES	Day 6	Šídák	*p* adj ＜ 0.0001	Yes
	Proximal migration comparison	HC vs FES	Day 7	Šídák	*p* adj ＜ 0.0001	Yes
Fig. 1F	Distal migration comparison	HC vs FES	Day 3	Šídák	*p* adj = 0.3812	No
	Distal migration comparison	HC vs FES	Day 6	Šídák	*p* adj = 0.0025	Yes
	Distal migration comparison	HC vs FES	Day 7	Šídák	*p* adj ＜ 0.0001	Yes
Fig. 1L	Number of active electrodes	HC vs FES	Day 160	Šídák	*p* adj = 0.1456	No
	Number of active electrodes	HC vs FES	Day 163	Šídák	*p* adj = 0.0392	Yes
	Number of active electrodes	HC vs FES	Day 166	Šídák	*p* adj = 0.0583	No
	Number of active electrodes	HC vs FES	Day 169	Šídák	*p* adj = 0.0321	Yes
	Number of active electrodes	HC vs FES	Day 174	Šídák	*p* adj = 0.0251	Yes
Fig. 1M	Weighted mean firing rate	HC vs FES	Day 160	Šídák	*p* adj = 0.6536	No
	Weighted mean firing rate	HC vs FES	Day 163	Šídák	*p* adj = 0.3417	No
	Weighted mean firing rate	HC vs FES	Day 166	Šídák	*p* adj = 0.7264	No
	Weighted mean firing rate	HC vs FES	Day 169	Šídák	*p* adj = 0.4685	No
	Weighted mean firing rate	HC vs FES	Day 174	Šídák	*p* adj = 0.8189	No
Fig. 1O	Synchrony index	HC vs FES	Day 160	Šídák	*p* adj = 0.1891	No
	Synchrony index	HC vs FES	Day 163	Šídák	*p* adj = 0.1621	No
	Synchrony index	HC vs FES	Day 166	Šídák	*p* adj = 0.1738	No
	Synchrony index	HC vs FES	Day 169	Šídák	*p* adj = 0.0374	Yes
	Synchrony index	HC vs FES	Day 174	Šídák	*p* adj = 0.1750	No
Fig. 5O	74 db of PPI	Ctrl vs Hyp	6-8 week	Bonferroni	*p* adj = 0.4354	No
	78 db of PPI	Ctrl vs Hyp		Bonferroni	*p* adj = 0.1118	No

Figure number	Assay	Comparision	Time point	Correction method	Adjusted *p* value	Significant after Correction
	82 db of PPI	Ctrl vs Hyp		Bonferroni	*p* adj = 0.0450	Yes
**Fig. 6L**	74 db of PPI	Ctrl+Saline vs Hyp+Saline	6-8 week	Bonferroni	*p* adj = 0.2058	No
		Ctrl+Saline vs Hyp+Low NAC		Bonferroni	*p* adj = 0.7878	No
		Ctrl+Saline vs Hyp+High NAC		Bonferroni	*p* adj = 0.0711	No
	78 db of PPI	Ctrl+Saline vs Hyp+Saline		Bonferroni	*p* adj = 0.0151	Yes
		Ctrl+Saline vs Hyp+Low NAC		Bonferroni	*p* adj = 0.2335	No
		Ctrl+Saline vs Hyp+High NAC		Bonferroni	*p* adj = 0.0684	No
	82 db of PPI	Ctrl+Saline vs Hyp+Saline		Bonferroni	*p* adj = 0.0322	Yes
		Ctrl+Saline vs Hyp+Low NAC		Bonferroni	*p* adj = 0.8284	No
		Ctrl+Saline vs Hyp+High NAC		Bonferroni	*p* adj = 0.2399	No
**Fig.S7C**	Width	Ctrl vs Hyp	E12.5	Šídák	*p* adj = 0.8982	No
	Width	Ctrl vs Hyp	E15.5	Šídák	*p* adj = 0.9996	No
	Width	Ctrl vs Hyp	E18.5	Šídák	*p* adj = 0.9781	No
**Fig.S7D**	Length	Ctrl vs Hyp	E12.5	Šídák	*p* adj = 0.0398	Yes
	Length	Ctrl vs Hyp	E15.5	Šídák	*p* adj = 0.0279	Yes
	Length	Ctrl vs Hyp	E18.5	Šídák	*p* adj = 0.1566	No
**Fig.S7E**	Weight	Ctrl vs Hyp	E12.5	Šídák	*p* adj = 0.0056	Yes
	Weight	Ctrl vs Hyp	E15.5	Šídák	*p* adj = 0.0321	Yes
	Weight	Ctrl vs Hyp	E18.5	Šídák	*p* adj =0.5101	No
**Fig.S7G**	Thickness of VZ	Ctrl vs Hyp	E12.5	Šídák	*p* adj = 0.9769	No
	Thickness of VZ	Ctrl vs Hyp	E15.5	Šídák	*p* adj = 0.9854	No
**Fig.S7K**	Tunel$^+$ in MGE	Ctrl vs Hyp	E12.5	Šídák	*p* adj = 0.9996	No
	Tunel$^+$ in MGE	Ctrl vs Hyp	E15.5	Šídák	*p* adj = 0.9999	No
	Tunel$^+$ in MGE	Ctrl vs Hyp	E18.5	Šídák	*p* adj = 0.9957	No

Table S18. Methods of statistical analysis.
(Related to Method section).
