# Supplementary material for: Gestational Hypoxia Disrupts Medial Ganglionic Eminence Progenitor Dynamics and Interneuron Development in Schizophrenia
Source: Adv Sci (Weinh). 2026 Aug 12:e76936. Online ahead of print. doi: 10.1002/advs.76936 (PMC13464456; doi:10.1002/advs.76936)
Supplement: Supplementary file 3 [file ADVS-9999-e76936-s004.zip › Supplementary Table/Table S17. Key resources.docx]

| **REAGENT or RESOURCE** | **SOURCE** | **IDENTIFIER** |
| --- | --- | --- |
| **Antibodies** | | |
| Rat anti-BrdU | Abcam | Cat# AB6326 |
| Rabbit anti-OCT4 | Cell Signaling Technology | Cat# 2750 |
| Mouse anti-TRA-1–60 | Abcam | Cat# AB16288 |
| Mouse anti-SSEA-4 | BD Biosciences | Cat# 560128 |
| Mouse anti-TRA-1–81 | BD Biosciences | Cat# 560161 |
| Rabbit anti-SOX2 | Cell Signaling Technology | Cat# 3579 |
| Rabbit anti- NKX2.1 | Abcam | Cat# AB227574 |
| Mouse anti-NESTIN | Cell Signaling Technology | Cat# 33475S |
| Rabbit anti-GABA | Immunostar | Cat# 20094 |
| Mouse anti-GAD67 | Millipore | Cat# MAB5406 |
| Mouse anti-β-tubulin | BD Biosciences | Cat# 560381 |
| Rabbit anti-SOX6 | Thermo Fisher Scientific | Cat# AB5805 |
| Mouse anti-hNuclei | Millipore | Cat# MAB1281 |
| Mouse anti-OLIG2 | Sigma-Aldrich | Cat# MABN50 |
| Mouse anti-GFAP | Neuromics | Cat# MO15052 |
| Rabbit anti-TH | Pelfreez | Cat# P40101-150 |
| Rabbit anti-IBA-1 | FUJIFILM | Cat# 019-19741 |
| Guinea pig anti-DCX | Sigma-Aldrich | Cat# AB2253 |
| Rabbit anti-SST | Invitrogen | Cat# PA5-85759 |
| Rabbit anti-PV | Cell Signaling Technology | Cat# 80561 |
| Rabbit anti-HIF-1α | Cell Signaling Technology | Cat# 36169S |
| Alexa Fluor 555-labeled Goat anti-Rabbit IgG (H+L) | Thermo Fisher Scientific | Cat# A32732 |
| Alexa Fluor 488-labeled Goat anti-Guinea pig IgG (H+L) | Thermo Fisher Scientific | Cat# A11073 |
| Alexa Fluor 488-labeled Donkey anti-Mouse IgG (H+L) | Thermo Fisher Scientific | Cat# A21202 |
| **Chemicals, peptides, and recombinant proteins** | | |
| A-83-01 | Stemgent | Cat# 040014 |
| Ascorbic acid | Sigma-Aldrich | Cat# A8960 |
| B-27™ Supplement (50×), serum-free | Thermo Fisher Scientific | Cat# 17504-044 |
| β-mercaptoethanol | Thermo Fisher Scientific | Cat# 21985-023 |
| Basic fibroblast growth factor | PeproTech | Cat# 100-18B |
| Bovine serum | Sigma-Aldrich | Cat# A9647 |
| Brain-derived neurotrophic factor | ProSpec Bio | Cat# cyt-207-b |
| BrdU | Sigma-Aldrich | Cat# B5002 |
| CHIR99021 | Stemgent | Cat# S2924 |
| Click-iT Edu Cell Proliferation Imaging Kit with Alexa Fluor 647 dye | Thermo Fisher Scientific | Cat# 10340 |
| Colchicine | Sangon Biotech | Cat# 64-86-8 |
| DAPI | Invitrogen | Cat# 62248 |
| DMEM | Thermo Fisher Scientific | Cat# 11965-092 |
| DMEM/F12 | Thermo Fisher Scientific | Cat# 10565-018 |
| Essential 8 Medium | Thermo Fisher Scientific | Cat# A1517001 |
| EX-CYTE Growth Enhancement Media Supplement | Millipore | Cat# 811292 |
| Fetal Bovine Serum | Thermo Fisher Scientific | Cat# 26140-079 |
| FGF8 | ProSpec Bio | Cat# cyt-839-c |
| Fibronectin | Corning | Cat#356008 |
| Fluoromount-G | Southern Biotech | Cat# 0100-01 |
| FMS-like tyrosine kinase 3 ligand | PeproTech | Cat# 250-31L |
| Giemsa staining | Sigma-Aldrich | Cat# G5637 |
| Glacial acetic acid | Sangon Biotech | Cat# 64-19-7 |
| Glial cell-derived neurotrophic factor | ProSpec Bio | Cat# cyt-305-b |
| GlutaMAX | Thermo Fisher Scientific | Cat# 35050-061 |
| Glutaraldehyde (pH 7.4) | SPI-chem | Cat# 111-30-8 |
| HA-100 | Santa Cruz Biotechnology | Cat# SC-203072 |
| HCl | Sinopharm | Cat# 7647-01-0 |
| Human leukemia inhibitory factor | Millipore | Cat# LIF1010 |
| Interleukin 3 | PeproTech | Cat# 200-03 |
| Interleukin 6 | PeproTech | Cat# 200-06 |
| Isoflurane | RWD | Cat# R510-22-10 |
| Inhibitor of Wnt Production 2 | Selleck Chem | Cat# s7085 |
| KCl | Sangon Biotech | Cat# A610440 |
| Knockout Serum Replacement | Thermo Fisher Scientific | Cat# 10828-028 |
| Laminin | Sigma-Aldrich | Cat# L6274 |
| LDN193189 | Selleck Chem | Cat# S2618 |
| Lead citrate | Ted Pella Inc Reading | Cat# 19312 |
| Matrigel | Corning | Cat# 354237 |
| Maxima SYBR Green | Thermo Fisher Scientific | Cat# K0222 |
| Methanol | Sangon Biotech | Cat# 67-56-1 |
| N2 Supplement | Thermo Fisher Scientific | Cat# 17502-048 |
| NAC | MedChemExpress | Cat# 616-91-1 |
| Neurobasal media | Thermo Fisher Scientific | Cat# 21103-049 |
| Optimum cutting temperature Compound | Sakura | Cat# 4583 |
| Osmic acid | Ted Pella Inc Reading | Cat# 18459 |
| Paraformaldehyde | BBI Life Sciences | Cat# E672002-0500 |
| PD0325901 | Stemgent Lexington | Cat# 040006 |
| Phosphate buffered saline | Thermo Fisher Scientific | Cat# 10010023 |
| Polyethyleneimine | Sigma-Aldrich | Cat# 408727 |
| Quercetin | Sigma-Aldrich | Cat# 1002933906 |
| SAG | Selleck Chem | Cat# S7779 |
| SB431542 | MEDCHEM | Cat# Axon 1661 |
| Soluble Interleukin 6 Receptor | PeproTech | Cat# 200-06RC |
| Stem Cell Factor | PeproTech | Cat# 255-SC |
| Sodium borate | Sinopharm | Cat# 1330-43-4 |
| StemSpan SFEM media | STEMCELL Technologies | Cat# 09650 |
| Trehalose | Thermo Fisher Scientific | Cat# A19434 |
| Tribromoethanol | AibeiBio | Cat# M2910 |
| TRIzol | Invitrogen | Cat# 15596026 |
| Triton-X-100 | Beyotime | Cat# P0096 |
| Trypsin-EDTA | Thermo Fisher Scientific | Cat# 15400054 |
| Thrombopoietin | PeproTech | Cat# 300-18 |
| TUNEL Apoptosis Detection Kit | Beyotime | Cat# C1088 |
| Turbo DNase | Thermo Fisher | Cat# AM2238 |
| ROCK inhibitor, Y27632 | Apex Bio | Cat# A3008 |
| Uranyl acetate | Ted Pella Inc Reading | Cat# 19481 |
| Versene Solution | Thermo Fisher Scientific | Cat# 15040066 |
| **Oligonucleotides** |  |  |
| OCT 4, forward primer | 19 bp | CAGTGCCCGAAACCCACAC |
| OCT 4, reverse primer | 20 bp | GGAGACCCAGCAGCCTCAAA |
| NANOG, forward primer | 20 bp | CAGAAGGCCTCAGCACCTAC |
| NANOG, reverse primer | 20 bp | ATTGTTCCAGGTCTGGTTGC |
| P27, forward primer | 20 bp | AGATACGAGTGGCAGGAGGT |
| P27, reverse primer | 20 bp | TGTTTACGTCTGGCGTCGAA |
| GAPDH, forward primer | 20 bp | GGTTGTCTCCTGCGACTTCA |
| GAPDH, reverse primer | 21 bp | TGGTCCAGGGTTTCTTACTCC |
| GEBNAL, forward primer | 22 bp | CAGTTCCTCGCCTTAGGTTGTA |
| GEBNAL, reverse primer | 22 bp | GGCTAGGAGTCACGTAGAAAGG |
| Primers-1 used for Mycoplasm test, forward primer | 20 bp | ACACCATGGGAGCTGGTAAT |
| Primers-1 used for Mycoplasm test, reverse primer | 27 bp | CTTCATCGACTTTCAGACCCAAGGCAT |
| Primers-2 used for Mycoplasm test, forward primer | 18 bp | GTTCTTTGAAAACTGAAT |
| Primers-2 used for Mycoplasm test, reverse primer | 18 bp | GCATCCACCAAAAACTCT |
| **Deposited data** |  |  |
| Single-cell RNA sequencing dataset | This study | GEO: GSE217934 |
| Single-cell RNA sequencing dataset (reference) | Shi et al.51 | GEO: GSE135827 |
| Single-cell RNA sequencing dataset (reference) | Yu et al.52 | GEO: [GSE165388](https://www.ncbi.nlm.nih.gov/geo/query/acc.cgi?acc=GSE165388) |
| **Software and algorithms** |  |  |
| Axion Integrated Studio (AxIS 2.4) | Axion Biosystems | https://www.axionbiosystems.com/products/software/axis-software |
| Cell Ranger (v3.0.2) | 10x Genomics | https://support.10xgenomics.com/single-cell-gene-expression/software/  overview/welcome |
| CNVkit (v0.9.8) | Eric Talevich et al. | https://cnvkit.readthedocs.io/en/stable/ |
| Cutadapt (v1.15) | Marcel Martin | https://cutadapt.readthedocs.io/en/stable/ |
| GraphPad Prism 9 | GraphPad Software | https://www.graphpad.com/scientific-software/prism/ |
| HTSeq (v0.11.2) | Simon Anders et al. | https://htseq.readthedocs.io/ |
| ImageJ | NIH | https://imagej.nih.gov/ij/ |
| LUMPY (v0.2.13) | Ryan M Layer et al. | https://github.com/arq5x/lumpy-sv |
| MAGMA | CTG Lab | https://ctg.cncr.nl/software/magma |
| MATLAB 7.2 | MathWorks | https://www.mathworks.com/products/matlab.html |
| NeuroExplorer | Nex Technologies | https://www.neuroexplorer.com/ |
| R (Version 4.3.2) | The R Foundation | https://www.r-project.org/ |
| RStudio | RStudio, PBC | https://www.rstudio.com/ |
| STAR (v2.6.0a) | Alexander Dobin | https://github.com/alexdobin/STAR |
| ZEN Image Analysis Software | Carl Zeiss | https://www.zeiss.com/microscopy/int/products/microscope-software/ zen.html |

**Table S17. Key resources**

**(Related to Method section).**
