# Supplementary material for: Gestational Hypoxia Disrupts Medial Ganglionic Eminence Progenitor Dynamics and Interneuron Development in Schizophrenia
Source: Adv Sci (Weinh). 2026 Aug 12:e76936. Online ahead of print. doi: 10.1002/advs.76936 (PMC13464456; doi:10.1002/advs.76936)
Supplement: Supplementary file 3 [file ADVS-9999-e76936-s004.zip › Supplementary Table/Table S18. Statistical analysis methods.pdf]

https://articlereview.paperperfect.ai/#/?templateID=6b12c2fba1874ac6b68ff57fc3e03308155713601

Author Queries

Q1 AUTHOR: Please confirm that forenames/given names (blue), surnames/family names (vermilion), and corresponding author(s) have been identified correctly.
Confirmed. No errors were found.

Q2 AUTHOR: Please confirm that the funding information correctly extracted from your article: that it includes all funders and that the text has been matched to the correct FundRef Registry organization names. If a name was not found in the FundRef registry, it may not be the canonical name form, it may be a program name rather than an organization name, or it may be an organization not yet included in FundRef Registry. If you know of another name form or a parent organization name for a not found item on this list below, please share that information.
The funding information has been updated to reflect the full and accurate names of the funding organizations, and an additional funding source has been included to ensure the completeness of the funding statement. The updated funding information is accurate and complete.

Q3 AUTHOR: Please provide the department name for all affiliations.
The affiliation information has been checked and confirmed to accurately represent the authors' institutional affiliations. The relevant departmental/academic unit information has been provided where applicable.

Article DOI : 10.1002/advs.76936

RESEARCH ARTICLE

Gestational Hypoxia Disrupts Medial Ganglionic Eminence Progenitor Dynamics and Interneuron Development in Schizophrenia

Peiyan Ni, Qiancheng Zhang, and Youhui Jiang contributed equally to this work.

Peiyan Ni [1,2,3,4] | Qiancheng Zhang [5,6,7,8] | Youhui Jiang [1,2,3] | Huiying Yao [9] | Chuqing Zhou [1,2,3] | Xiaoyi Tian [1,2,3] | Shiyingnan Gao [10] | Jun Zhao [1,2,3] | Xinyi Ren [1,2,3] | Naxin Xu [1,2,3] | Mengshan Fu [1,2,3] | Liansheng Zhao [11] | Xueyu Qi [1,2,3] | Xueli Yu [1,2,3] | Wanjun Guo [1,2,3] | Sangmi Chung [12] | Fan Guo [5,6,7,~~13,~~8] | Tao Li [1,2,3,4,13]

[1] Affiliated Mental Health Center & Hangzhou Seventh People's Hospital, Zhejiang University School of Medicine, Hangzhou, Zhejiang, China

[2] Zhejiang Key Laboratory of Clinical and Basic Research on Psychiatric Diseases, Hangzhou, Zhejiang, China

[3] Zhejiang Engineering Research Center for Intelligent Diagnosis and Treatment of Mental and Psychological Disorders, Hangzhou, Zhejiang, China

[4] The MOE Frontier Science Center for Brain Science & Brain-Machine Integration, Zhejiang University, Hangzhou, China

[5] State Key Laboratory of Stem Cell and Reproductive Biology, Institute of Zoology, University of Chinese Academy of Sciences, Chinese Academy of Sciences, Beijing, China

[6] Institute For Stem Cell and Regeneration, Chinese Academy of Sciences, Beijing, China

[7] Beijing Institute for Stem Cell and Regenerative Medicine, Beijing, China

[8] Key Laboratory of Organ Regeneration and Reconstruction, Chinese Academy of Sciences, Beijing, China

[9] College of Life Sciences, Sichuan University, Chengdu, Sichuan, China

[10] School of Mental Health, Wenzhou Medical University, Wenzhou, Zhejiang, China

[11] The Mental Health Center and Psychiatric Laboratory, West China Hospital, Sichuan University, Chengdu, Sichuan, China

[12] Department of Neurosurgery, Rutgers University, Piscataway, NJ, USA

[13] Nanhu Brain-Computer Interface Institute, Hangzhou, Zhejiang, China

Funding Information

National Natural Science Foundation of China : 82571737 : 82230046

Key R&D Program of Zhejiang : 2025C02112

~~Hangzhou Science and Technology Bureau~~ the Key R&D by Hangzhou Science and Technology Bureau : 20241203A14

Hangzhou Special Project for Biomedical and Health Industry : 2024WJC035

~~Basic Research for Psychiatric Diseases~~ Zhejiang Key Laboratory of Clinical and Basic Research for Psychiatric Diseases : {{Tao Li: One funding source was inadvertently omitted from the accepted manuscript. We have now added the relevant funding information to the Funding section. }}2024ZY01010 : 2024E10107

~~Medical Disciplines of Hangzhou~~ the Construction Fund of Medical Disciplines of Hangzhou : 2025HZGF10

National Institute of Mental Health : MH133205 : MH131610

Copyright | OPEN ACCESS

© 2026 The Author(s). *Advanced Science* published by Wiley-VCH GmbH.

This is an open access article under the terms of the Creative Commons Attribution License, which permits use, distribution and reproduction in any medium, provided the original work is properly cited.

Received Date: 12 December 2025 |
Revised Date: 20 July 2026 |
Accepted Date: 23 July 2026

Abstract

Schizophrenia (SCZ) is a neurodevelopmental disorder characterized by heterogeneous symptoms and multifactorial etiologies. Medial ganglionic eminence (MGE) spheroids generated from first-episode schizophrenia (FES) patients revealed accelerated neurodevelopmental trajectories and enhanced hypoxia responses via single-cell transcriptomics. Notably, FES patient-derived MGE spheroids exhibited defective interneuron migration, disrupted synaptic ultrastructure, and diminished network synchronization. To establish causal links, the gestational hypoxia mouse model recapitulated key pathologies, including reduced progenitor proliferation, abbreviated cell cycles, mismatched interneuron subtypes, and schizophrenia-like behavioral deficits in offspring. Critically, maternal administration of N-acetylcysteine (NAC) restored redox homeostasis and rescued both cellular and behavioral phenotypes. Collectively, these results demonstrate that developmental redox disruption directly impairs GABAergic circuit assembly, while supporting targeted

antioxidant pharmacotherapy during gestation as a translatable strategy to mitigate neurodevelopmental risk.

Graphical Abstract

FES-derived MGE spheroids exhibit progenitor-stage alterations in developmental trajectory and hypoxia-responsive transcriptional programs, followed by functional disruption. Gestational hypoxia recapitulates impaired progenitor proliferation, shortened cell-cycle progression, interneuron developmental abnormalities, and schizophrenia-like behaviors in mice, whereas maternal NAC supplementation restores redox homeostasis and rescues key cellular and behavioral phenotypes.

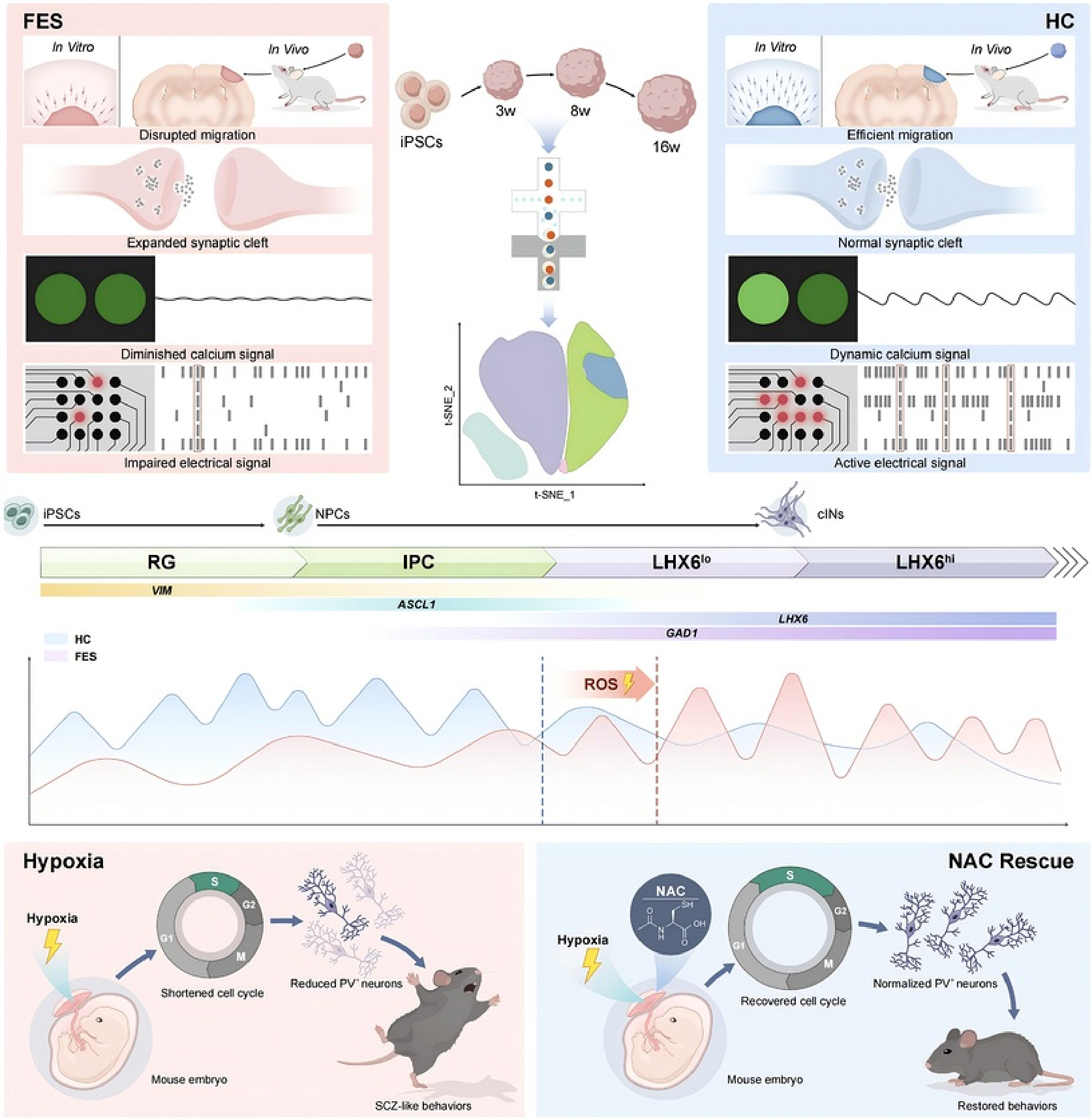

Keywords

gestational hypoxia | interneuron development | medial ganglionic eminence | N-acetylcysteine | schizophrenia

1 | Introduction

Schizophrenia (SCZ) has been widely considered a neurodevelopmental disorder with a prevalence of 1% of the global adult population [1], presenting a considerable burden to both individuals and society. SCZ is characterized by a spectrum of symptoms, including positive symptoms (e.g., delusions, hallucinations), negative symptoms (e.g., apathy, anhedonia), and cognitive symptoms (e.g., impaired memory, executive function, and attention) [2]. Prevailing evidence implicates that the emergence of negative symptoms and cognitive deficits can be attributed to the excitatory-inhibitory imbalance during the neurodevelopmental process [2]. This imbalance is partly driven by a diminished functionality of inhibitory interneurons, particularly within the cortical regions [3, 4]. Cortical interneurons (cINs) are characterized by a spectrum of biological functions contributing to the elaborate excitatory-inhibitory circuits involved in the cortex [5–9]. The cINs have been

increasingly acknowledged for their pivotal role as a neuronal population consistently affected in the pathophysiology of SCZ [10–14]. In the early stages of neurodevelopment, the vast majority of cINs are primarily generated in germinal zones of the medial ganglionic eminence (MGE) in the subpallium and progressively differentiate into young GABAergic interneurons from nascent progenitors (*NKX2.1*$^{+}$/*VIM*$^{+}$) [15]. These interneurons undergo an extended migration that spans several months, subsequently experience activity-dependent refinement, and eventually integrate into the sophisticated cortical circuits [16]. This represents a critical phase in the functional maturation of cINs, where they transform into a spectrum of functional subtypes. Prominent among these are the somatostatin-positive (SST^{+}) and parvalbumin-positive (PV^{+}) interneurons [17, 18], highlighting the intricate cellular diversity that emerges during this developmental phase.

The development of cINs is typically prolonged and tightly orchestrated, rendering these cells highly susceptible to in utero environmental perturbations. Among various prenatal risk factors associated with SCZ, hypoxia has garnered particular attention as a potential convergent mechanism [19–21]. Hypoxia-induced oxidative stress arises from an imbalance between reactive oxygen species (ROS) production and antioxidant defense systems, and is frequently accompanied by mitochondrial dysfunction [22]. This redox imbalance can disrupt multiple neurodevelopmental processes, including progenitor proliferation and differentiation, cell cycle regulation, neuronal migration, axon guidance, and synaptogenesis [23–26]. Notably, PV^{+} interneurons appear to be especially vulnerable to oxidative stress. Owing to their high metabolic demands required to sustain fast-spiking activity and extensive synaptic output throughout maturation, even minor metabolic disturbances or impairments in antioxidant systems can compromise their survival and function [27, 28]. Clinical studies have also reported reduced antioxidant enzyme levels and dysregulated glutathione systems in SCZ patients, further implicating oxidative stress in the disease etiology [29–32].

Understanding how prenatal oxidative stress impacts interneuron lineage commitment and cortical circuit assembly could therefore be essential for providing critical insight into the developmental origins of SCZ. However, the limited availability of accessible live informative human brain has historically impeded our understanding of the underlying mechanisms of SCZ pathogenesis. Although postmortem studies have provided critical insights, their interpretability is often confounded by factors such as antipsychotic exposure and disease chronicity [33, 34]. Induced pluripotent stem cell (iPSC) technology has emerged as a transformative platform for modeling patient-specific neurodevelopment, especially considering SCZ's early-life origins [35–40]. Recent advances in iPSC-derived protocols enable the generation of MGE-like spheroids, which faithfully recapitulate the generation and maturation of cINs [41–47]. These spheroids encompass cell types and developmental states derived from the MGE lineage, but bulk transcriptomics often masks subtle lineage-specific alterations. Single-cell RNA sequencing (scRNA-seq) overcomes this limitation by resolving cellular heterogeneity and reconstructing developmental trajectories through pseudotime analysis [48–50]. Recent scRNA-seq studies of human fetal brains have unveiled the remarkable diversity in molecular and transcriptional profiles of MGE-derived cells, shedding new light on the cINs development [51, 52]. To date, no studies have specifically explored the nuanced differences at the single-cell level in MGE-derived cINs between individuals with First-Episode Schizophrenia (FES) and healthy controls (HC), with a particular emphasis on the intricate regulatory mechanisms that govern cell differentiation, lineage determination, migration, and synaptic plasticity. Unraveling these intricate regulatory processes could substantially enhance our comprehension of the pathophysiological underpinnings of SCZ.

In this study, iPSCs were generated from ten individuals, including four patients with FES and six HC. Using these cell lines, MGE-derived spheroids were derived and profiled with the scRNA-seq approach to map the developmental trajectories of cINs in vitro and characterize cellular heterogeneity between the FES and HC groups. Despite comparable early cell type compositions, pseudotime analysis revealed accelerated cIN maturation in patient-derived samples. However, functional assays indicated impaired maturation, suggesting a disruption in developmental timing, potentially driven by intrinsic or environmental factors. Additionally, persistent redox imbalance was detected throughout the differentiation from neural progenitor cells (NPCs) to postmitotic cINs. To investigate causality, a gestational hypoxia mouse model was established and their progenitor behavior was examined in the embryonic MGE. 5-ethynyl-2′-deoxyuridine (EdU) and 5-bromo-2′-deoxyuridine (BrdU) dual-pulse labeling revealed reduced proliferation and shortened cell cycles in hypoxia-exposed embryos, indicating stress-induced acceleration of neurogenesis. This developmental disturbance resulted in impaired interneuron generation and SCZ-relevant behavioral phenotypes in offspring. Strikingly, maternal administration of the antioxidant N-acetylcysteine (NAC) restored MGE progenitor cell cycle dynamics, corrected interneuron fate specification, and rescued adult behavioral deficits. Together, these findings demonstrate that gestational oxidative stress disrupts cIN development by altering progenitor cell cycle kinetics, providing a developmental framework for SCZ pathogenesis and identifying antioxidant intervention as a potential preventive strategy.

2 | Results

2.1 | Impaired Oxygen-Consuming Functions in Schizophrenia-Derived MGE Spheroids

The demographic and clinical characteristics of all participants are shown in Table S1. To minimize potential confounding effects of ethnicity, environmental factors, and prior pharmacotherapy, all patients were treatment-naïve Han Chinese adolescents experiencing their first episode of SCZ (13.50 ± 2.08 years, 1 Male/3 Females). Han Chinese adolescents without psychiatric disorders (13.17 ± 1.84 years, 3 males/3 females) were recruited as HC, with no significant differences in age ($t = 0.27$, $p = 0.80$) or gender ($\chi^2 = 0.02$, $p = 0.90$) compared to the patient group. All the iPSCs were derived from the subject-donated peripheral blood mononuclear cells (PBMCs) by the

Episomal method, with all detected short tandem repeat (STR) sites accurately matched (Table S2). All iPSC lines utilized were mycoplasma-free, pathogen-negative (Table S3), and karyotype-normal (Figure S1A). As shown in Figure S1B, all reprogrammed iPSCs showed embryonic stem-like cells (ESC-like) morphology. The expected high expression level of pluripotent markers was determined by immunocytochemistry staining (OCT4 and TRA-1-60, Figure S1B), flow cytometry analysis (SSEA4 and TRA-1-81, Figure S1C,D), and quantitative real-time PCR (qPCR) (*OCT4* and *NANOG*) (Figure S1E,F). In vivo pluripotency assessment by teratoma formation assay demonstrated that all cell lines differentiated into derivatives of the three germ layers (Figure S1B). Exogenous plasmid integration was excluded by qPCR detection (< 0.1 copies/cell, Figure S1G). To assess genomic background and sample consistency, whole-genome sequencing (WGS) was performed on PBMCs and matched iPSCs from 10 individuals. Principal component analysis (PCA) confirmed that samples clustered by individual, supporting high-fidelity reprogramming and sample identity (Figure S1H). The number of singleton mutations varied across individuals [32–90]. While several genes altered in the FES group (e.g., *GRIN2C*, *MAGI2*, *SHANK3*) have been previously reported to link to psychiatric risk, [13, 53, 54] no consistent group-specific mutations were identified (Figure S1I, Table S4). These findings suggest no intra-group enrichment of known SCZ risk variants. Parallel coverage of six exogenous genes confirmed the absence of residual vector sequences (Table S5). Collectively, these results demonstrate that the high-quality iPSC lines were successfully generated.

Human MGE-derived spheroids were differentiated using a modified version of an established protocol (Figure 1A) [46, 55]. The 3-week-old spheroids developed rosette-like structures, which were like the organization of the ventricular zone (VZ) and subventricular zone (SVZ) ($SOX2^+$/$NESTIN^+$, white arrowhead in Figure 1B). The MGE progenitors were indicated by high expression of NKX2.1 (spheroids in Figure 1B and dissociated cells in Figure S2A). At week 8, the MGE lineage spheroids exhibited high expression of the neuronal marker TUJ1, as well as MGE-derived cIN markers SOX6, GAD1, and GABA (spheroids in Figure 1C and dissociated cells in Figure S2B). During the initial phase of methodology development, phenotypes of MGE-derived spheroids at week 16 were characterized in H9-derived spheroids. The results indicated that neural markers such as GFAP (astrocytes), TH (dopaminergic neurons), and OLIG2 (oligodendrocytes) were sparsely expressed, while no IBA1-positive cells (microglia) were detected (Figure S2C). These findings indicate that all the iPSC lines can successfully differentiate along the canonical MGE lineage trajectory.

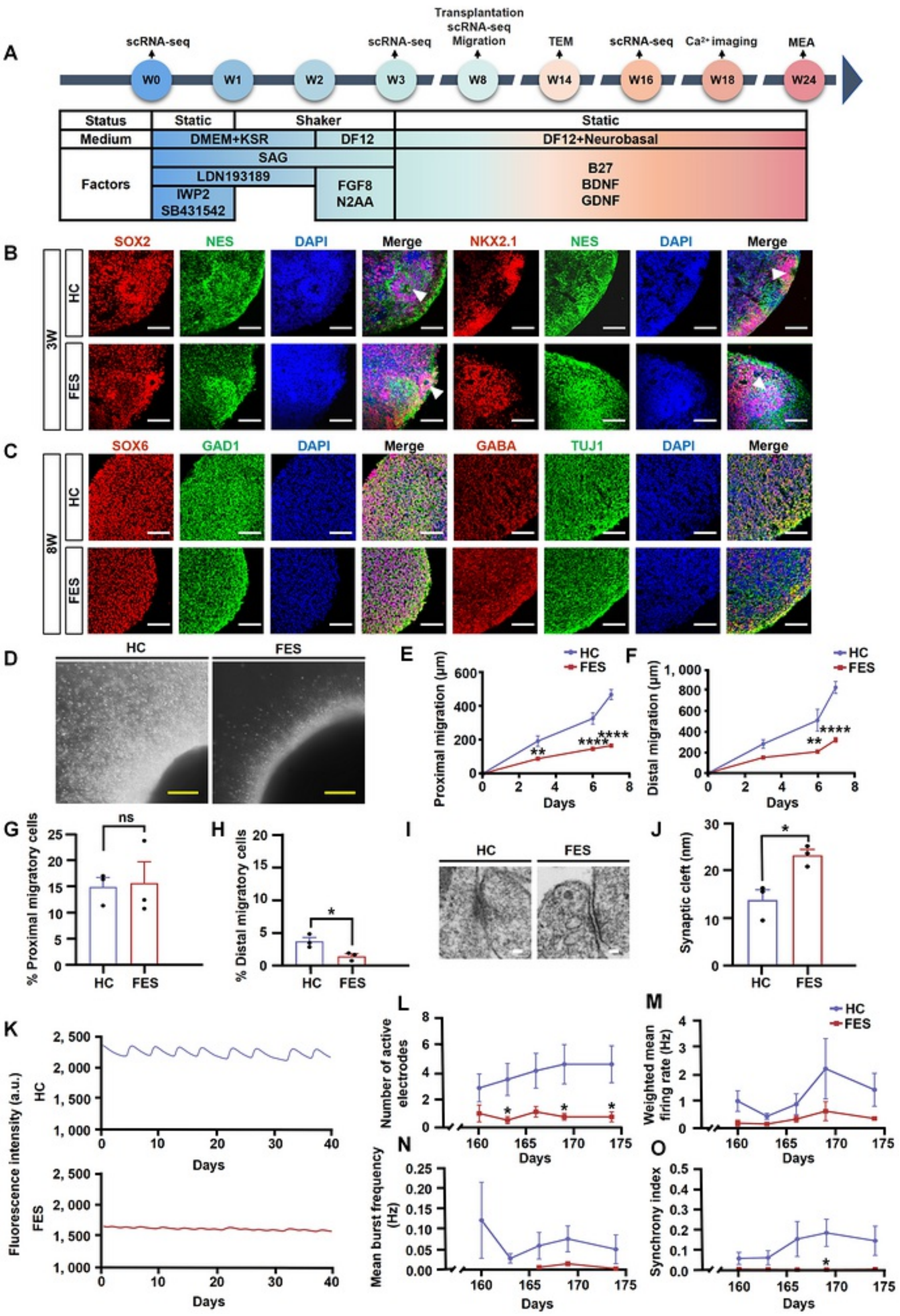

FIGURE 1.

Characterization and Functional Analysis of Human MGE-Derived Spheroids between the HC and FES Groups. (A) Schematic representation of the preparation and analysis of human MGE-derived spheroids. Detailed procedures are provided in the Methods section. (B) Immunostaining of MGE progenitor and neuronal markers in 3-week-old spheroids. White arrowheads in the merged images indicate neural rosettes. White scale bar = 100 μm. (C) Immunostaining of MGE-derived cIN markers in 8-week-old spheroids. White scale bar = 100 μm. (D) Phase-contrast images showing the morphology of migratory cells after 7 days of migration within Matrigel from 8-week-old spheroids. Yellow scale bar = 500 μm. (E-F) Quantification of the proximal (E) and distal (F) migration difference between FES and HC groups in vitro. Data are presented as mean ± SEM. ** $p < 0.01$, and **** $p < 0.0001$ by two-way ANOVA followed by Šídák's post hoc test. HC: n = 6 subjects; FES: n = 4 subjects; for each subject, three spheroids were analyzed. (G,H) Quantitative analysis of the in vivo transplantation experiment reveals differences in proximal (G) and distal (H) migration distances from the graft edge between the HC and FES groups, with data presented as mean ± SEM. * $p < 0.05$ by *t*-test. n = 3 independent subjects per group. HC- and FES-derived MGE spheroids were transplanted into the bilateral cortices of the same recipient mice, with spheroids from each group implanted into one hemisphere. For each subject, spheroids were transplanted into one hemisphere of three recipient mice. (I) TEM images indicate abnormal ultrastructural synaptic abnormalities in the FES group. White scale bar = 100 nm. (J) Quantification of synaptic cleft width in HC and FES groups (n = 3 subjects per group). Data are presented as mean ± SEM. * $p < 0.05$ by *t*-test. (K) Representative traces showing raw calcium fluorescence intensity (a.u.) in MGE-derived spheroids from HC and FES groups. The HC group exhibits regular oscillatory calcium activity, whereas the FES group shows markedly reduced signal amplitude with minimal or absent fluctuations. (L-O) MEA-based electrophysiological analysis of MGE-derived spheroids from HC and FES groups. Defective spontaneous electrophysiology by MEA in the spheroids from the FES group. Data are presented as mean ± SEM. * $p < 0.05$ by two-way ANOVA followed by Šídák's post hoc test. 8 spheroids were analyzed per group.

To further identify the potential functional defects, MGE spheroids were subjected to a series of in vitro and in vivo assessments. The sustained energy-consuming migration is a key feature of the maturation of MGE-derived interneurons and is essential for the proper integration of inhibitory neurons into developing cortical networks [56]. As shown in Figure S2D, migrated cells were classified into two regions: the proximal region (densely populated and adjacent to the spheroid border) and the distal region (sparsely populated and located farther away). In the FES group, the spheroids presented compromised migration over time in both areas in vitro (Figure 1D–F).

Furthermore, 10 weeks after transplanting whole 8-week-old spheroids into the cortex of NOG mice (Figure S2E), the FES group showed no difference in proximal migration (< 400 μm from the graft core) but exhibited impaired distal migration (> 400 μm from the graft core) (Figure 1G,H). The physiological integrity of synaptic structures is essential for efficient neurotransmission and neurotransmitter diffusion across neuronal circuits [57, 58]. Ultrastructural analysis of synapses indicated that neurons within 14-week-old spheroids derived from FES patients displayed expanded synaptic clefts (Figure 1I,J). Such morphological features indicate compromised synaptic integrity and may translate into functional impairments. The quantification of spontaneous calcium dynamics demonstrated that spheroids derived from the FES group exhibited significantly reduced spontaneous calcium transients (both in frequency and amplitude, Figure 1K, Figure S2F, and Video S1). The diminished neuronal excitability and attenuated intracellular calcium signaling (an inherently energy-intensive biological process) collectively indicate severely impaired capacity for generating and propagating spontaneous activity within neuronal networks of FES spheroids. Furthermore, the multielectrode array (MEA) analysis confirmed that MGE-derived spheroids from the FES group exhibited oxygen-dependent electrophysiological deficits, including fewer active electrodes, a tendency toward lower weighted mean firing rate and burst frequency, and decreased network synchrony index (Figure 1L–O; Figure S2G). These findings indicate that despite the preservation of MGE lineage specification, the oxygen-intensive functions of inhibitory neurons are substantially impaired in FES-derived spheroids.

2.2 | Transcriptome-Wide Profiling of MGE-Derived Spheroids

To comprehensively profile transcriptional landscapes in MGE-derived spheroids and investigate cell-intrinsic neurodevelopmental dysregulation underlying SCZ, scRNA-seq was performed at 0, 3, 8, and 16 W (Figure 2A). Totally 116, 483 single cells were harvested and 94, 296 cells passed quality control with a mean of 2, 745 genes and 8, 906 transcripts per cell (Figures S3A-S3D, detailed sample information in Table S6). Dimensionality reduction of cells co-profiled by both 10× Genomics and SCRB-seq platforms demonstrated robust concordance through highly overlapping distributions, indicating strong agreement in transcriptomic capture between technologies (Figure S3E). Furthermore, the reproducibility of the same methods was conducted for two different batches of the same line (FES1) at 3, 8, and 16 W, and the profiles were also highly repetitive (Figure S3F,G). The initial unbiased t-distributed stochastic neighbor embedding (t-SNE) clustering revealed five distinct clusters (Figure 2B), and four of which were annotated by established cell type-specific markers (Figure 2B–I, Figure S4A, and Table S7). A distinct cluster defined by exclusive pluripotency markers (*LIN28A* and *OCT4*) was identified as iPSCs, emerging exclusively at week 0 (Figure 2C,D). NPCs mediating MGE lineage specification were characterized by *VIM* expression (Figure 2E). The cIN cluster was annotated by specifically enriched markers of *GAD1* and *GAD2* (Figure 2F,G). Canonically enriched markers *PDGFRα* and *OLIG2* defined oligodendrocyte progenitor cells (OPCs; Figure 2H,I), which originate as the first MGE-derived wave facilitating cortical cIN migration toward the cortex along appropriate pathways [59]. Cross-validation against a well-established human fetal MGE dataset [52] confirmed concordant cell type-specific expression patterns of the annotated markers (Figure S4B). The iPSCs were exclusively populated at week 0, with NPCs/cINs persisting throughout 3–16 weeks while OPCs emerged significantly only after week 8 (Figure S4C,D), mirroring human MGE development where prolonged neurogenesis precedes gliogenesis [15, 60].

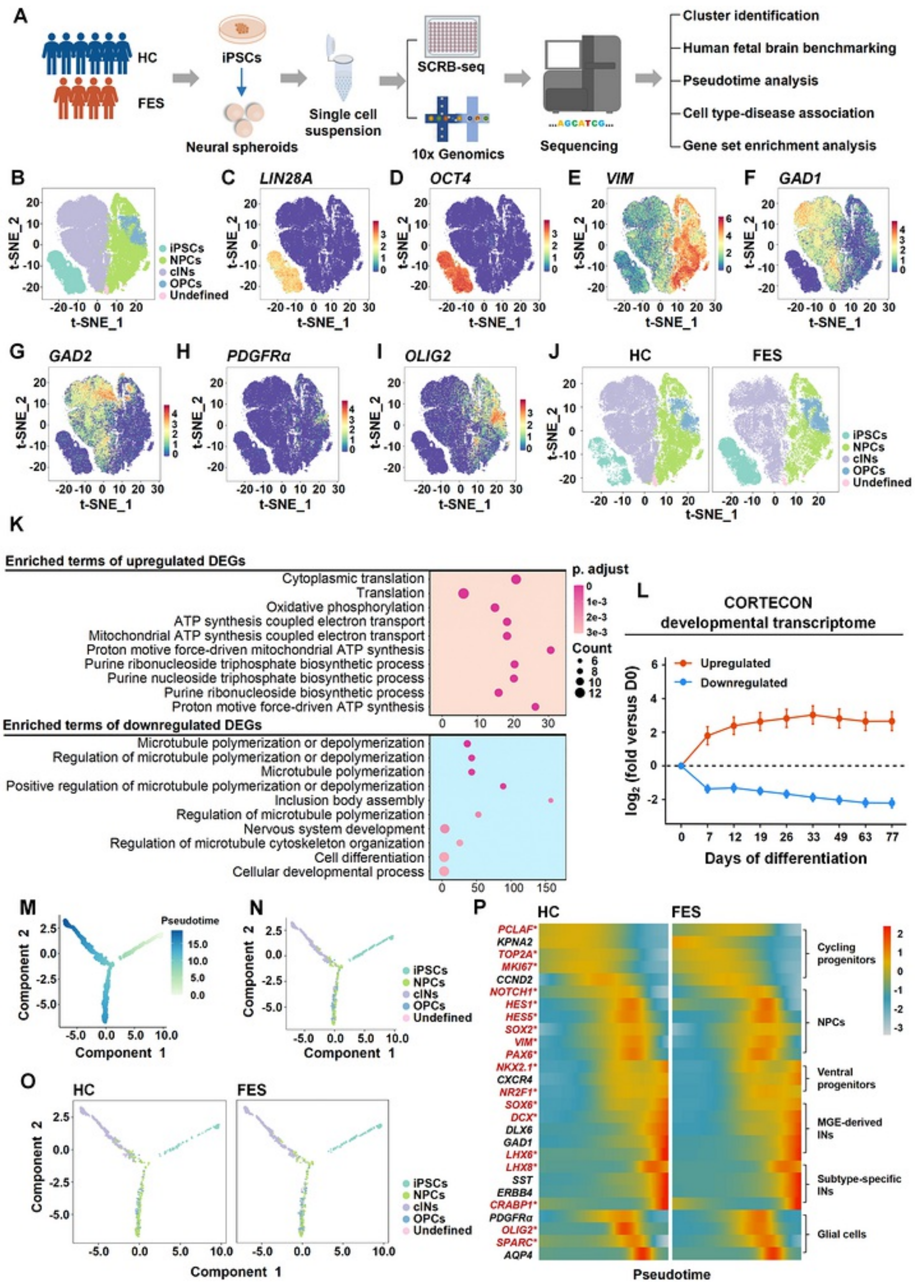

FIGURE 2.

A single-cell resolution transcriptome of MGE-derived spheroids. (A) Workflow and downstream analysis of scRNA-seq transcriptome profiling. The live cells dissociated from spheroids were collected for scRNA-seq, followed by the downstream analysis. (B) The t-SNE visualization shows major cell populations. Cells are colored by cell types. iPSCs, Induced pluripotent stem cells; NPCs, Neural progenitor cells; cINs, Cortical interneurons; OPCs, Oligodendrocyte precursor cells. (C–I) The t-SNE visualization illustrates the expression level of selected marker genes, with red indicating high expression and blue indicating low expression. (J) The t-SNE visualization shows cell types in the HC and FES groups. Cells are colored by cell types. (K) Bubble plots summarizing Gene Ontology-Biological Process (GO-BP) terms significantly enriched among DEGs in total cells from FES versus HC. The upper panel displays GO terms enriched in genes upregulated in FES; the lower panel shows terms enriched in genes downregulated in FES. The x-axis represents the average $\log_2$ fold change of ~~enriched genes within each GO~~{{Tao Li: After verification, the description of the x-axis in Figure 2K has been corrected from "the average log₂ fold change of enriched genes within each GO term" to "the enrichment score" to accurately reflect the metric presented in the figure. This correction does not affect the data or conclusions.}} ~~term~~the enrichment score (FES versus HC). Dot size represents gene count, and color intensity indicates adjusted *p*-value. (L) Line plot showing the temporal expression profile of DEGs (HC vs. FES) in the CORTECON developmental transcriptome (https://cortecon.neuralsci.org), a reference dataset for normal hESC-derived cortical organoid development. Genes upregulated in HC (red line) and downregulated in HC (blue line) follow increasing and decreasing developmental trends, respectively, suggesting greater alignment of HC gene expression with normal cortical organoid maturation. (M) Overview of pseudotime trajectory inferred by Monocle2. Cells are colored by pseudotime. (N) Overview of pseudotime trajectory inferred by Monocle2. Cells are colored by cell types. (O) Pseudotime trajectory in HC and FES groups, respectively. Cells are colored by cell types. (P) Heatmap showing gene expression dynamics of key developmental regulatory genes along pseudotime. The color represents the relative expression level of each gene at each pseudotime point. Gene expression levels were scaled using Monocle2. Differentially expressed genes are highlighted in red. Statistical significance was determined by *t*-test. * $p < 0.05$.

Despite comparable cell composition and spatial distribution in FES vs HC groups by ~~t~~-SNE (Figure 2J), dysregulated genes were identified by differentially expressed genes (DEGs) across non-iPSC clusters (Figure S4E,F, Tables S8–S11) with *SCD*, *DLK1*, and *SPARCL1* emerging as pan-lineage DEGs in NPCs/cINs/OPCs (Figure S4G). These synaptic microenvironment regulators and neural differentiation modulators indicate a global cellular stress response in FES-derived MGE spheroids, broadly perturbing neural development [61–64]. Consistently, Gene Ontology (GO) enrichment analysis of DEGs revealed that downregulated genes were significantly associated with microtubule polymerization and depolymerization and neural differentiation and development, while upregulated genes were enriched in pathways

related to cytoplasmic translation, oxidative phosphorylation, and ATP synthesis (Figure 2K). This gene expression pattern indicates that FES spheroids are under substantial bioenergetic stress, which may drive a compensatory metabolic activation at the cost of impairing proper neural differentiation and maturation. Cross-referencing DEGs with the CORTECON dataset [65] revealed normative neurodevelopmental trajectories: HC-upregulated genes showed progressive elevation during prolonged culture, while FES exhibited divergent temporal regulation patterns, suggesting pathogenic neuronal dysfunction (Figure 2L). Collectively, despite conserved cytoarchitecture, FES patient-derived spheroids exhibit bioenergetic stress-induced metabolic compensation that impairs neural differentiation through divergent temporal genes.

To decode the developmental dynamics of the spheroids, the pseudotime trajectories were constructed to map single-cell developmental routes (Figure 2M,N; Figure S4H,I). Two primary trajectories diverged from iPSCs: a neuronal lineage (NPCs → cINs) and a glial lineage (NPCs → OPCs). Although the global pseudotemporal architecture was conserved between the HC and FES groups (Figure 2O), FES-derived cells showed asynchronous development, characterized by time-dependent dysregulation of neurodevelopmental genes along pseudotime (Figure 2P). GO analysis of pseudotime-stratified DEGs further demonstrated aberrant enrichment in processes related to cell migration, motility, growth, and metal-ion response (Figure S4J and Table S12), recapitulating the functional deficits observed in Figure 1. Together, this pseudotemporal dissection reveals temporally resolved transcriptional dysregulation underlying SCZ pathology.

2.3 | Accelerated Neurodevelopmental Trajectories and Enhanced Hypoxia-Responses in FES-Derived MGE Progenitors

NPCs were further identified using established markers *SOX2* and *NES* (Figure 3A,B). MGE-derived progenitors comprised radial glial cells (RGs) within the VZ and intermediate progenitor cells (IPCs) in the SVZ [14]. Through combinatorial analysis of *NKX2.1* and *ASCL1*, NPCs were stratified into three subtypes (Figure 3C–F, Table S13): (1) *NKX2.1*^{hi}/*ASCL1*^{lo}, ventral RGs; [56] (2) *NKX2.1*^{hi}/*ASCL1*^{hi}, ventral IPCs; [56, 66] and (3) *NKX2.1*^{lo}/*ASCL1*^{lo}, given the elevated *GFAP* expression, classified as astrocyte progenitor-like (ASC-like) cells [67]. The IPC population demonstrated differentiation potential toward cortical interneuron lineages, as indicated by higher expression of DLX family genes compared with RGs, consistent with a more advanced interneuron developmental state that is associated with the onset of migratory programs (Figure 3G–J). Transcriptomic alignment with human ganglionic eminence (GE) reference datasets from gestational weeks (GW) 9–12 revealed conserved molecular signatures between embryonic GE progenitors and our NPC populations (Figure 3K) [51]. Temporal profiling demonstrated RG and IPC emergence at week 3 with progressive upregulation, whereas ASC-like cells appeared later in the differentiation timeline (Figure S5A). Concurrently, NPC subcluster distributions were comparable between groups (Figure S5B). Furthermore, reconstructed NPC developmental trajectories revealed that our spheroids recapitulated key neurodevelopmental dynamics observed in vivo (Figure 3L,M) [56, 66]. Lineage tracing revealed that a subset of cells diverged toward ASC-like cell fate during the RG-to-IPC transition [68]. Pseudotemporal trajectory analysis uncovered disease-associated deviations: density distribution analysis demonstrated a significant rightward shift in FES-derived NPCs (Figure 3N), indicating increased representation in advanced pseudotime states versus HC counterparts. These findings support an accelerated neurodevelopmental trajectory in FES-derived NPCs, potentially reflecting precocious maturation. Gene set enrichment analysis (GSEA) was employed to characterize molecular perturbations in MGE-derived NPCs. NPCs from FES patients exhibited significant enrichment in oxygen-responsive regulatory pathways (Figure 3O, Table S14), suggesting pathological oxidative stress that may compromise neuronal survival, proliferation, and differentiation [69]. Among 22 previously reported oxidative stress-associated genes linked to 108 SCZ-risk loci, [70, 71] 15 were significantly dysregulated in FES-derived NPCs compared to healthy controls (Figure 3P), suggesting early disruptions of redox homeostasis during neurodevelopment. Collectively, FES-derived NPCs exhibited accelerated neurodevelopmental progression concurrent with oxidative stress-associated transcriptional remodeling. These findings support a dual dysregulation of developmental timing and metabolic homeostasis during early cIN differentiation from MGE progenitors, potentially establishing cellular vulnerabilities that prime SCZ risk.

{{Tao Li: The merged image in Figure 3K was incorrect due to an inadvertent file selection error. After verification of the original data, the correct merged image has been replaced. This correction does not affect the quantitative analysis or conclusions of the study.}}

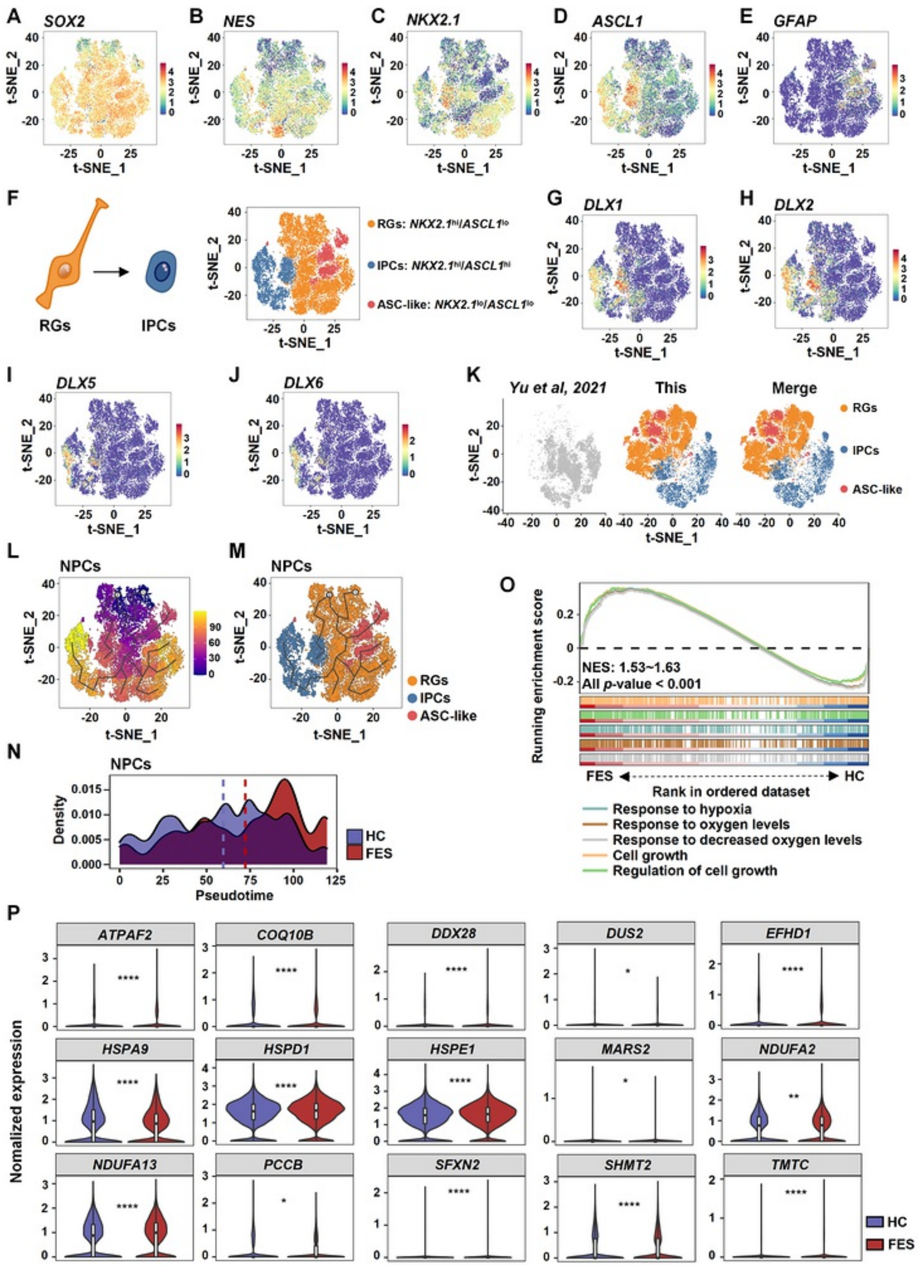

FIGURE 3.
Accelerated neurodevelopmental trajectories and enhanced hypoxia response in FES-derived MGE progenitors revealed by transcriptomic diversity. (A–E) The t-SNE visualization shows the expression level of neural progenitor markers in NPCs, with red indicating high expression and blue indicating low expression. (F) Schematic diagram and t-SNE visualization showing the differentiation potential and subtypes of NPCs. *NKX2.1*^{hi}/*ASCL1*^{lo}: RGs; *NKX2.1*^{hi}/*ASCL1*^{hi}: IPCs; and *NKX2.1*^{lo}/*ASCL1*^{lo}: ASC-like cells. IPCs, intermediate progenitor cells; RGs, radial glial cells. (G–J) The t-SNE visualization shows the expression patterns of migratory markers in NPCs, with red indicating high expression and blue indicating low expression. (K) The t-SNE plot shows the integrated analysis of NPC datasets from this study and human embryonic GE progenitors from Yu et al. (2021), (51) with cells colored by subtype and data source. (L,M) Reconstructed single-cell trajectories of NPCs colored by pseudotime (L) or cell subtypes (M). (L) Line plots showing the inferred developmental trajectories of NPCs reconstructed by trajectory inference algorithms. Cells are colored by pseudotime values, indicating their relative positions along a continuous developmental path. Color gradients from purple to yellow represent progression from early to late pseudotime. (M) The same trajectories are visualized with cells colored by their predicted subtypes. Black lines indicate the main developmental branches, and white circles denote starting points identified by the trajectory analysis. These reconstructions illustrate subtype divergence along the developmental trajectory. (N) Density plots showing cell distribution of NPCs along the pseudotime in HC and FES groups. The x-axis represents the pseudotime trajectory, while the y-axis reflects the density of cells at each pseudotime point. Density curves represent the relative frequency of cells at different pseudotimes. Dashed vertical lines indicate the mean pseudotime value for each group. (O) Enriched biological processes in NPCs derived from the FES group, identified by GSEA using genes ranked by fold change. Significance was determined with an adjusted *p*-value < 0.05 (Benjamini-Hochberg correction). NES: normalized enrichment score. (P) Violin plots showing 15 differentially expressed oxidative stress–related genes in NPCs derived from the FES group. * $p < 0.05$, ** $p < 0.01$ and **** $p < 0.0001$ by *t*-test. The oxidative stress-related genes are derived from the 108 genome-wide significant schizophrenia risk loci reported in the landmark GWAS by the Psychiatric Genomics Consortium [70].

2.4 | Persistent Developmental Timing Shift and Redox Risk Gene Dysregulation in cINs Derived From FES

MGE-derived precursors undergo progressive differentiation during migratory phases, ultimately maturing into cortical SST$^+$ and PV$^+$ interneuron subtypes [56, 72]. Single-cell disease relevance score (scDRS) analysis identified cINs as the cell population most robustly associated with SCZ, exhibiting significantly higher disease-relevance scores than neural progenitors or glial lineages (Figure 4A). These

findings align with the established roles of these interneurons in maintaining excitation-inhibition balance and circuit functionality, both of which are core pathophysiological features of SCZ [73–75]. The cIN population predominantly comprises young, migratory-competent cells (Figure 4B; Figure S6A,B). The *LHX6*, a transcription factor critical for human MGE-derived interneuron specification and migration through NKX2.1 suppression was then assessed [76]. Stratification by *LHX6* expression delineated two cIN subtypes: *LHX6*^{hi} (more mature) and *LHX6*^{lo} (less mature), distinguished by differential expression of cINs markers (e.g., *SOX6* and *ARX*) (Figure 4C,D, Figure S6C–F; Table S15) [56, 77, 78]. Additionally, some markers of cIN subtypes also emerged, including *SST*, *ERBB4*/*ETV1*/*CRABP1* (*PV* interneuron precursors) [51, 52], as well as *LHX8*, a key transcription factor regulating subpallial cholinergic neuron development (Figure 4E,G, Figure S6G,H) [79]. Notably, *LHX6*/*LHX8* co-expressing cells representing subpallial cholinergic neurons (Figure 4C,G) [52, 56, 80]. Transcriptomic mapping against human fetal MGE neurons references (GW9-18) demonstrated recapitulation of early embryonic developmental processes in our induced cINs (Figure 4H,I) [52]. Notably, cINs from the FES group appeared to correspond to a relatively later developmental stage of human MGE neurons (Figure S6I). The downregulation of *MKI67* indicated that more cINs have exited the cell cycle and initiated migration toward the cortex (Figure S6J). In parallel, persistent *LHX6*^{hi} and *LHX6*^{lo} subpopulations suggested continuous interneuron production and maturation (Figure S6L), implying embryonic-stage developmental disruptions may confer SCZ risk. Consistently, pseudotime trajectory analysis revealed *LHX6* upregulation in late-stage cINs (Figure 4J,K), and FES-derived cINs exhibited advanced developmental progression recapitulating NPC acceleration patterns (Figure 4L).

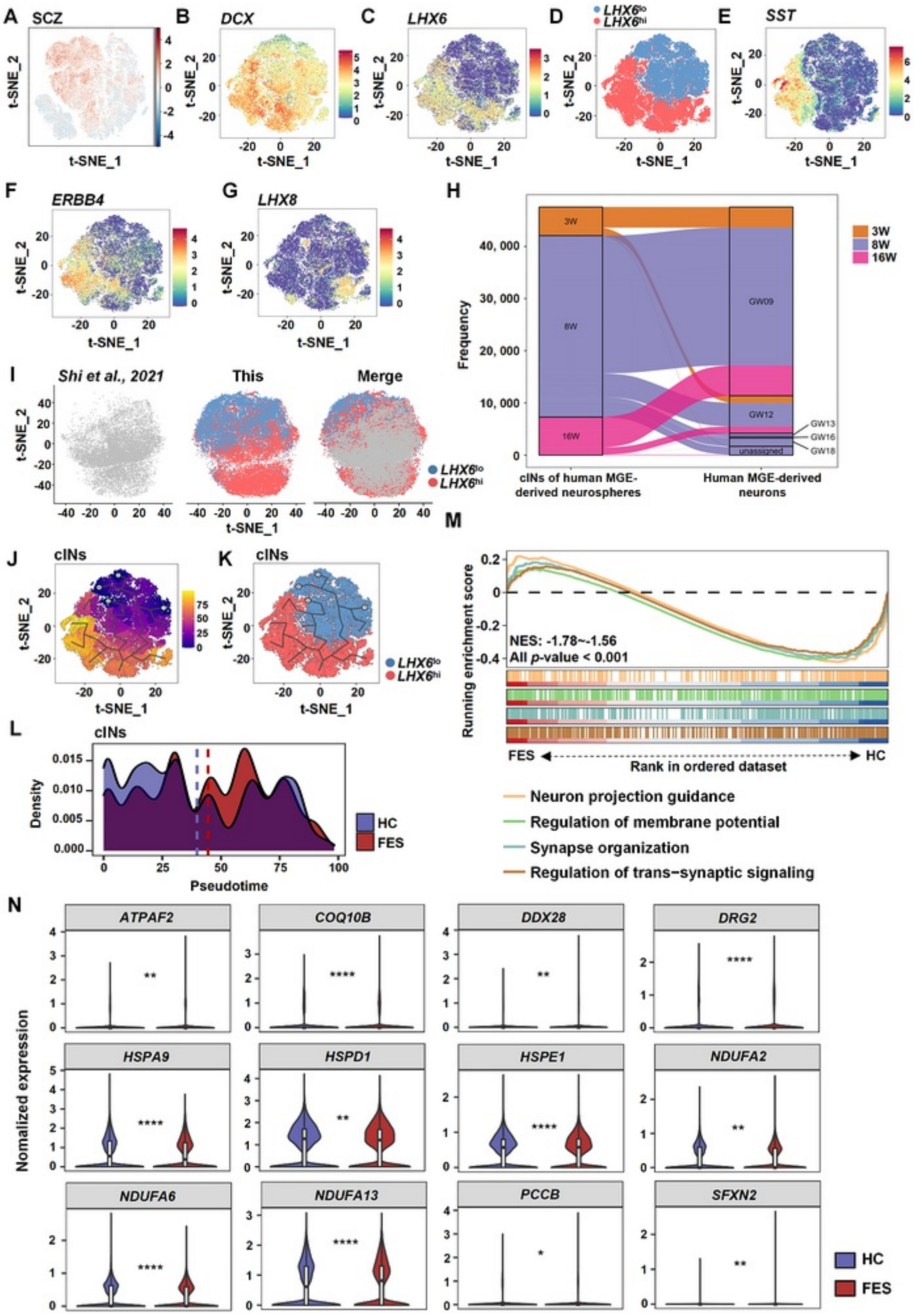

FIGURE 4.
Transcriptomic features of cINs and persistent developmental acceleration and redox gene dysregulation in FES-derived cINs. (A) t-SNE visualization showing single-cell disease relevance scores (scDRS), with high scores in red and low scores in blue. (B) The t-SNE visualization shows that the marker gene *DCX*, a marker for young neurons, is uniformly highly expressed in cINs, with red indicating high expression and blue indicating low expression. C) The t-SNE visualization shows the expression of *LHX6* in cINs, with red indicating high expression and blue indicating low expression. (D) The t-SNE visualization shows the subtypes of cINs based on *LHX6* expression. *LHX6*^{lo}: cells with low levels of *LHX6* expression; *LHX6*^{hi}: cells with high levels of *LHX6* expression. (E–G) The t-SNE visualization shows the expression patterns of cIN markers of *SST*, *ERBB4*, and *LHX8*, with red indicating high expression and blue indicating low expression. (H) Sankey diagram illustrating the temporal correspondence between cINs and human embryonic MGE interneurons [52]. (I) The t-SNE visualization shows the integration of cINs and

human embryonic MGE-derived neurons, colored by subtypes and data sources [52]. (J,K) Reconstructed single-cell trajectories of cINs colored by pseudotime (J) or cell subtypes (K). (J) Line plots showing the inferred developmental trajectories of cINs reconstructed by trajectory inference algorithms. Cells are colored by pseudotime values, indicating their relative positions along a continuous developmental path. Color gradients from purple to yellow represent progression from early to late pseudotime. (K) The same trajectories are visualized with cells colored by their predicted subtypes. Black lines indicate the main developmental branches, and white circles denote starting points identified by the trajectory analysis. These reconstructions illustrate subtype divergence along the developmental trajectory. (L) Density plots showing cell distribution of cINs along the pseudotime in FES and HC groups. The x-axis represents the pseudotime trajectory, while the y-axis reflects the density of cells at each pseudotime point. Density curves represent the relative frequency of cells at different pseudotimes. Dashed vertical lines indicate the mean pseudotime value for each group. (M) Negative{{Tao Li: The enrichment direction in Figure 4M was incorrectly described and has been corrected. This change does not affect the results or conclusion. }}ly ~~E~~enriched biological processes in cINs derived from the FES group, identified by GSEA using genes ranked by fold change. Significance was determined with an adjusted p-value < 0.05 (Benjamini–Hochberg correction). NES: normalized enrichment score. (N) Violin plots showing the expression differences of 12 oxidative stress-related schizophrenia risk genes in cINs derived from the FES group. (70) * $p < 0.05$, ** $p < 0.001$ and **** $p < 0.0001$ by t-test.

GSEA of week 16 cINs identified FES-associated downregulation in functional pathways critical for neuronal connectivity: axon guidance, synaptic organization, membrane potential regulation, and trans-synaptic signaling (Figure 4M, Table S16). This accelerated maturation compromises functional specialization, particularly at the expense of synaptic development. Among 22 redox-linked SCZ risk genes, 12 exhibited significant dysregulat~~ion~~ed in FES patients-derived cINs (Figure 4N), underscoring oxidative stress as a persistent pathomechanism. This sustained redox imbalance may impair energy-demanding neuronal processes, including electrical activity, migration, and synaptic transmission, thereby compromising the functional integration of cINs into cortical circuits. The convergence of redox imbalance and temporal compression during neurodevelopment suggests redox-mediated dysregulation may potentiate SCZ susceptibility through simultaneous metabolic and maturational metabolic perturbations.

2.5 | Accelerated Cell Cycle and Defective Interneuron Development Induced by Gestational Hypoxia

Persistent oxidative stress signatures across NPC and cIN stages in SCZ patient-derived MGE neurospheroids motivated an interrogation of whether gestational hypoxia (an established oxidative stress trigger) perturbs the development of MGE-derived cINs in vivo. A gestational hypoxia paradigm was established by exposing pregnant mice to 10% oxygen for 48 h beginning at embryonic day 9.5 (E9.5), while control pregnant mice were maintained under normoxic ambient air conditions (~21% O_2). The induction efficacy was confirmed by elevated HIF-1α immunoreactivity at E11.5 (Figure 5A) [81]. Fetal resorption and mortality remained indistinguishable between normoxic and hypoxic litters at E12.5, E15.5, and E18.5 (Figure S7A), and hypoxic embryos maintained body widths comparable to controls throughout gestation (Figure S7B,C). Transient decrements in body length and weight at E12.5 and E15.5 normalized by E18.5 (Figures S7B and S7D,E). Thickness of VZ, gauged by OLIG2/DCX staining, showed no deviation at E12.5 and E15.5 (Figure S7F,G). Proliferation of the MGE region was subsequently evaluated via EdU–BrdU dual-pulse labeling (Figure 5B). BrdU incorporation within 1 h before perfusion revealed a significant reduction in S-phase entry in the VZ and SVZ of hypoxic embryos at E12.5 (Figure 5C–E). By E15.5, both EdU (3-h window) and BrdU (1-h window) labeling showed pronounced reduction of S-phase entry across proliferative zones (Figure 5F–H), indicating pervasive progenitor proliferation impairment. The temporal lag prompted further cell-cycle scrutiny. The elevated $EdU^{+}BrdU^{-}$ cells (post-S-phase exiting cells) in hypoxia embryos at E12.5 and E15.5 (Figure 5I–K) pointed to accelerated S-phase completion and cell cycle progression. Concordantly, diminished P27 expression in the ventral forebrain at E12.5 and E15.5 corroborated hypoxia-driven cell-cycle compression (Figure S7H,I) [82, 83]. Equivalent apoptosis frequencies (TUNEL assay) ruled out cell death as a confound (Figure S7J,K). To further determine whether gestational hypoxia altered postnatal maternal care, we assessed maternal behaviors during the early lactation period. Nest-building performance at P1 was comparable between normoxic and hypoxic dams (Figure S8A,B). In addition, the number of pups per litter, pup survival rate at P7, milk band score, latency to retrieve three pups, and latency to retrieve the first pup at P1 showed no significant differences between groups (Figure S8C–H). These findings suggest that gestational hypoxia did not detectably impair maternal care or early pup viability, reducing the possibility that the subsequent offspring behavioral phenotypes were primarily attributable to altered maternal care during lactation.

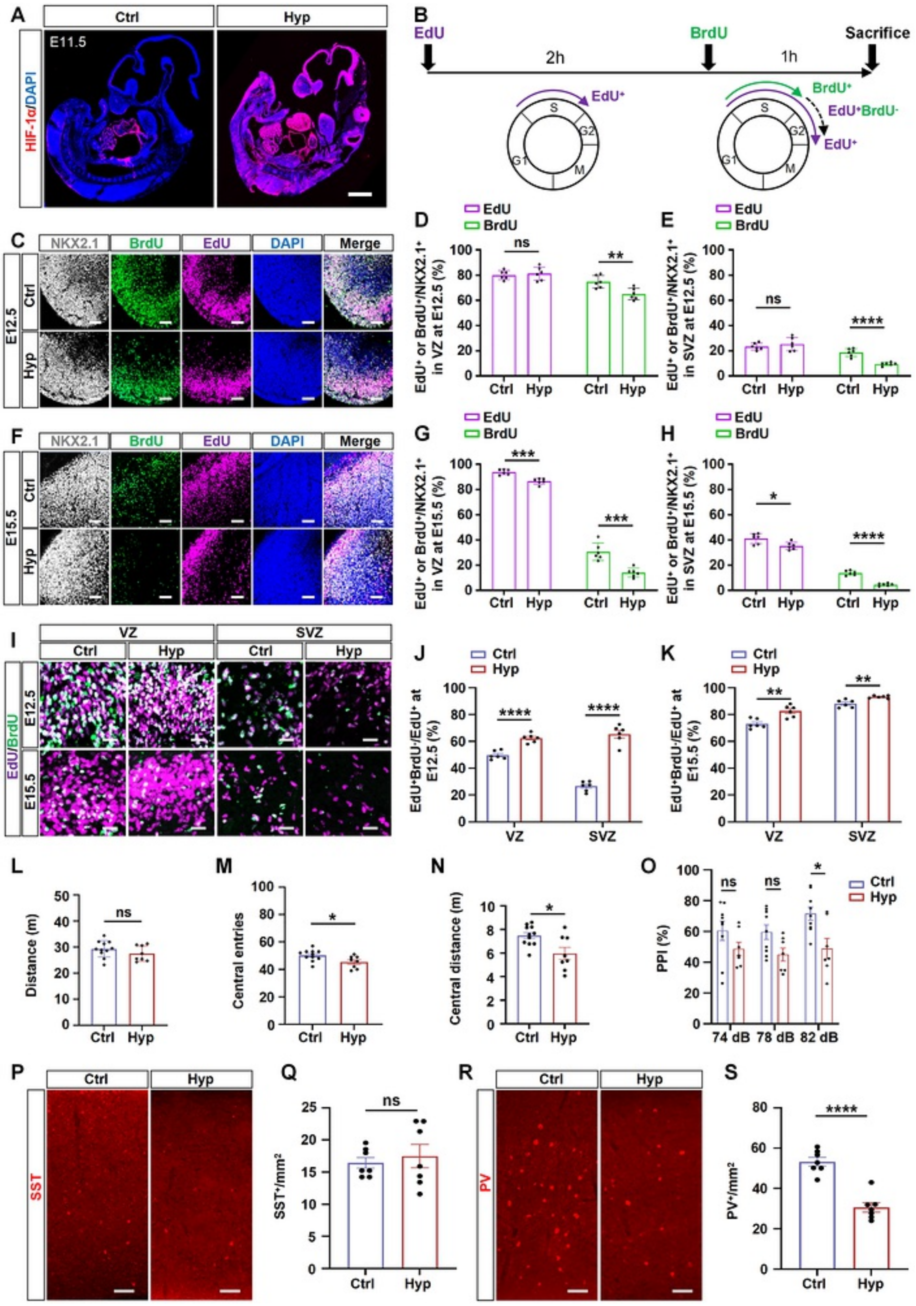

FIGURE 5.

Accelerated cell cycle and impaired interneuron development in mice induced by gestational Hypoxia. (A) Immunofluorescence staining of HIF-1α in sagittal sections of E11.5 mouse embryos under normoxia and hypoxia. White scale bar = 1 mm. (B) Schematic diagram of the EdU/BrdU pulse-chase labeling strategy and resultant cell cycle labeling patterns. The illustration depicts how EdU^+, $BrdU^+$, and $EdU^+BrdU^−$ cells represent distinct stages of S-phase progression within the cell cycle. (C) Representative immunofluorescence images of the MGE at E12.5 under normoxia and hypoxia with the EdU/BrdU pulse-chase labeling strategy. White scale bar = 100 μm. (D,E) Quantification of EdU^+ and $BrdU^+$ cells within $NKX2.1^+$ progenitors of the MGE at E12.5 (VZ, D; SVZ, E). n = 6 embryos from independent litters per group. (F) Representative immunofluorescence images of the MGE at E15.5. White scale bar = 100 μm. (G,H) Quantification of EdU^+ and $BrdU^+$ cells within $NKX2.1^+$ progenitors of the MGE at E15.5 (VZ, G; SVZ, H). n = 6 embryos from independent litters per group. (I) Representative immunofluorescence images of the MGE at E12.5 and E15.5. White scale bar = 25 μm. (J,K) Quantification of putative MGE cell-cycle exit at E12.5 (J) and E15.5 (K) by computing the ratio of $EdU^+BrdU^−$ cells to total EdU^+ cells. n = 6 embryos from independent litters per group. (L–N) Gestational hypoxia exposure effects on the open field behavior of adult offspring, including total distance traveled (L), Number of entries into the center zone (M), and Distance traveled within the center zone (N). n = 11 mice in the control group and n = 8 mice in the hypoxia group. (O) Gestational hypoxia exposure effects on the PPI of adult offspring. n = 9 mice in the control group and n = 7 mice in the hypoxia group. (P,Q) Immunofluorescence staining (P) and quantification (Q) of SST^+ interneurons in the frontal cortex of adult mice under normoxia and hypoxia. White scale bar = 100 μm. n=7. (R,S) Immunofluorescence staining (R) and quantification (S) of PV^+ interneurons in the frontal cortex of adult mice under normoxia and hypoxia. White scale bar = 100 μm. n = 7. Detailed statistical results for these analyses are provided in Table S18.

Adult offspring exposed to gestational hypoxia displayed SCZ-like behavioral profile: intact locomotion but diminished center exploration in the open field test (Figure 5L–N, Figure S7L) and impaired prepulse inhibition (PPI, Figure 5O and Figure S7M). In accord with SCZ-associated PV^+ interneuron pathology [84–86], SST^+ interneuron density was unchanged (Figure 5P,Q), whereas ~~pre~~frontal cortical PV^+ populations were significantly reduced (Figure 5R,S). This selective PV^+ interneuron vulnerability aligns with the heightened oxidative sensitivity conferred by the elevated metabolic demands of PV^+ interneurons and with their established role in SCZ pathophysiology, [87–89] thereby recapitulating core disease features. Crucially, the shortened cell cycle observed in gestational MGE progenitors parallels the accelerated neurodevelopmental trajectories evident both in the hypoxic mouse model and FES patient-derived MGE spheroids, implying the

convergence on oxidative stress-driven mechanisms of developmental dysregulation that ultimately contribute to SCZ-like phenotypes.

2.6 | N-Acetylcysteine-Mediated Rescue of Gestational Hypoxia-Induced Interneuron Development Deficits and Offspring SCZ-Like Behaviors

To explore the pharmacological rescue potential for oxidative stress-related deficits, hypoxia-exposed pregnant mice were administered NAC, a potent antioxidant known to mitigate oxidative damage [19]. NAC was delivered at two dosages: 100 mg/kg (low dose) and 200 mg/kg (high dose). Strikingly, EdU and BrdU incorporation in the VZ and SVZ of the fetal MGE at E15.5 was nearly restored to normoxic levels following NAC administration (Figure 6A–E). Subsequent quantification demonstrated that high-dose NAC normalized the elevated S-phase exit ratio specifically within the VZ, while both doses restored SVZ ratios to baseline (Figure 6F–H), collectively indicating full normalization of cell-cycle dynamics. With proliferative rescue confirmed, adult offspring underwent behavioral profiling. In the open field test, hypoxia-induced deficits in center exploration were reversed comparably by both NAC doses (Figure 6I–K and Figure S7N). Most notably, PPI assays revealed that a low dose of NAC achieved complete restoration of sensorimotor gating impairments in hypoxia-exposed offspring (Figure 6L and Figure S7O). To assess whether interneuron development was concomitantly restored, PV$^+$ interneurons were quantified in the ~~medial pre~~frontal cortex of adult offspring. Both doses of NAC treatments fully restored the PV$^+$ interneuron density to normoxic control levels (Figure 6M,N), confirming effective rescue of MGE-derived interneuron specification. These findings collectively demonstrate that maternal NAC intervention sufficiently reverses embryonic hypoxia-induced disruptions in progenitor proliferation, interneuron development, and SCZ-like behaviors. Collectively, our results position gestational hypoxia as a direct driver of both proliferative impairment and SCZ-like behaviors. The consistent rescue across cellular and functional domains supports NAC-mediated hypoxia-initiated redox-targeting strategies as a promising therapeutic avenue for mitigating interneuron-related neurodevelopmental deficits.

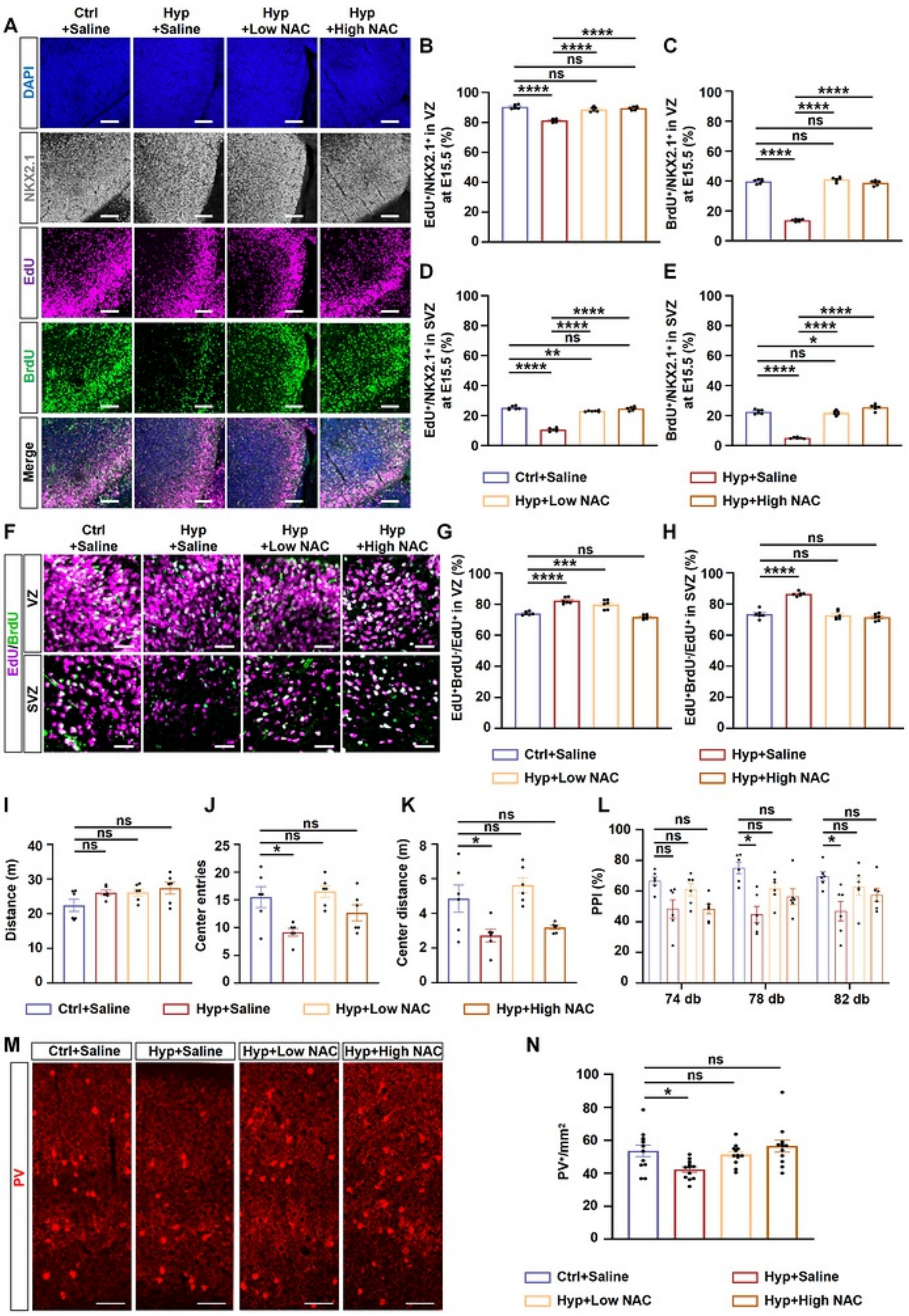

FIGURE 6.

N-acetylcysteine-mediated rescue reverses offspring deficits induced by gestational hypoxia. (A) Representative immunofluorescence images of the MGE at E15.5 demonstrating the rescue of proliferation deficits by low- and high-dose NAC treatment following gestational hypoxia. White scale bar = 100 μm. (B–E) Quantification of EdU$^+$ or BrdU$^+$ cells within NKX2.1$^+$ progenitors of the MGE at E15.5 in the VZ and SVZ. n = 6 embryos from independent litters per group. (F) Representative immunofluorescence images of the MGE at E15.5 demonstrating the rescue of proliferation deficits by low- and high-dose NAC treatment following gestational hypoxia. White scale bar = 25 μm. (G,H) Quantification of putative MGE cell-cycle exit at E15.5 in the VZ (G) and SVZ (H) by computing the ratio of EdU$^+$BrdU$^-$ cells to total EdU$^+$ cells. n = 6 embryos from independent litters per group. (I–K) Effects of maternal NAC treatment on the open field behavior of adult offspring, including total distance traveled (I), Number of entries into the center zone (J), and Distance traveled within the center zone (K). n = 6. (L) Effects of maternal NAC treatment on the PPI of adult offspring. n = 6. (M,N) Immunofluorescence staining (M) and quantification (N) of PV$^+$ interneurons in the frontal cortex of adult offspring following ~~or~~ maternal NAC treatment. n = 12. White scale bar = 100 μm. Detailed statistical results for these analyses are provided in Table S18.

3 | Discussion

Elucidating the neurodevelopmental origins of SCZ remains a pivotal yet unresolved challenge in psychiatric neuroscience. To capture the first molecular and cellular missteps that launch SCZ, high-resolution single-cell transcriptomes of patient-derived medial ganglionic eminence spheroids were paired with an embryonic hypoxia mouse model. This convergence forges a mechanistic bridge that interrogates and validates events across genetically faithful human brain tissue and a precisely controlled prenatal insult. This integration reveals convergent impairments in developmental timing, oxidative stress homeostasis, and neuronal function, thereby establishing these pathways as core mechanistic components underlying SCZ's dysregulated neurodevelopmental trajectory. A notable strength of this study lies in its rigorous sample selection and modeling strategy. Exclusive enrollment of medication-naïve Han Chinese adolescents with first-episode SCZ mitigates potential confounders including chronic medication exposure, accumulated epigenetic drift, and population stratification. All iPSC lines were generated via integration-free episomal vectors to avoid artificial genomic alterations. This careful design strategy reduces biological noise and improves the interpretability of patient-specific phenotypes given the heterogeneous nature of SCZ risk architecture.

Importantly, our differentiation system robustly recapitulated the cellular complexity and temporal dynamics of human MGE development. The scRNA-seq combined with trajectory inference delineated progressive neurogenesis from iPSCs through NPCs to cINs, faithfully modeling the subpallial lineage hierarchy (Figure 2B,N). Human embryonic studies indicate that the MGE region comprises nest-like shells surrounding densely packed aggregates of highly *VIM*-expressing NPCs [15, 90]. These NPCs progressively mature into interneurons persisting the nest-like architecture until GW39, revealing continuous NPC and interneuron generation. Consequently, *VIM* served as an NPC identification marker. Although current MGE-derived spheroid models lack this nest-like organization, [46, 91, 92] parallel generation of NPCs and cINs was nonetheless observed throughout the differentiation period (Figure S4C). Furthermore, our MGE-derived spheroids successfully model the development of two principal human MGE progenitor subtypes: RGs (*NKX2.1*^{hi}/*ASCL1*^{lo}) and IPCs (*NKX2*.1^{hi}/*ASCL1*^{hi}) (Figure 3F) [52]. This classification is in line with murine models where *ASCL1* is a key regulator driving the RG-to-IPC transition during subpallial neurogenesis [93]. A distinct subtype exhibiting high *GFAP* expression alongside low levels of *NKX2.1* and *ASCL1* was identified, which was tentatively classified as an ASC-like progenitor cell (Figure 3E,F). This cell type is theorized to originate from the subpallium, an area whose developmental contributions remain incompletely characterized [94]. This modeling fidelity establishes MGE spheroids as a physiologically relevant platform for probing early neurodevelopmental disruptions in SCZ.

LHX6 was specifically emphasized given its pivotal role in the development and functional regulation of cINs. As a LIM-homeodomain transcription factor predominantly expressed in the MGE and directly regulated by *NKX2.1*, [95, 96] *LHX6* orchestrates interneuron migration and cortical distribution. Our results revealed that *LHX6*^{hi} subpopulations correlate with advanced maturation markers (*SOX6*, *ARX*; Figure 4C, Figure S6C–F), essential for specification into SST$^+$ and PV$^+$ GABAergic subtypes [78, 97]. Upon commitment to the cIN lineage, *MKI67* expression cessation (Figure S6J) signifies cell cycle exit and fate specification initiation. The spheroids generated in this study displayed predominant SST$^+$ interneurons (Figure 4E) but lacked detectable PV$^+$ interneurons (Figure S6K). This finding aligns with the established developmental sequence of cINs, characterized by the initial emergence of SST-expressing neurons followed later by PV-expressing neurons [49, 56]. This pattern indicates that the spheroids model a relatively immature developmental stage analogous to human cIN development at approximately GW9-12 (Figure 4H). Although PV$^+$ interneurons (strongly implicated in SCZ pathology) [98–100] typically mature postnatally, their precursors expressing elevated *ErbB4*/*ETV1*/*CRABP1* levels were already detectable within spheroids (Figure 4F, Figure S6G,H). It is therefore suggested that developmental abnormalities occurring during early prenatal stages may disrupt the subsequent postnatal trajectory and functionality of PV$^+$ interneurons. One of the central findings of this study is the accelerated neurodevelopment observed in MGE spheroids derived from SCZ patients. Pseudotime analysis revealed accelerated developmental trajectories of both NPCs and cINs (Figures 3N and 4L), indicating faster progression along the differentiation pathway. This phenomenon was consistently demonstrated in our FES-derived MGE spheroid model and corroborated recent reports of accelerated striatal inhibitory progenitor development in ventral forebrain organoids derived from SCZ patients [101]. Concurrent stage-spanning oxidative stress signatures accompanied the temporal dysregulation, evidenced by heightened hypoxic responses in NPCs (Figure 3O,P) and persistent aberrant expression of oxidative stress-associated risk genes in cINs (Figure 4N). Meanwhile, interneurons derived from FES patients exhibited pronounced impairments in several energy-intensive functions. Spheroid migration of the FES group presented compromised migration in both proximal and distal regions, with pronounced deficits distally (Figure 1D–H). The impairment of cellular guidance mechanisms leads to defective cortical positioning, and this energy-intensive migratory process has been a well-documented defect

underlying SCZ [102–104]. Transmission electron microscopy analysis of 14-week-old spheroids revealed shrunken synaptic structures with expanded clefts in the FES group (Figure 1I,J). The altered cleft width is likely to compromise the energy-intensive trans-synaptic trafficking of neurotransmitters and associated cargos, further exacerbating synaptic inefficiency [105]. Calcium imaging and MEA recordings further demonstrated significant electrophysiological deficits (Figure 1K–O), consistent with the high energy demands of neuronal activity. These results collectively suggest that early redox dysregulation initiates cascading developmental anomalies, culminating in aberrant formation and function of inhibitory circuitry central to SCZ pathology.

The stage-spanning hypoxic signatures in NPCs and cINs, coupled with selective vulnerability of energy-intensive processes, suggest that early redox imbalance precipitates premature lineage commitment and progenitor progression along the differentiation trajectory. Gestational hypoxia, an established risk factor for SCZ and a potent oxidative stress driver, [106–108] simultaneously suppresses NPC proliferation, accelerates cell cycle progression, and imprints adult offspring with SCZ-like behavioral phenotypes. These findings were confirmed by cortical developmental abnormalities in DISC1 knockdown mice [109]. Hypoxia-induced acceleration of S-phase exit and cell cycle progression (Figure 5B,I–K), leading to early neurodevelopmental asynchrony that promotes NPC exit from the proliferative state. Risk-associated loci that are up-regulated during gestation are disproportionately concentrated in cell-cycle–related genes, pointing to cell-cycle dysregulation as a central mechanistic node in SCZ [110]. Reduced expression of histone methyltransferase PRMT7, an enzyme critical for maintaining NPC proliferation, accelerates cellular differentiation and thereby genetically links SCZ risk to dysregulated cell-cycle control [111]. Complementarily, primary olfactory neurosphere cultures from patients display shortened cell cycles relative to controls [112]. Cell-cycle aberrations are further causally linked to perturb interneuron fate and maturation. Hypoxic mice exhibited reduced PV^+ interneurons, though this phenotype cannot yet be verified in MGE models before PV^+ interneuron generation. In summary, these findings highlight that gestational hypoxia, through convergent oxidative stress and cell-cycle dysregulation, drives premature NPC differentiation and developmental asynchrony, thereby laying a neurodevelopmental foundation for SCZ vulnerability. However, an important limitation of our study is that we did not directly manipulate redox state in the MGE spheroid system to establish causality between oxidative stress and altered developmental trajectories. In this study, redox-related abnormalities were already evident at the NPC stage and coincided with altered pseudotemporal progression and dysregulation of developmental regulatory genes. Matched single-cell comparisons reduced confounding from differences in cell-type composition, while the gestational hypoxia mouse model further provided in vivo causal support. Importantly, maternal NAC supplementation restored progenitor proliferation and normalized cell-cycle kinetics, suggesting that redox imbalance may contribute to the regulation of early neurodevelopmental trajectories. Nevertheless, redox-targeted therapeutic strategies should still be considered with caution.

NAC, a thiol-containing antioxidant already approved for acetaminophen overdose and chronic pulmonary disease, [113] exerts pleiotropic actions that converge on glutathione resynthesis, direct scavenging of reactive oxygen species, and restoration of intracellular redox balance, mechanisms increasingly recognized as central to the pathophysiology of neuropsychiatric disorders. SCZ is characterized by an established deficit in cortical glutathione and heightened oxidative burden,[27] particularly during prenatal neurodevelopment. By exploiting this mechanistic link, maternal NAC supplementation rescued the proliferation of MGE progenitors, normalized their cell-cycle kinetics (Figure 6A–H), and thereby augmented the postnatal complement of parvalbumin-expressing interneurons in the cortex (Figures 6M,N). These cellular corrections translated into enduring behavioral improvements, manifested as reduced locomotor hyperactivity and restored sensory gating, both endophenotypes of SCZ (Figure 6I–L). The results collectively establish that redox-directed intervention during gestation can prevent the emergence of interneuronopathy associated with SCZ risk, define a critical window for prophylaxis, and reposition NAC from a generic antioxidant to a disease-modifying agent for high-risk populations. From a translational perspective, the high-risk populations referred to here are not all pregnancies, but those exposed to hypoxia-related prenatal or perinatal adversities, particularly when combined with schizophrenia-associated genetic susceptibility or other biological vulnerabilities. Such contexts may include preeclampsia, placental insufficiency, fetal growth restriction, low birth weight, preterm birth, perinatal asphyxia, and other obstetric complications that increase fetal hypoxic or oxidative burden [108]. Given the heterogeneity of schizophrenia, gestational hypoxia should not be viewed as an independent etiological subtype, but as a clinically relevant prenatal risk pathway that may interact with genetic, inflammatory, placental, and redox-related mechanisms [107, 114, 115]. Similarly, NAC's protective effects are unlikely to be specific to hypoxia, as NAC targets glutathione metabolism, oxidative stress, inflammation, glutamatergic signaling, and mitochondrial function [116]. However, our findings do not support routine NAC use during pregnancy; its preventive application requires further validation in defined high-risk populations.

MGE-derived spheroids faithfully recapitulate human interneuron development. In FES samples, both precocious cell cycle exit and persistent redox dyshomeostasis implicate early oxidative stress as a principal pathogenic mechanism driving accelerated cycling kinetics and altered neurodevelopmental trajectories in SCZ. This mechanistic insight is corroborated by a gestational hypoxia mouse paradigm that recapitulates diminished NPC proliferation, abbreviated cell cycles, and SCZ-relevant behaviors. NAC delivered during gestation fully rescues NPC proliferation, restores ~~pre~~frontal PV^+ interneuron density, and normalizes behavioral deficits, thereby indicating that redox-targeted intervention can correct cell-fate trajectories and circuit function. Extending NAC treatment to patient-derived brain organoids would permit validation in a human genomic context and establish a scalable platform for early-stage drug screening and individualized preventive therapy. Parallel efforts to generate vascularized, immunocompetent, and long-term sustainable brain organoids across diverse genetic backgrounds will further enhance the fidelity of SCZ neurodevelopmental models. Meanwhile, direct modulation of cell-cycle dynamics may offer an orthogonal avenue for safeguarding interneuron fate specification and aborting disease inception during the earliest windows of development.

4 | Methods

4.1 | Subjects

Approval for the study was granted by the Institutional Review Board of Affiliated Mental Health Center & Hangzhou Seventh People's Hospital, Zhejiang University School of Medicine (approval no. 2024-068), and West China Hospital, Sichuan University (approval no. 2018-120). All participants were Han Chinese and provided written informed consent. The individuals were assessed by one of two trained psychiatrists, both trained in administering the Structured Clinical Interview for the Diagnostic and Statistical Manual of Mental Disorders, Fourth Edition (DSM-IV) [117]. Four patients with FES were recruited from in-patient and out-patient psychiatric units and assessed by Structured Clinical Interview for the DSM-IV Axis I Disorder, Patient Edition (SCID-P) [118]. Age of onset was recorded as the onset of psychotic symptoms informed by the patients themselves or their informants. All the patients underwent the Positive and Negative Syndrome Scale (PANSS) test and were followed up for at least 6 months to confirm the diagnosis. A total of 6 HCs were screened using the non-patient edition of the SCID (SCID-NP) by one of the two qualified psychiatrists. Subjects with a previous history of any major psychiatric disorders, schizoaffective disorders, substance abuse, neurological disorders, head trauma, or a known family history of psychiatric disorders were excluded.

4.2 | Generation of iPSC~~Ipsc~~ Lines

All procedures followed the Institutional Review Board's guidelines, and all human samples were obtained with informed consent. PBMCs were collected, and serum was separated from the whole blood of all subjects. The following pathogens in serological samples were detected by the ELISA method: human immunodeficiency virus (HIV), Hepatitis B virus, Treponema pallidum specific antibody, and Hepatitis C virus (Table S3). Followed by PBMC resuscitation and expansion, $CD34^+$ cells were purified and amplified on fibronectin (Corning, Corning, NY, USA)-coated plate in StemSpan SFEM media (StemCell Technologies, Vancouver, BC, CA) with EX-CYTE Growth Enhancement Media Supplement (1:1000, Millipore, Billerica, MA, USA), 2 mM GlutaMAX (Thermo Fisher, Waltham, MA, USA), 250 ng/mL Stem Cell Factor (SCF, PeproTech, Rocky Hill, NJ, USA), 250 ng/mL FMS-like tyrosine kinase 3 ligand (FLT3L, PeproTech, Rocky Hill, NJ, USA), 100 ng/mL thrombopoietin (TPO, PeproTech, Rocky Hill, NJ, USA), 20 ng/mL interleukin-3 (IL-3, PeproTech, Rocky Hill, NJ, USA), 50 ng/mL interleukin-6 (IL-6, PeproTech, Rocky Hill, NJ, USA) and 10 ng/mL soluble interleukin-6 receptor (sIL6-R, PeproTech, Rocky Hill, NJ, USA). After 7-day amplification, 1×10^6 $CD34^+$ cells were transfected by Episomal reprogramming vectors of pEP4EO2SEN2K, pEP4EO2SET2K, and pCEP4-M2L containing expression cassettes for *OCT4*, *SOX2*, *NANOG*, *LIN28*, *c-MYC*, *KLF4*, and *SV40LT* transgenes [119]. The transfected $CD34^+$ cells were maintained on fibronectin/Matrigel (Corning, Corning, NY, USA) -coated plate in DMEM/F12 media (Thermo Fisher, Waltham, MA, USA) supplemented with N2 (1%, Thermo Fisher, Waltham, MA, USA), B27 (2% Thermo Fisher, Waltham, MA, USA), basic fibroblast growth factor (bFGF, 100 ng/mL, PeproTech, Rocky Hill, NJ, USA), PD0325901 (0.5 μM, Stemgent, Lexington, MA, USA), CHIR99021 (3 μM, Stemgent, Lexington, MA, USA), A-83-01 (0.5 μM, Stemgent, Lexington, MA, USA), human leukemia inhibitory factor (hLIF, 1000 U/mL, Millipore, Billerica, MA, USA), and HA-100 (10 μM, Santa Cruz Biotechnology, Dallas, TX, USA). The culture medium was refreshed every day. The most productive well was selected for the bulk passage from nascent colonies to initiate the first passage (P1) of iPSC cultures. The generated colonies with typical ESC-like morphology (clearly defined border, prominent nucleoli, high nucleus-to-cytoplasm ratios, and tightly clustered small cells) were manually picked to new culture dishes for following identification and subsequent differentiation.

The prepared iPSC lines were maintained on Matrigel-coated plates in Essential 8 (E8) Medium (Thermo Fisher, Waltham, MA, USA). Every 3 to 4 days, cells reaching 80%–90% confluence were dissociated with Versene Solution (Thermo Fisher, Waltham, MA, USA) and then dispersed at a splitting ratio of 1:5 to 1:8 to ensure optimal growth. The ROCK inhibitor Y-27632 (10 μM, ApexBio, Boston, MA, USA) was added to the culture for 24 h to prevent single-cell-induced cell death after passaging. Mycoplasma was routinely tested once a week using a mycoplasma detection kit (Takara, Shiga, OSA, Japan). All chemicals used in this study are listed in Table S17.

4.3 | Identification of iPSC Pluripotency

The pluripotency of iPSCs at passages 6 and 20 was characterized by OCT4/TRA-1-60 immunostaining (see details in the Immunofluorescence section). The expression of pluripotency markers of *OCT4* and *NANOG* was evaluated by qPCR (primers listed in Table S17). To objectively reflect the pluripotency of the iPSC lines, ESC line H1 (WiCell, Madison, WI, USA, passage 40-50) was set as a positive control, and hMSCs as a negative control. Total RNA was isolated by TRIzol reagent (Invitrogen, Carlsbad, CA, USA), and 1 μg RNA was reverse transcribed as the template (First Strand cDNA Synthesis Kit, Thermo Fisher Scientific, Waltham, MA, USA). The qPCR was performed using the Maxima SYBR Green qPCR Master Mix (Thermo Fisher Scientific, Waltham, MA, USA) on a Bio-Rad iCycle iQ5 system (Bio-Rad, Hercules, California, USA). For fluorescence-activated cell sorting (FACS), single cells were suspended and blocked in PBS with 5% bovine serum albumin (BSA; Sigma-Aldrich, St Louis, MO, USA) and stained with pluripotency markers of SSEA4 and TRA-1-81 (antibody information listed in Table S17). The suspension was then sorted through Guava easyCyte 8 (Merck Millipore, Billerica, MA, USA), and the collected data were analyzed using Kaluza Analysis 1.5 software (Beckman Coulter, Brea, CA, USA). To examine the in vivo differentiation potential of iPSCs, a total of 5×10^6 cells were suspended in 30% Matrigel in DMEM/F12 solution and injected intramuscularly in the hind legs of 6- to 8-week-old immunocompromised SCID Beige mice. After 6–8 weeks, the teratomas were dissected

when reaching 1–2 cm in diameter and processed with HE staining. The endoderm, mesoderm, and ectoderm tissues were evaluated using Imager Z2 (Carl Zeiss, Baden-Württemberg, Oberkochen, Germany).

4.4 | Genomic Integrity of iPSCs and Their Parental PBMCs

To assess the number and morphology of chromosomes and investigate the genomic stability, standard G-banded karyotype analyses were conducted on all prepared iPSC lines. During metaphase, colchicine (100 μg/mL, Sangon Biotech, Shanghai, Shanghai, CN) was added to the cells and incubated for 1 h at 37°C to collapse mitotic spindles and prevent the completion of mitosis. After incubation in hypotonic solution (75 mM KCl, Sangon Biotech, Shanghai, Shanghai, CN) for 20–40 min at 37°C, suspended cells were fixed with a mixture of methanol (Sangon Biotech, Shanghai, Shanghai, CN) and glacial acetic acid (Sangon Biotech, Shanghai, Shanghai, CN) (3:1) until the supernatant became clear and the pellet turned white. A total of 20–30 randomly metaphase spreads were counted, and Giemsa staining (Sigma-Aldrich, St Louis, MO, USA) was performed. DNA was extracted and purified from these iPSC lines and their parental PBMCs. Subsequent STR analysis was performed in BGI Genomics Co., Ltd. (Shenzhen, Guangdong, CN). Totally 23 loci were tested to confirm identity (details were shown in Table S2). The matched points and identical sizes between iPSCs and their corresponding parental PBMCs substantiated their identity confirmation. To identify any possible residual plasmid-derived sequences, genomic DNA was extracted according to the TIANamp Genomic DNA Kit (TIANGEN, Beijing, Beijing, CN). The qPCR was used to detect the expression of *OCT4* and *gEBANA1* in all iPSC lines at passage 6 (primers were listed in Table S17). To draw a standard curve, the episomal reprogramming plasmid of pCEP4-EGFP was mixed with genomic DNA from MSCs in a gradient copy-number ratio of 0, 0.1, 1, 10, and 100. Human MSCs functioned as a negative control, given their absence of nucleic acid sequences derived from exogenous plasmid sources. The obtained standard curve was depicted as $y = 0.933x - 6.620$. The mRNA expression of *OCT4* in each sample was used as an internal reference gene to normalize the data. Relative expression of *gEbna1* was analyzed using the $2^{-\Delta\Delta Ct}$ method. The copy number of *gEbna1* for each sample was calculated according to the standard curve [120].

4.5 | WGS of Parental PBMCs and Derived iPSC Lines

To investigate whether patient-specific genetic variants exist in the FES group, WGS was performed in paired PBMCs and iPSCs at passage 20 from each subject. The sequencing was performed by OE Biotech Co., Ltd. (Shanghai, Shanghai, CN). Briefly, the genomic DNA was extracted by TruSeq Nano DNA LT Sample Prep Kit (Illumina, San Diego, CA, USA), and a total of 1 μg of qualified genome was randomly fragmented to 350–500 bp using S220 Focused-ultrasonicates (Covaris, Woburn, MA, USA). The DNA library was constructed by VAHTS Universal Plus DNA Library Prep Kit (ND617, VAZYME, Nanjing, Jiangsu, CN) for Illumina following the steps of end repair, 3′ end adenylation, sequence adapters ligation, fragment enrichment, and library pooling. The final libraries were sequenced on the HiSeq X Ten platform (Illumina Inc., San Diego, CA, USA), generating 150-bp paired-end reads.

Sequencing reads were aligned to the Genome Reference Consortium Human Build 38 patch release 13 (GRCh38.p13) reference genome, including alternative contigs and decoys, using the Burrows-Wheeler Aligner maximum Exact Matches program (BWA-MEM, version 0.7.17) [121]. The mean depth of coverage was over 29.1× across all samples. The mapped reads were further processed with the Sequence Alignment/Map tools (SAM, version 1.9, https://github.com/samtools/samtools) [122], Picard tools (version 4.2.0.0, http://broadinstitute.github.io/picard), and Genome Analysis Tool Kit (GATK, version 4.2.0.0, https://gatk.broadinstitute.org/hc/en-us) [123] to generate Binary Alignment/Map (BAM) format files. The analysis-ready BAM files were used as input files for germline variant calling by HaplotypeCaller in GATK. To reduce false-positive mutation calls, only mutants with a quality/depth ≥ 2.0 were retained. Identified variations including single nucleotide polymorphisms (SNPs) and insertions/deletions (InDels) were then annotated using ANNOVAR with annotation databases including RefSeq (https://www.ncbi.nlm.nih.gov/refseq/), 1000 Genomes (1000G, http://www.internationalgenome.org/), Exome Sequencing Project 6500 (ESP6500, http://evs.gs.washington.edu/EVS/), Genome Aggregation Database (gnomAD, http://www.gnomad-sg.org/), Sorting Intolerant from Tolerant (SIFT, http://sift-dna.org), ClinVar (https://www.ncbi.nlm.nih.gov/clinvar/), Polymorphism Phenotyping v2 (PolyPhen, http://genetics.bwh.harvard.edu/pph2/index.shtml), MutationTaster (http://www.mutationtaster.org/), the Catalogue of Somatic Mutations in Cancer (COSMIC, https://cancer.sanger.ac.uk/cosmic), Genome-Wide Association Studies Catalog (GWAS Catalog, https://www.ebi.ac.uk/gwas/), and Online Mendelian Inheritance in Man (OMIM, http://omim.org/). PCA was performed on genotype using Kinship-based Inference [124]. The plots of PC2 against PC1 serve as a description of genetic similarity between individuals.

4.6 | Differentiation of iPSCs Into cINs

An overview of the differentiation procedure is presented in Figure 1A [125]. For neuroectoderm induction, cells were treated with Dual-SMAD inhibitors (LDN193189 and SB431542). For MGE phenotype induction, the media were supplemented with Wnt inhibitor (IWP2, Selleck Chem, Houston, MA, USA) and SHH activator (SAG). FGF8 was added to induce the MGE phenotype at the expense of the CGE phenotype. The iPSC lines were dissociated with 0.05% trypsin-EDTA (Thermo Fisher, Waltham, MA, USA), and the suspension was blocked in DMEM (Thermo Fisher, Waltham, MA, USA) with 10% Fetal Bovine Serum (FBS, Thermo Fisher, Waltham, MA, USA). Dissociated iPSCs were resuspended and self-assembled as embryoid bodies in DMEM media with 15% knockout serum replacement (KSR, Thermo Fisher, Waltham, MA, USA), 55 μM β-Mercaptoethanol (Thermo Fisher, Waltham, MA, USA), 0.1 μ~~m~~M{{Tao Li: The reagent concentrations in the "Differentiation of iPSCs Into cINs" section were inadvertently reported as stock concentrations. They have now been

corrected to the corresponding final working concentrations.}} LDN193189 (Selleck Chem, Houston, MA, USA), 0.1 µmM SAG (Selleck Chem, Houston, MA, USA), 10 µmM SB431542 (MedChem, Monmouth Junction, NJ, USA), and 5 µmM IWP2 for the first week. Y-27632 (10 µM) was added to the culture on the first day of differentiation to avoid single-cell-induced cell death of iPSCs. For the second week, both SB431542 and IWP2 were removed. During the third week, the medium was changed to DMEM/F12 GlutaMAX medium with 1% N2, 2010 µg/mlM ascorbic acid (AA, Sigma-Aldrich, St Louis, MO, USA), 0.1 µmM SAG, and 100 ng/mL FGF8 (ProSpec, Rocky Hill, CT, USA). From the fourth week, the cells were maintained in a neurotrophic-rich medium with 2% B27 supplement, 10 ng/mL glial cell line-derived neurotrophic factor (GDNF, ProSpec, Rocky Hill, CT, USA), and 10 ng/mL brain-derived neurotrophic factor (BDNF, ProSpec, Rocky Hill, CT, USA) in DMEM/F12 GlutaMAX medium with equal volume of Neurobasal medium (Thermo Fisher, Waltham, MA, USA). Spheroids were placed on the orbital shaker at 80 rpm (Wiggens, Straubenhardt, BW, GER) from the beginning of week two to the end of week six. Mycoplasma was routinely tested once a week.

4.7 | Library Construction and scRNA-seq

The iPSCs (0 weeks) were dissociated when reaching 80%–90% confluence to obtain the single cells, as detailed in the "Differentiation of iPSCs into cINs" section. After 3-week and 8-week induction, spheroids were dissociated by 0.05% trypsin with 100 mM Trehalose (Thermo Fisher, Waltham, MA, USA) and 2 U/mL Turbo DNase (Thermo Fisher, Waltham, MA, USA). To eliminate the dead cell clusters, the resuspended cells were filtered through a 35-µm cell strainer cap (Corning, Corning, NY, USA). When cIN spheroids matured to 16 weeks, single cells were carefully collected by SoniConvert sonicator (SC-L1) with a C-Type Tissue Dissociation Kit (both from Doc-Sense, Chengdu, Sichuan, CN). Briefly, the spheroids were washed with chilled PBS three times before being sectioned into small pieces. After incubation with Buffer-C1 and Buffer-C2 at 37°C for 10 min, the chopped spheroids were dissociated by the sonicator for 5 s. The cell suspension was centrifuged at 300 g for 5 min at 37°C and resuspended in Buffer-C3. The dissociated single cells at different stages subsequently endured the library construction, and the detailed information on each line was listed in Table S6.

For SCRB-seq, libraries were constructed as previously described [126]. Briefly, single cells were manually picked up using a glass capillary pipette (Sigma-Aldrich, Natick, MA, USA) and transferred into cell lysis buffer to synthesize the first-strand cDNA. Then, second-strand cDNA was synthesized, amplified, and fragmented. The obtained cDNA was purified using XP magnetic beads (Beckman Coulter, Brea, CA, USA) and DNA Clean and Concentrator Kit-5 (Zymo, Orange County, CA, USA). After quantification, RNA-seq libraries were prepared using the NEBNext Ultra II DNA Library Prep Kit (NEB, Beverly, MA, USA), following the instruction manual. For 10× Genomics, dissociated cells were resuspended in ice-cold PBS containing 0.04% BSA. The collected cells were isolated, encapsulated, and constructed using the Chromium Next GEM Single Cell 3' GEM, Library & Gel Bead Kit (V3.3, 10× Genomics, Pleasanton, CA, USA), following the manufacturer's instructions. The final cDNA libraries were sequenced with 150-bp paired-end reads on an Illumina HiSeq X-Ten instrument (Novogene, Beijing, Beijing, CN).

4.8 | Quality Assessment and Preprocessing of Single-Cell Sequence Data

The sequencing data processing from SCRB-seq was performed as previously described [126]. Raw sequencing reads were trimmed using Cutadapt (v1.15) to remove sequencing adapters, template switch oligo (TSO), and poly (A) tail sequences, [127] using the following parameters: -j 24 -m 50 –max-n = 0.1 -q 20,10 -O 5 -n 12. The trimmed reads were then aligned to GRCh38 using STAR (v2.6.0a) [128] with default parameters. The deduplication of a unique molecular identifier (UMI) was conducted on uniquely mapped reads using umi_tools (v1.0.0) with default parameters. Finally, the gene expression level of each cell was quantified according to barcodes using HTSeq (v0.11.2), [129] using the following parameters: -s no -f sam -a 10. The cells were classified as high quality if they met the following criteria: 1) the number of detected genes was between 1000 and 7000; 2) the number of detected UMIs was between 10 000 and 300 000; 3) the percentage of mitochondrial gene sequences was less than 15%; 4) mapping rates were more than 70%; and 5) cells with low percentage of External RNA Controls Consortium (ERCC) spike-ins, identified by function is Outlier (type = higher and nmads = 3) in scuttle R package (v1.0.4) [130].

For sequencing data generated by the 10× Genomics platform (Pleasanton, CA, USA), raw sequencing reads were aligned to GRCh38, and the count matrix was generated by Cell Ranger (v3.0.2, Pleasanton, CA, USA) [131] with default parameters. Similarly, cells were marked as high quality if they met the following criteria: 1) the number of detected genes was between 1, 000 and 6000; 2) the number of detected UMIs was between 1000 and 25 000; 3) the percentage of mitochondrial gene sequences was less than 10%.

4.9 | Processing and Functional Analysis of scRNA-seq Data

The downstream data analysis of scRNA-seq was mainly performed by Seurat (v3.2.3) [132]. Initially, raw counts were normalized using the *NormalizeData* (normalization.method = "LogNormalize") function with default parameters, and 2, 000 high variable genes (HVGs) were identified using the *FindVariableFeatures* with default parameters. The batch correction was then performed by *RunFastMNN* [133] with default parameters. Next, non-linear dimensional reduction was executed using *RunTSNE* (dims = 1:30), followed by cluster identification with *FindNeighbors* (dims = 1:30) and *FindClusters* (resolution = 0.5). Subsequently, the clusters with more than 50% of low-quality cells were removed. All the above processes were repeated for each cell population based on the enriched expression of selected markers. Then, the DEGs were identified using *FindAllMarkers* with default parameters, and the top 100 DEGs (selected by fold change) of each cluster were used to plot heatmap by *Pheatmap*. The DEGs were then utilized as the input for GO enrichment analysis, conducted with the R package

clusterProfiler (v3.18.0) [134]. GO terms were enriched by the 'compareCluster' function, and 'ont = BP' was set. To identify shared DEGs, the *FindMarkers* function was utilized with default parameters, and genes were categorized as either Up or Down based on $\log_2 FC > 0$.

4.10 | Alignment of Cells From MGE-Derived Spheroids to Databases of Human MGE-Derived Neurons

To align our transcriptional data with developmental stage-dependent gene expression changes during human neuronal differentiation, we used the CORTECON dataset as an external temporal reference. CORTECON is a published temporal RNA-seq resource generated from human embryonic stem cell-derived in vitro cerebral cortical differentiation [135]. CORTECON was used in this study as a broad developmental reference to contextualize stage-dependent transcriptional dynamics and the developmental timing of cortical interneuron lineage-related programs in our MGE-derived spheroids. Average gene expression levels at each time point were extracted, and $\log_2$-transformed fold changes [$\log_2$ (FC + 1×10^{-5})] were calculated for downstream analysis. To evaluate the developmental stage of the MGE-derived NPCs, raw counts were obtained from the Gene Expression Omnibus (GEO) to assess neural NPCs derived from iPSCs. Then, the data were processed, and NPCs were defined following the methods outlined in Yu et al. (GW9-12) [51]. Then, the reference NPCs Seurat object and MGE-derived NPCs Seurat objects were merged, and the data integration was done by *RunFastMNN* with 2000 highly variable features. To visualize and explore datasets in low-dimensional space *RunTSNE* was performed with reduction = mnn and dims = 1:20. Similarly, the scRNA-seq data of the human fetal brain from the late first to the early second trimester of human development (GW9-18) [52] were used as reference data. The data integration and dimensionality reduction were performed using the same parameters. Then, data visualization was performed in R (v4.1.2) and RStudio. Moreover, the MGE and 16-week-cINs were extracted from the Seurat object and converted into *SingleCellExperiment* objects. Subsequently, *scmapCluster* (threshold = 0.01) was used to project the 16-week-cINs dataset into the MGE dataset.

4.11 | GSEA

The files of count expression (txt format), phenotype labels (cls format), and gene sets (gmt format) were created manually by customized R and in-house Shell scripts. In brief, the raw count expression data were extracted from the Seurat object, focusing specifically on NPCs and 16-week cINs. Genes expressed in at least one cell were retained in the count matrix, written in table format along with the corresponding annotations, and manually modified to the format of GSEA files. Then the genes of top GO terms were extracted from R package org.Hs.eg.db (v3.12.0). The gene set files (gmt format) were created following the GSEA User Guide. Finally, the GSEA was done by the application for Linux [136, 137] with default parameters, and the results were visualized using R package GseaVis (v0.0.8).

4.12 | Calculation of Cell Type-Disease Association

To identify the association between cell subtypes and common psychiatric diseases including SCZ, bipolar disorder (BD), and major depressive disorder (MDD), a set of putative disease genes from GWAS data [74, 138, 139] constructed by scDRS using Multi-marker Analysis of GenoMic Annotation (MAGMA, https://doi.org/10.1371/journal.pcbi.1004219) [140] were obtained firstly from Zhang et al. (2022) [73]. With the normalized expression data of the AnnData object converted from the Seurat object, the analysis of the *compute-score* was subsequently performed with modified parameters (*–flag-filter-data False* and *–flag-raw-count False*). A raw disease score and 1000 control scores for each cell were generated and then,, the cell type-disease association was assessed by *perform-downstream* (–group-analysis) to aggregate the disease relevance. The results were visualized using the functions *sc.pl.tsne* and *scdrs.util.plot_group_stats*.

4.13 | Pseudotime Reconstruction and Alignment

To establish the cell trajectory, the raw counts were initially downsampled and then used to create the Monocle2 object [141] using the function *newCellDataSet*. To order the cells, a gene list was generated using the *FindAllMarkers* function (only.pos = T, logfc.threshold = 0.8 and min.diff.pct = 0.1) and used as input for *orderCells*. Then, the starting branch for trajectory was chosen manually by the position of the earliest developmental cell type (generally iPSCs). The pseudotime trajectory was then visualized on the reduced dimensional space by *plot_complex_cell_trajectory*.

Monocle3 was used to construct the trajectories of cINs and NPCs. Briefly, the dimensionality reduction was performed using the *RunTSNE* (dims = 1:30). The Seurat object were converted to a CellDataSet object using the as.cell_data_set() function, and *cluster_cells* (cluster_method = 'louvain") and *learn_graph* (use_partition = F, close_loop = F) functions were applied to order the cells in pseudotime. The pseudotime values for the cells were extracted from the CellDataSet objects and then rescaled from 0 to 100 to facilitate the creation of density plots.

Branch-dependent genes were identified by *Branch Expression Analysis Modeling* (*BEAM*) in Monocle2, [142] with a q-value cut-off set at less than 1×10^{-4}. *Plot_pseudotime_heatmap* was used to plot the expressive change of the selected genes along the pseudotime. Then the pseudotime was scaled as 0~1, and the dynamic time warping (DTW) algorithm was used to align the pseudotime courses of FES and HC groups according to the published method [143]. The F-test-based ANOVA analysis was used to identify the dysregulated genes along the aligned pseudotime trajectory. In brief, a natural splined linear regression model was established for each branch-dependent gene, and the F-test was applied to analyze the DEGs explained by the splined linear model (p-value < 0.05). And the GO terms were enriched by the *enrichGO* function, OrgDb = 'org.Hs.eg.db".

4.14 | Migration Analysis

For in vitro migration analysis, 8-week-old cIN spheroids were embedded in the Matrigel. Phase pictures were taken to follow the migration of cINs. To quantify the migration, ImageJ software was utilized to measure the migration distance in both the proximal and distal areas.

For in vivo migration analysis, complete cIN spheroids were transplanted into the hemispheres of NOD.Cg-Prkdc^scid/Il2rg^tm1Sug/JicCrl mice (NOG, Central Institute for Experimental Animals, Kanagawa, Tokyo, Japan). All animal procedures adhered to the approved guidelines and the animal protocols were approved by the Institutional Animal Care and Use Committee of Zhejiang University (approval no. ZJU20240667), and West China Hospital, Sichuan University (approval no. 20240809005). After 16 h of quercetin (40 ~~m~~μm, Sigma-Aldrich, St Louis, MO, USA) treatment, the 8-week-old cIN spheroids from two groups were transplanted into different hemispheres of the same mouse, respectively (left, FES; right, HC). Adult mice at 8–10 weeks were anesthetized by isoflurane (2% induction and followed by 0.5%–1% maintenance, RWD, Shenzhen, Guangdong, CN) using a stereotaxometer (RWD, Shenzhen, Guangdong, CN). A circular burr hole, with a radius of 0.75 mm and centered at coordinates (ML = ± 1.00 mm, AP = −3.20 mm), was surgically created on the skulls of mice using a microdrill (RWD, Shenzhen, Guangdong, CN). Through the burr hole, the cortical brain tissues with a diameter of approximately 1.5 mm were gently extracted using a vacuum pump. Spheroids with a diameter of less than 1.5 mm were gently placed into the prepared cavity. Finally, the transparent round coverslip (diameter = 4 mm, Schleden, Nantong, Jiangsu, CN) was affixed to the burr hole to provide physical protection to the brain. Recipient mice were sacrificed at 10 weeks post-transplantation. Briefly, mice were perfused with 4% paraformaldehyde (PFA; BBI Life Sciences, Shanghai, Shanghai, CN) solution, and their isolated brain tissues were subsequently fixed in 4% PFA for an additional 24 h. The post-fixation brains were dehydrated by 30% sucrose for 24 h and then coronally sectioned at a thickness of 30 μm with a Leica sliding microtome. The immunofluorescence staining process followed the same procedure as described above. For in vivo migration analysis, hNuclei staining was captured and tiled using Imager Z2 Microscopes with a 10× objective. The grafted cell number was counted using the multi-point function in ImageJ software (Version 1.53C, NIH, Bethesda, MD). Migrated cells (human Nuclei$^+$) from the graft border less than 400 μm and more than 400 μm were counted separately for each graft. The experimenters responsible for counting were blind to the experimental samples. The percentage of positive cells for each marker was calculated based on the number of DAPI-stained nuclei from three independent differentiations. At least 500 cells were counted for each marker.

4.15 | Ultrastructural Analysis by Tem

The 14-week-old cIN spheroids were fixed in 2.5% glutaraldehyde (pH 7.4, SPI-chem, Westchester, PA, USA) for 24 h. After being washed with 0.1 M phosphate buffer (prepared from sodium dihydrogen phosphate A600878 and disodium hydrogen phosphate A610404, PH 7.2, 15 min, 3 times, Sangon Biotech, Shanghai, Shanghai, CN), the samples were fixed in 1% osmic acid (Ted Pella Inc Reading, Altadena, CA, USA) at 4°C for 2 h. The fixed samples were then gradient dehydrated in a graded series of ethanol (30%–50%–70%–80%–95%–100%–100%). Subsequently, the spheroids were embedded in SPI-Pon 812 resin (SPI-chem, Westchester, PA, USA) for penetration and polymerization. Semithin sections (1 μm) were used to locate the region of interest, and then ultrathin sections (70 nm) were obtained by Leica UC7 (Leica Biosystem, Buffalo Grove, IL, USA). The sections were retrieved onto 150-mesh copper grids (Zhongjingkeyi, Beijing, Beijing, CN) for microstructure analysis. Following the counterstaining of 3% uranyl acetate (Ted Pella Inc., Reading, Altadena, CA, USA) and 0.3% lead citrate (Ted Pella Inc., Reading, Altadena, CA, USA), the sections were observed with the HT7800 transmission electron microscope (HITACHI, Hitachi, Tokyo, Japan).

4.16 | Calcium Imaging

The iPSC-derived spheroids, at 18 weeks old, were infected with an adeno-associated viral vector expressing GCaMP6s (AAV-hSyn-GCaMP6s, MOI = 2, Shumi, Wuhan, Hubei, CN) for 1 day. The infected spheroids were rinsed with PBS and maintained in fresh media. They were subsequently plated onto a glass-bottom 96-well multi-plate (Eppendorf, Barkhausenweg, Hamburg, DE) coated with 1% Matrigel and incubated at 37°C for 1 h. After 20 days, the calcium imaging was recorded for 200 s. One thousand frames were acquired at 28 Hz with a region of 1936 × 1460 pixels (4× magnification) under the stream acquisition mode. Changes in fluorescence intensity were measured using an Axio Observer 3 microscope (Carl Zeiss, Baden-Württemberg, Oberkochen, DE) equipped with an Axiocam 503 mono Camera (Carl Zeiss, Baden-Württemberg, Oberkochen, DE) and Colibri 7 Type RGB-UV Light Source (Carl Zeiss, Baden-Württemberg, Oberkochen, DE). ZEN Image Analysis Software (Carl Zeiss, Baden-Württemberg, Oberkochen, DE) was used to analyze the live calcium traces. Images were processed using ImageJ and custom-written routines in MATLAB 7.2 (Mathworks, Natick, MA, USA). The amplitude of signals was presented as fluorescence intensity after background subtraction. The threshold for calcium spikes was set at the 95th percentile of the amplitude of all detected events.

4.17 | MEA Recording of Spheroid Activity

MEA was performed using the Maestro Edge system (Axion Biosystems, Atlanta, GA, USA), and the data were analyzed with Axion Integrated Studio AxIS 2.4 (Axion Biosystems Inc., Atlanta, GA, USA) and NeuroExplorer (NexTechnologies, Madison, AL, USA). Briefly, the MGE-derived spheroids at 24 weeks were plated onto CytoView 48-well plates (Axion Biosystems, Atlanta, GA, USA) coated with polyethyleneimine (PEI, 0.1%, Sigma-Aldrich, St Louis, MO, USA) and laminin (10 μg/mL, Sigma-Aldrich, St Louis, MO, USA). The spheroids were maintained in the plate for 24 days, with the medium changed every other day. Before the recording, MEA plates were

allowed to equilibrate for 10 min in the Maestro (Axion Biosystems, Atlanta, GA, USA). Simultaneous signals from 16 extracellular electrodes per well were recorded under a constant temperature of 37°C. All channels were simultaneously sampled with a gain of 1200× and a frequency of 12.5 kHz/channel, using a band-pass filter set between 200 and 500 Hz. The signals were continuously recorded for 5 min. The detection threshold was set as +5.5 × standard deviation (SD), and no additional spike sorting techniques were applied. Active electrodes were defined as >1 spike over a 200-sec analysis period. The weighted mean firing rate was characterized by the number of spikes, normalized by the duration of the recording session. Network bursts were defined as events where more than 50 spikes occurred within a 100-millisecond window and were detected across 35% of the electrodes simultaneously. The burst frequency was calculated by dividing the total number of single-electrode bursts by the analysis period. The synchrony index, which quantifies synchronization without units, is defined in such a way that a value close to 1 indicates greater synchronization [144].

4.18 | Gestational Hypoxia Modeling and Cell Cycle Labeling

To establish an embryonic oxidative stress model, time-mated C57BL/6J pregnant mice were checked daily for vaginal plugs; plug presence was designated embryonic day 0.5 (E0.5). At E9.5, pregnant dams were randomly assigned to hypoxia or normoxia groups. The hypoxia group was placed in a chamber (PEIJIA, Hangzhou, Zhejiang, CN) with 10% O_2 for 48 h, while controls remained in ambient air. To assess embryonic cell proliferation and cell cycle dynamics, a dual pulse labeling strategy using EdU (Thermo Fisher, Waltham, MA, USA) and BrdU (Thermo Fisher, Waltham, MA, USA) was employed. At E12.5 and E15.5, pregnant mice received intraperitoneal injections of EdU (i.p., 40 mg/kg) 3 h before perfusion, followed by BrdU (i.p., 50 mg/kg) 1 h before perfusion. Embryos were harvested post-maternal perfusion for subsequent proliferative activity and S-phase progression analysis.

4.19 | Immunofluorescence

Immunofluorescence staining was performed on adherent cells, neural spheroids, as well as on cryosections of mouse brain tissue. Adherent cell or neural spheroid samples were fixed with 4% PFA for 15 min at room temperature, and the spheroids were cryoprotected in 30% sucrose at 4°C until they sank to the bottom. Mice were deeply anesthetized via intraperitoneal injection of tribromoethanol (250 mg/kg body weight, AibeiBio, Nanjing, Jiangsu, CN), with the absence of toe pinch reflex confirmed before perfusion. Transcardial perfusion was conducted through the left ventricle using ice-cold PBS (pH 7.4) to flush blood, followed by 4% PFA in PBS for fixation. Approximately 50 mL total volume (PBS + 4% PFA) was administered per mouse. After perfusion, brains were carefully dissected, post-fixed in 4% PFA at 4 °C for 24 h. After fixation, the neural spheroids/brain tissues were cryoprotected in 30% sucrose solution at 4°C until they sank to the bottom. For cryosection preparation, mouse brain tissues or neural spheroids were embedded in O.C.T. compound (Sakura, Torrance, CA, USA) and sectioned using a Leica CM3050S cryostat. The section thickness was 16 μm for neural spheroids, 30 μm for embryonic brain, and 40 μm for adult mouse brain. Sections were circled using a hydrophobic barrier pen (Biosharp, Hefei, Anhui, CN) and permeabilized with PBS containing 0.2% Triton X-100 (Beyotime, Shanghai, Shanghai, CN) and 10% FBS for 30 min at room temperature.

EdU detection was performed using the Click-iT EdU Alexa Fluor Imaging Kit (Thermo Fisher, Waltham, MA, USA) according to the manufacturer's protocol. For BrdU staining, sections were pretreated with 1.3 M hydrochloric acid (HCl, Sinopharm Chemical Reagent, Shanghai, Shanghai, CN) at 37°C for 30 min to denature DNA and expose incorporated BrdU. After acid treatment, sections were washed three times with PBS containing 2% FBS and then incubated with 0.2 M sodium borate solution (Sinopharm Chemical Reagent, Shanghai, Shanghai, CN) at room temperature for 30 min to neutralize residual acid. This was followed by three additional washes with PBS containing 2% FBS. After blocking, samples were incubated overnight at 4°C with primary antibodies diluted in antibody dilution buffer (2% FBS in PBS). TUNEL staining was performed on mouse brain tissue using a commercial in situ cell death detection kit (Beyotime, Shanghai, Shanghai, CN) following the manufacturer's instructions. After primary antibody incubation, samples were washed with PBS and incubated with fluorescently labeled secondary antibodies and DAPI (Invitrogen, Carlsbad, CA, USA) at room temperature for 1 h under light-protected conditions. The sections were then mounted with Fluoromount-G (Southern Biotech, Birmingham, AL, USA) and imaged using a Zeiss Imager Z2 or Nikon N-SIM S confocal microscope. Detailed antibody information is listed in Table S17.

4.20 | qPCR analysis of Embryonic Mouse Basal Forebrain

Ventral forebrain tissues were microdissected from embryonic mouse brains at E12.5 and E15.5 under a stereomicroscope on ice. The forebrain was exposed by removing the overlying soft cranial tissue, and the basal region was carefully isolated using fine forceps and micro-scalpels to minimize contamination from surrounding regions. Total RNA was extracted and reverse-transcribed into cDNA according to the manufacturer's instructions. Quantitative PCR was performed using TB Green Premix Ex Taq II (Tli RNaseH Plus) (2×) in a total reaction volume of 25 μL, containing 12.5 μL TB Green Premix, 1.0 μL forward primer (10 μM), 1.0 μL reverse primer (10 μM), 2.0 μL cDNA template, and 8.5 μL nuclease-free water. The qPCR cycling conditions were as follows: initial denaturation at 95°C for 30 s, followed by 40 cycles of 95°C for 5 s and 60°C for 30 s, and a final melting curve analysis to verify amplification specificity. Gene expression levels were normalized to *Gapdh* and calculated using the $2^{-\Delta\Delta Ct}$ method. Primer sequences for target genes are listed in Table S17.

4.21 | Maternal Behavior Assessment

Maternal behavior was assessed during the peripartum and early postnatal periods to determine whether gestational hypoxia altered

lactational care. Nest-building performance was documented at E18.5 and P1. Nest quality at P1 was scored using a 1–5 scale based on the 3-day nest-building protocol, with higher scores indicating more complete and enclosed nest construction. Litter size was recorded at P1, and pup survival rate was calculated at P7 as the percentage of surviving pups relative to the number of pups present at P1.

Milk band scores were assessed at P1 as an indicator of recent milk intake. Scores were assigned as follows: 0, no visible milk band; 1, visible but small or poorly filled milk band; and 2, clearly filled milk band with a larger visible area, indicating sufficient recent milk intake. Pup retrieval behavior was evaluated at P1 by briefly separating three pups from the dam and placing them away from the nest. The latency to retrieve the first pup and the latency to retrieve all three pups back to the nest were recorded. All behavioral assessments were performed by investigators blinded to experimental groups.

4.22 | Behavioral Test

All behavioral tests, including the OFT and PPI tests, were performed in adult offspring at 6–8 weeks of age. Male and female offspring were not analyzed separately. The OFT was performed in a square arena (40 cm × 40 cm × 40 cm) divided into a central zone (20 cm × 20 cm) and a peripheral zone. Mice were acclimated to the testing environment for ≥ 1 h pre-experiment. The arena was wiped with 75% ethanol before each trial to eliminate odors and sanitize surfaces. Each mouse was gently placed in the center, and behavior was recorded for 10 min using the Anymaze video tracking system. PPI reflects sensorimotor gating function, which is a process typically deficient in SCZ patients. To evaluate PPI impairment in hypoxia-exposed mice, acoustic startle reflex testing was conducted. The testing environment maintained a stable temperature/humidity in a quiet room to minimize external interference. Pre-test calibration ensured acoustic stimuli at 74, 78, 82, and 120 dB. Mice were habituated to the testing room for at least 1 h before the experiment. During the test, each mouse was placed individually in a startle chamber with 65 dB background noise for 5 min of acclimation. A baseline session followed by five consecutive 120 dB startle pulses (40 ms duration; 15-s inter-trial interval) to measure startle responses. The PPI testing session comprised eight pseudorandomized trial blocks. Prepulses (74, 78, or 82 dB; 20 ms) preceded 120 dB startle pulses (40 ms) by 30 ms. Trials were presented with 15-s inter-trial intervals. Startle responses were automatically recorded, with PPI percentage calculated as: PPI (%) = [1 − (mean startle amplitude for pulse trials with prepulse / mean startle amplitude for pulse alone trials)] × 100%.

4.23 | Maternal N-Acetylcysteine Administration

To investigate the potential protective effect of NAC (MedChemExpress, Monmouth Junction, NJ, USA) against embryonic hypoxia, pregnant mice received daily oral administration of NAC dissolved in sterile PBS. The NAC solution was freshly prepared before each administration and pPH-adjusted to avoid excessive acidity. Using a dedicated gavage needle, mice were administered the solution once daily at a fixed time. Doses were calculated according to individual body weights measured immediately before administration. Treatment commenced at E11.5 and continued daily until either tissue collection or spontaneous delivery. Experimental groups received NAC at 100 mg/kg or 200 mg/kg, while the control group received an equivalent volume of sterile PBS.

4.24 | Statistical Analysis

The statistical analysis was conducted by GraphPad Prism 9 (GraphPad Software, La Jolla, CA, USA). A two-tailed, unpaired *t*-test was utilized to compare the differences between two groups, while gender comparisons were analyzed by the chi-square test.

Multiple group comparisons were conducted using analysis of variance (ANOVA). When $p<0.05$, the difference was considered statistically significant. Data are presented as the mean ± SEM. No statistical method was used to pre-determine the sample size. The experimental cohort for iPSCs was chosen based on our selection criteria (Han Chinese adolescent patients with FES vs. age- and gender-matched healthy controls) without randomization to reduce variation caused by age, ethnicity, and gender. Blinding was used during cell counting. Each statistical test was justified by the appropriate method to meet the assumptions of the tests, which were summarized in Table S18.

Author Contributions

P.N., Q.Z., Y.J., F.G., and T.L. designed the experiments. P.N., Q.Z., Y.J., H.Y., C.Z., X.T., S.G., J.Z., N.X., X.R., M.F.,L.Z. X.Q. and X.Y. conducted experiments, collected data, and analyzed the data. P.N., Q.Z., Y.J., X.T., S.G., S.C., F.G., and T.L. wrote the manuscript. W.G. reviewed data interpretation and manuscript contents. P.N., S.C., and T.L. supported this study financially.

Acknowledgements

This work was supported by the National Natural Science Foundation of China (82571737 [P.N.] and 82230046 [T.L.]), the Key R&D Program of Zhejiang (2025C02112 [P.N.]), the Key R&D by Hangzhou Science and Technology Bureau (20241203A14 [T.L.]), the Hangzhou Special Project for Biomedical and Health Industry (2024WJC035 [P.N.]), Zhejiang Key Laboratory of Clinical and Basic Research for Psychiatric Diseases (No. 2024ZY01010 and 2024E10107) and the Construction Fund of Key Medical Disciplines of Hangzhou (2025HZGF10 [T.L.]). This work was also partly supported by grants from the National Institute of Mental Health (MH133205 [S.C.] and MH131610 [S.C.]).

Ethics Statement

The human study was conducted in accordance with the principles of the Declaration of Helsinki and was approved by the Institutional Review Board of the Affiliated Mental Health Center & Hangzhou Seventh People's Hospital, Zhejiang University School of Medicine (approval no. 2024-068), and the Biomedical Ethics Subcommittee of West China Hospital, Sichuan University (approval no. 2018-120).

All animal procedures were conducted in accordance with institutional guidelines for the care and use of laboratory animals and were approved by the Laboratory Animal Welfare and Ethics Review Committee of Zhejiang University (approval no. ZJU20240667) and the Laboratory Animal Ethics Committee of West China Hospital, Sichuan University (approval no. 20240809005).

Conflicts of Interest

The authors declare no conflicts of interest.

Open Research

Data Availability Statement

The data that support the findings of this study are openly available in GEO at https://www.ncbi.nlm.nih.gov/geo/ , reference number GSE217934.

Supporting Information

Supporting file {{Tao Li: A wording error and an incorrect panel designation were identified in a Supplementary Figure legend. We have uploaded both a revised version with the corrections highlighted in blue and a clean version. These corrections do not affect the data or conclusions of the study.}}: advs76936-sup-0001-SuppMat.docx.

Supporting file {{Tao Li: A wording error and an incorrect panel designation were identified in a Supplementary Figure legend. We have uploaded both a revised version with the corrections highlighted in blue and a clean version. These corrections do not affect the data or conclusions of the study.}}: advs76936-sup-0001-SuppMat.docx.
1. supinfo/advs76936-sup-0001-SuppMat.docx

Supporting file {{Tao Li: The title label was inadvertently omitted in the original version of Figure S8. The figure has been replaced with a corrected version including the complete title label. }}: advs76936-sup-0002-FigureS1-S8.zip.

Supporting file {{Tao Li: The title label was inadvertently omitted in the original version of Figure S8. The figure has been replaced with a corrected version including the complete title label. }}: advs76936-sup-0002-FigureS1-S8.zip.
2. supinfo/advs76936-sup-0002-FigureS1-S8.zip

Supporting file {{Tao Li: Corrections have been made to Table S18 and the statistical information associated with Table S1, Figure 6I, and Figure S1E–F. Specifically:
1.To maintain consistency in data presentation, the variability easure for the age variable in Table S1 has been changed from standard deviation (SD) to standard error of the mean (SEM).
2.The SEM value for the hypoxia + saline group in Figure 6I has been corrected based on recalculation from the original dataset.
3.Based on recalculation from the original dataset, the statistical analysis presented in Figure S1E–F has been updated. Specifically, the normalized H1 values were excluded from the statistical analysis.
These corrections do not affect the conclusions of the study.
 }} {{Tao Li: Corrections have been made to Table S1. The demographic information of participants HC3 and HC6 was inadvertently exchanged and has now been corrected. All data presented throughout the manuscript were subsequently carefully reviewed, and no similar errors were identified. }}: advs76936-sup-0003-TableS1-S18.zip

Supporting file {{Tao Li: Corrections have been made to Table S18 and the statistical information associated with Table S1, Figure 6I, and Figure S1E–F. Specifically:
1.To maintain consistency in data presentation, the variability easure for the age variable in Table S1 has been changed from standard deviation (SD) to standard error of the mean (SEM).
2.The SEM value for the hypoxia + saline group in Figure 6I has been corrected based on recalculation from the original dataset.
3.Based on recalculation from the original dataset, the statistical analysis presented in Figure S1E–F has been updated. Specifically, the normalized H1 values were excluded from the statistical analysis.
These corrections do not affect the conclusions of the study.
 }} {{Tao Li: Corrections have been made to Table S1. The demographic information of participants HC3 and HC6 was inadvertently exchanged and has now been corrected. All data presented throughout the manuscript were subsequently carefully reviewed, and no similar errors were identified. }}: advs76936-sup-0003-TableS1-S18.zip
3. supinfo/advs76936-sup-0003-TableS1-S18.zip

Supporting file : advs76936-sup-0004-VideoS1.mp4.

Supporting file : advs76936-sup-0004-VideoS1.mp4.
4. supinfo/advs76936-sup-0004-VideoS1.mp4

References

1. S. Saha , D. Chant , J. Welham , and J. McGrath , " A Systematic Review of the Prevalence of Schizophrenia ," *PLoS Medicine* *2* , no. 5 (2005): 141 , https://doi.org/10.1371/journal.pmed.0020141 .

2. R. A. McCutcheon , T. Reis Marques , and O. D. Howes , " Schizophrenia—An Overview ," *JAMA Psychiatry* *77* , no. 2 (2020): 201 – 210 , https://doi.org/10.1001/jamapsychiatry.2019.3360 .

3. D. W. Chung , M. A. Geramita , and D. A. Lewis , " Synaptic Variability and Cortical Gamma Oscillation Power in Schizophrenia ," *American Journal of Psychiatry* *179* , no. 4 (2022): 277 – 287 , https://doi.org/10.1176/appi.ajp.2021.21080798 .

4. O. D. Howes and E. Shatalina , " Integrating the Neurodevelopmental and Dopamine Hypotheses of Schizophrenia and the Role of Cortical Excitation-Inhibition Balance ," *Biological Psychiatry* *92* , no. 6 (2022): 501 – 513 , https://doi.org/10.1016/j.biopsych.2022.06.017 .

5. A. Kepecs and G. Fishell , " Interneuron Cell Types Are Fit to Function ," *Nature* *505* , no. 7483 (2014): 318 – 326 , https://doi.org/10.1038/nature12983 .

6. R. Tremblay , S. Lee , and B. Rudy , " GABAergic Interneurons in the Neocortex: From Cellular Properties to Circuits ," *Neuron* *91* , no. 2 (2016): 260 – 292 , https://doi.org/10.1016/j.neuron.2016.06.033 .

7. T. Zhang , Y. Zeng , Y. Zhang , et al., " Neuron Type Classification in Rat Brain Based on Integrative Convolutional and Tree-based Recurrent Neural Networks ," *Scientific Reports* *11* , no. 1 (2021): 7291 , https://doi.org/10.1038/s41598-021-86780-4 .

8. K. T. Sultan , Z. Han , X.-J. Zhang , et al., " Clonally Related GABAergic Interneurons Do Not Randomly Disperse but Frequently Form Local Clusters in the Forebrain ," *Neuron* *92* , no. 1 (2016): 31 – 44 , https://doi.org/10.1016/j.neuron.2016.09.033 .

9. X.-H. Yao , M. Wang , X.-N. He , et al., " Electrical Coupling Regulates Layer 1 Interneuron Microcircuit Formation in the Neocortex ," *Nature Communications* *7* , no. 1 (2016): 12229 , https://doi.org/10.1038/ncomms12229 .

10. O. Marín , " Interneuron Dysfunction in Psychiatric Disorders ," *Nature Reviews Neuroscience* *13* , no. 2 (2012): 107 – 120 , https://doi.org/10.1038/nrn3155 .

11. J. Yang , X. Yang , and K. Tang , " Interneuron Development and Dysfunction ," *The FEBS Journal* *289* , no. 8 (2022): 2318 – 2336 , https://doi.org/10.1111/febs.15872 .

12. Z. Shao , H. Noh , W. Bin Kim , et al., " Dysregulated Protocadherin-Pathway Activity as an Intrinsic Defect in Induced Pluripotent Stem Cell-Derived Cortical Interneurons From Subjects With Schizophrenia ," *Nature Neuroscience* *22* , no. 2 (2019): 229 – 242 , https://doi.org/10.1038/s41593-018-0313-z .

13. P. Ni , C. Zhou , S. Liang , et al., " YBX1-Mediated DNA Methylation-Dependent SHANK3 Expression in PBMCs and Developing Cortical Interneurons in Schizophrenia ," *Advanced Science* *10* , no. 20 (2023): 2300455 , https://doi.org/10.1002/advs.202300455 .

14. D. V. Hansen , J. H. Lui , P. Flandin , et al., " Non-epithelial Stem Cells and Cortical Interneuron Production in the human Ganglionic Eminences ," *Nature Neuroscience* *16* , no. 11 (2013): 1576 – 1587 , https://doi.org/10.1038/nn.3541 .

15. M. F. Paredes , C. Mora , Q. Flores-Ramirez , et al., " Nests of Dividing Neuroblasts Sustain Interneuron Production for the Developing human Brain ," *Science* *375* , no. 6579 (2022): abk2346 , https://doi.org/10.1126/science.abk2346 .

16. F. Birey , J. Andersen , C. D. Makinson , et al., " Assembly of Functionally Integrated human Forebrain Spheroids ," *Nature* *545* , no. 7652 (2017): 54 – 59 , https://doi.org/10.1038/nature22330 .

17. Y. K. Wu , C. Miehl , and J. Gjorgjieva , " Regulation of Circuit Organization and Function Through Inhibitory Synaptic Plasticity ," *Trends in Neurosciences* *45* , no. 12 (2022): 884 – 898 , https://doi.org/10.1016/j.tins.2022.10.006 .

18. T. Ma , C. Wang , L. Wang , et al., " Subcortical Origins of Human and Monkey Neocortical Interneurons ," *Nature Neuroscience* *16* , no. 11 (2013): 1588 – 1597 , https://doi.org/10.1038/nn.3536 .

19. J. H. Cabungcal , D. S. Counotte , E. Lewis , et al., " Juvenile Antioxidant Treatment Prevents Adult Deficits in a Developmental Model of Schizophrenia ," *Neuron* *83* , no. 5 (2014): 1073 – 1084 , https://doi.org/10.1016/j.neuron.2014.07.028 .

20. G. E. Hardingham and K. Q. Do , " Linking Early-life NMDAR Hypofunction and Oxidative Stress in Schizophrenia Pathogenesis ," *Nature Reviews Neuroscience 17* , no. 2 (2016): 125 – 134 , https://doi.org/10.1038/nrn.2015.19 .

21. R. Srivastava , T. Faust , A. Ramos , K. Ishizuka , and A. Sawa , " Dynamic Changes of the Mitochondria in Psychiatric Illnesses: New Mechanistic Insights from Human Neuronal Models ," *Biological Psychiatry 83* , no. 9 (2018): 751 – 760 , https://doi.org/10.1016/j.biopsych.2018.01.007 .

22. D. C. Fuhrmann and B. Brüne , " Mitochondrial Composition and Function Under the Control of Hypoxia ," *Redox Biology 12* (2017): 208 – 215 , https://doi.org/10.1016/j.redox.2017.02.012 .

23. K. A. Kennedy , S. D. Sandiford , I. S. Skerjanc , and S. S. Li , " Reactive Oxygen Species and the Neuronal Fate ," *Cellular and Molecular Life Sciences 69* , no. 2 (2012): 215 – 221 , https://doi.org/10.1007/s00018-011-0807-2 .

24. J. Chiu and I. W. Dawes , " Redox Control of Cell Proliferation ," *Trends in Cellular Biology 22* , no. 11 (2012): 592 – 601 , https://doi.org/10.1016/j.tcb.2012.08.002 .

25. G. Zsurka and W. S. Kunz , " Mitochondrial Dysfunction and Seizures: The Neuronal Energy Crisis ," *Lancet Neurology 14* , no. 9 (2015): 956 – 966 , https://doi.org/10.1016/s1474-4422(15)00148-9 .

26. M. K. Li , R. J. Yu , K. L. Chen , et al., " Long-Term Real-Time Tracking of Morphology and Migration of Neuronal Cells Under Oxidative Stress ," *Chemical & Biomedical Imaging 3* , no. 3 (2025): 191 – 198 , https://doi.org/10.1021/cbmi.4c00074 .

27. J. H. Cabungcal , P. Steullet , H. Morishita , et al., " Perineuronal Nets Protect Fast-Spiking Interneurons Against Oxidative Stress ," *Proceedings of the National Academy of Sciences U S A 110* , no. 22 (2013): 9130 – 9135 , https://doi.org/10.1073/pnas.1300454110 .

28. O. Kann , I. E. Papageorgiou , and A. Draguhn , " Highly Energized Inhibitory Interneurons Are a central Element for Information Processing in Cortical Networks ," *Journal of Cerebral Blood Flow and Metabolism 34* , no. 8 (2014): 1270 – 1282 , https://doi.org/10.1038/jcbfm.2014.104 .

29. F. Reyes-Madrigal , P. León-Ortiz , X. Mao , R. Mora-Durán , and D. C. Shungu , " de la Fuente-Sandoval C. Striatal Glutathione in First-episode Psychosis Patients Measured in Vivo With Proton Magnetic Resonance Spectroscopy ," *Archives of Medical Research 50* , no. 4 (2019): 207 – 213 , https://doi.org/10.1016/j.arcmed.2019.08.003 .

30. T. K. Das , A. Javadzadeh , A. Dey , et al., " Antioxidant Defense in Schizophrenia and Bipolar Disorder: A Meta-analysis of MRS Studies of Anterior Cingulate Glutathione ," *Progress in Neuro-Psychopharmacology and Biological Psychiatry 91* (2019): 94 – 102 , https://doi.org/10.1016/j.pnpbp.2018.08.006 .

31. K. Q. Do , A. H. Trabesinger , and M. Kirsten-Krüger , " Schizophrenia: Glutathione Deficit in Cerebrospinal Fluid and Prefrontal Cortex in Vivo ," *European Journal of Neuroscience 12* , no. 10 (2000): 3721 – 3728 , https://doi.org/10.1046/j.1460-9568.2000.00229.x .

32. L. Xin , R. Mekle , M. Fournier , et al., " Genetic Polymorphism Associated Prefrontal Glutathione and Its Coupling with Brain Glutamate and Peripheral Redox Status in Early Psychosis ," *Schizophrenia Bulletin 42* , no. 5 (2016): 1185 – 1196 , https://doi.org/10.1093/schbul/sbw038 .

33. D. Shan , D. Mount , S. Moore , V. Haroutunian , J. H. Meador-Woodruff , and R. E. McCullumsmith , " Abnormal Partitioning of Hexokinase 1 Suggests Disruption of a Glutamate Transport Protein Complex in Schizophrenia ," *Schizophrenia Research 154* , no. 1-3 (2014): 1 – 13 , https://doi.org/10.1016/j.schres.2014.01.028 .

34. K. A. Barksdale , E. Perez-Costas , J. C. Gandy , M. Melendez-Ferro , R. C. Roberts , and G. N. Bijur , " Mitochondrial Viability in Mouse and human Postmortem Brain ," *The FASEB Journal 24* , no. 9 (2010): 3590 – 3599 , https://doi.org/10.1096/fj.09-152108 .

35. B. M. L. Baselmans , L. Yengo , W. van Rheenen , and N. R. Wray , " Risk in Relatives, Heritability, SNP-Based Heritability, and Genetic Correlations in Psychiatric Disorders: A Review ," *Biological Psychiatry 89* , no. 1 (2021): 11 – 19 , https://doi.org/10.1016/j.biopsych.2020.05.034 .

36. M. P. Forrest , H. Zhang , W. Moy , et al., " Open Chromatin Profiling in hiPSC-Derived Neurons Prioritizes Functional Noncoding Psychiatric Risk Variants and Highlights Neurodevelopmental Loci ," *Cell Stem Cell 21* , no. 3 (2017): 305 – 318 , https://doi.org/10.1016/j.stem.2017.07.008 .

37. E. K. Stachowiak , C. A. Benson , S. T. Narla , et al., " Cerebral Organoids Reveal Early Cortical Maldevelopment in Schizophrenia—Computational Anatomy and Genomics, Role of FGFR1 ," *Translational Psychiatry 7* , no. 11 (2017): 6 , https://doi.org/10.1038/s41398-017-0054-x .

38. K. Fujimori , M. Ishikawa , A. Otomo , et al., " Modeling Sporadic ALS in iPSC-Derived Motor Neurons Identifies a Potential Therapeutic Agent ," *Nature Medicine 24* , no. 10 (2018): 1579 – 1589 , https://doi.org/10.1038/s41591-018-0140-5 .

39. R. Hilker , D. Helenius , B. Fagerlund , et al., " Heritability of Schizophrenia and Schizophrenia Spectrum Based on the Nationwide Danish Twin Register ," *Biological Psychiatry 83* , no. 6 (2018): 492 – 498 , https://doi.org/10.1016/j.biopsych.2017.08.017 .

40. C. Pak , T. Danko , Y. Zhang , et al., " Human Neuropsychiatric Disease Modeling Using Conditional Deletion Reveals Synaptic Transmission Defects Caused by Heterozygous Mutations in NRXN1 ," *Cell Stem Cell* 17 , no. 3 (2015): 316 – 328 , https://doi.org/10.1016/j.stem.2015.07.017 .

41. M. Bershteyn , S. Bröer , M. Parekh , et al., " Human Pallial MGE-type GABAergic Interneuron Cell Therapy for Chronic Focal Epilepsy ," *Cell Stem Cell* 30 , no. 10 (2023): 1331 – 1350 , https://doi.org/10.1016/j.stem.2023.08.013 .

42. J. S. Schweitzer , B. Song , T. M. Herrington , et al., " Personalized iPSC-Derived Dopamine Progenitor Cells for Parkinson's Disease ," *New England Journal of Medicine* 382 , no. 20 (2020): 1926 – 1932 , https://doi.org/10.1056/NEJMoa1915872 .

43. T.-Y. Park , J. Jeon , N. Lee , et al., " Co-Transplantation of Autologous Treg Cells in a Cell Therapy for Parkinson's Disease ," *Nature* 619 , no. 7970 (2023): 606 – 615 , https://doi.org/10.1038/s41586-023-06300-4 .

44. Y. Liu , H. Liu , C. Sauvey , L. Yao , E. D. Zarnowska , and S.-C. Zhang , " Directed Differentiation of Forebrain GABA Interneurons From Human Pluripotent Stem Cells ," *Nature Protocols* 8 , no. 9 (2013): 1670 – 1679 , https://doi.org/10.1038/nprot.2013.106 .

45. A. M. Maroof , S. Keros , J. A. Tyson , et al., " Directed Differentiation and Functional Maturation of Cortical Interneurons From Human Embryonic Stem Cells ," *Cell Stem Cell* 12 , no. 5 (2013): 559 – 572 , https://doi.org/10.1016/j.stem.2013.04.008 .

46. P. Ni , H. Noh , Z. Shao , et al., " Large-Scale Generation and Characterization of Homogeneous Populations of Migratory Cortical Interneurons From Human Pluripotent Stem Cells ," *Molecular Therapy—Methods & Clinical Development* 13 (2019): 414 – 430 , https://doi.org/10.1016/j.omtm.2019.04.002 .

47. S. A. Sloan , J. Andersen , A. M. Pașca , F. Birey , and S. P. Pașca , " Generation and Assembly of Human Brain Region–Specific Three-Dimensional Cultures ," *Nature Protocols* 13 , no. 9 (2018): 2062 – 2085 , https://doi.org/10.1038/s41596-018-0032-7 .

48. M. B. Johnson and C. A. Walsh , " Cerebral Cortical Neuron Diversity and Development at Single-Cell Resolution ," *Current Opinion in Neurobiology* 42 (2017): 9 – 16 , https://doi.org/10.1016/j.conb.2016.11.001 .

49. C. Mayer , C. Hafemeister , R. C. Bandler , et al., " Developmental Diversification of Cortical Inhibitory Interneurons ," *Nature* 555 , no. 7697 (2018): 457 – 462 , https://doi.org/10.1038/nature25999 .

50. M. B. Miller , A. Y. Huang , J. Kim , et al., " Somatic Genomic Changes in Single Alzheimer's Disease Neurons ," *Nature* 604 , no. 7907 (2022): 714 – 722 , https://doi.org/10.1038/s41586-022-04640-1 .

51. Y. Yu , Z. Zeng , D. Xie , et al., " Interneuron Origin and Molecular Diversity in the human Fetal Brain ," *Nature Neuroscience* 24 , no. 12 (2021): 1745 – 1756 , https://doi.org/10.1038/s41593-021-00940-3 .

52. Y. Shi , M. Wang , D. Mi , et al., " Mouse and Human Share Conserved Transcriptional Programs for Interneuron Development ," *Science* 374 , no. 6573 (2021): abj6641 , https://doi.org/10.1126/science.abj6641 .

53. Y. Yu , Y. Lin , Y. Takasaki , et al., " Rare Loss of Function Mutations in N-methyl-D-aspartate Glutamate Receptors and Their Contributions to Schizophrenia Susceptibility ," *Translational Psychiatry* 8 , no. 1 (2018): 12 , https://doi.org/10.1038/s41398-017-0061-y .

54. S. M. Shin , S. Skaar , E. Danielson , and S. H. Lee , " Aberrant Expression of S-SCAM Causes the Loss of GABAergic Synapses in Hippocampal Neurons ," *Scientific Reports* 10 , no. 1 (2020): 83 , https://doi.org/10.1038/s41598-019-57053-y .

55. T.-G. Kim , R. Yao , T. Monnell , et al., " Efficient Specification of Interneurons From Human Pluripotent Stem Cells by Dorsoventral and Rostrocaudal Modulation ," *Stem Cells* 32 , no. 7 (2014): 1789 – 1804 , https://doi.org/10.1002/stem.1704 .

56. L. Lim , D. Mi , A. Llorca , and O. Marín , " Development and Functional Diversification of Cortical Interneurons ," *Neuron* 100 , no. 2 (2018): 294 – 313 , https://doi.org/10.1016/j.neuron.2018.10.009 .

57. S. E. Ahmari and S. J. Smith , " Knowing a Nascent Synapse When You See It ," *Neuron* 34 , no. 3 (2002): 333 – 336 , https://doi.org/10.1016/s0896-6273(02)00685-2 .

58. K. M. Harris and R. J. Weinberg , " Ultrastructure of Synapses in the Mammalian Brain ," *Cold Spring Harbor Perspectives in Biology* 4 , no. 5 (2012): a005587 – a005587 , https://doi.org/10.1101/cshperspect.a005587 .

59. F. Lepiemme , J. Stoufflet , M. Javier-Torrent , G. Mazzucchelli , C. G. Silva , and L. Nguyen , " Oligodendrocyte Precursors Guide Interneuron Migration by Unidirectional Contact Repulsion ," *Science* 376 , no. 6595 (2022): abn6204 , https://doi.org/10.1126/science.abn6204 .

60. A. Voronova , S. A. Yuzwa , B. S. Wang , et al., " Migrating Interneurons Secrete Fractalkine to Promote Oligodendrocyte Formation in the Developing Mammalian Brain ," *Neuron* 94 , no. 3 (2017): 500 – 516 , https://doi.org/10.1016/j.neuron.2017.04.018 .

61. D. I. Sinner , G. J. Kim , G. C. Henderson , and R. A. Igal , " StearoylCoA Desaturase-5: A Novel Regulator of Neuronal Cell Proliferation and Differentiation ," *PLoS One* *7* , no. 6 (2012): 39787 , https://doi.org/10.1371/journal.pone.0039787 .

62. S. R. Ferrón , M. Charalambous , E. Radford , et al., " Postnatal Loss of Dlk1 Imprinting in Stem Cells and Niche Astrocytes Regulates Neurogenesis ," *Nature* *475* , no. 7356 (2011): 381 – 385 , https://doi.org/10.1038/nature10229 .

63. J. H. Kim , H. G. Jung , A. Kim , et al., " Hevin–Calcyon Interaction Promotes Synaptic Reorganization After Brain Injury ," *Cell Death & Differentiation* *28* , no. 9 (2021): 2571 – 2588 , https://doi.org/10.1038/s41418-021-00772-5 .

64. V. Gongidi , C. Ring , M. Moody , et al., " SPARC-Like 1 Regulates the Terminal Phase of Radial Glia-Guided Migration in the Cerebral Cortex ," *Neuron* *41* , no. 1 (2004): 57 – 69 , https://doi.org/10.1016/s0896-6273(03)00818-3 .

65. J. van de Leemput , N. C. Boles , T. R. Kiehl , et al., " CORTECON: A Temporal Transcriptome Analysis of In Vitro human Cerebral Cortex Development From human Embryonic Stem Cells ," *Neuron* *83* , no. 1 (2014): 51 – 68 , https://doi.org/10.1016/j.neuron.2014.05.013 .

66. K. Letinic , R. Zoncu , and P. Rakic , " Origin of GABAergic Neurons in the Human Neocortex ," *Nature* *417* , no. 6889 (2002): 645 – 649 , https://doi.org/10.1038/nature00779 .

67. M. Brenner and A. Messing , " Regulation of GFAP Expression ," *ASN Neuro* *13* , no. 1 (2021): 1759091420981206 , https://doi.org/10.1177/1759091420981206 .

68. L. Yang , Z. Li , G. Liu , X. Li , and Z. Yang , " Developmental Origins of Human Cortical Oligodendrocytes and Astrocytes ," *Neuroscience Bulletin* *38* , no. 1 (2022): 47 – 68 , https://doi.org/10.1007/s12264-021-00759-9 .

69. P. Ni , H. Noh , G.-H. Park , et al., " iPSC-Derived Homogeneous Populations of Developing Schizophrenia Cortical Interneurons Have Compromised Mitochondrial Function ," *Molecular Psychiatry* *25* , no. 11 (2020): 2873 – 2888 , https://doi.org/10.1038/s41380-019-0423-3 .

70. Schizophrenia Working Group of the Psychiatric Genomics Consortium , " Biological Insights from 108 Schizophrenia-associated Genetic Loci ," *Nature* *511* , no. 7510 (2014): 421 – 427 , https://doi.org/10.1038/nature13595 .

71. A. Schulmann , E. Ryu , V. Goncalves , et al., " Novel Complex Interactions Between Mitochondrial and Nuclear DNA in Schizophrenia and Bipolar Disorder ," *Molecular Neuropsychiatry* *5* , no. 1 (2019): 13 – 27 , https://doi.org/10.1159/000495658 .

72. R. H. Williams and T. Riedemann , " Development, Diversity, and Death of MGE-Derived Cortical Interneurons ," *International Journal of Molecular Sciences* *22* , no. 17 (2021): 9297 , https://doi.org/10.3390/ijms22179297 .

73. M. J. Zhang , K. Hou , K. K. Dey , et al., " Polygenic Enrichment Distinguishes Disease Associations of Individual Cells in Single-Cell RNA-Seq Data ," *Nature Genetics* *54* , no. 10 (2022): 1572 – 1580 , https://doi.org/10.1038/s41588-022-01167-z .

74. A. F. Pardiñas , P. Holmans , A. J. Pocklington , et al., " Common Schizophrenia Alleles Are Enriched in Mutation-intolerant Genes and in Regions Under Strong Background Selection ," *Nat Genet* *50* , no. 3 (2018): 381 – 389 , https://doi.org/10.1038/s41588-018-0059-2 .

75. J. E. Lisman , J. T. Coyle , R. W. Green , et al., " Circuit-based Framework for Understanding Neurotransmitter and Risk Gene Interactions in Schizophrenia ," *Trends in Neurosciences* *31* , no. 5 (2008): 234 – 242 , https://doi.org/10.1016/j.tins.2008.02.005 .

76. P. Flandin , Y. Zhao , D. Vogt , et al., " Lhx6 and Lhx8 Coordinately Induce Neuronal Expression of Shh That Controls the Generation of Interneuron Progenitors ," *Neuron* *70* , no. 5 (2011): 939 – 950 , https://doi.org/10.1016/j.neuron.2011.04.020 .

77. E. Azim , D. Jabaudon , R. M. Fame , and J. D. Macklis , " SOX6 controls Dorsal Progenitor Identity and Interneuron Diversity During Neocortical Development ," *Nature Neuroscience* *12* , no. 10 (2009): 1238 – 1247 , https://doi.org/10.1038/nn.2387 .

78. D. Vogt , R. F. Hunt , S. Mandal , et al., " Lhx6 Directly Regulates Arx and CXCR7 to Determine Cortical Interneuron Fate and Laminar Position ," *Neuron* *82* , no. 2 (2014): 350 – 364 , https://doi.org/10.1016/j.neuron.2014.02.030 .

79. H. H. Cho , F. Cargnin , Y. Kim , et al., " Isl1 directly Controls a Cholinergic Neuronal Identity in the Developing Forebrain and Spinal Cord by Forming Cell Type-specific Complexes ," *PLos Genetics* *10* , no. 4 (2014): 1004280 , https://doi.org/10.1371/journal.pgen.1004280 .

80. C. Zhou , G. Yang , M. Chen , et al., " Lhx6 and Lhx8: Cell Fate Regulators and Beyond ," *FASEB Journal* *29* , no. 10 (2015): 4083 – 4091 , https://doi.org/10.1096/fj.14-267500 .

81. W. Dai , R. Guo , X. Na , et al., " Hypoxia and the Endometrium: An Indispensable Role for HIF-1α as Therapeutic Strategies ," *Redox Biology* *73* (2024): 103205 , https://doi.org/10.1016/j.redox.2024.103205 .

82. W. C. Burhans and N. H. Heintz , " The Cell Cycle Is a Redox Cycle: Linking Phase-specific Targets to Cell Fate ," *Free Radical Biology &*

Medicine *47* , no. 9 (2009): 1282 – 1293 , https://doi.org/10.1016/j.freeradbiomed.2009.05.026 .

83. O. Coqueret , " New Roles for p21 and p27 Cell-Cycle Inhibitors: A Function for Each Cell Compartment? ," *Trends in Cellular Biology* *13* , no. 2 (2003): 65 – 70 , https://doi.org/10.1016/s0962-8924(02)00043-0 .

84. D. W. Volk , T. Matsubara , S. Li , et al., " Deficits in Transcriptional Regulators of Cortical Parvalbumin Neurons in Schizophrenia ," *American Journal of Psychiatry* *169* , no. 10 (2012): 1082 – 1091 , https://doi.org/10.1176/appi.ajp.2012.12030305 .

85. A. Mukherjee , F. Carvalho , and S. Eliez , " Long-Lasting Rescue of Network and Cognitive Dysfunction in a Genetic Schizophrenia Model ," *Cell* *178* , no. 6 (2019): 1387 – 1402 , https://doi.org/10.1016/j.cell.2019.07.023 .

86. D. W. Chung , D. W. Volk , D. Arion , Y. Zhang , A. R. Sampson , and D. A. Lewis , " Dysregulated ErbB4 Splicing in Schizophrenia: Selective Effects on Parvalbumin Expression ," *American Journal of Psychiatry* *173* , no. 1 (2016): 60 – 68 , https://doi.org/10.1176/appi.ajp.2015.15020150 .

87. E. M. Goldberg , H. Y. Jeong , I. Kruglikov , R. Tremblay , R. M. Lazarenko , and B. Rudy , " Rapid Developmental Maturation of Neocortical FS Cell Intrinsic Excitability ," *Cerebral Cortex* *21* , no. 3 (2011): 666 – 682 , https://doi.org/10.1093/cercor/bhq138 .

88. D. Doischer , J. A. Hosp , Y. Yanagawa , et al., " Postnatal Differentiation of Basket Cells From Slow to Fast Signaling Devices ," *The Journal of Neuroscience* *28* , no. 48 (2008): 12956 – 12968 , https://doi.org/10.1523/jneurosci.2890-08.2008 .

89. O. Marín , " Parvalbumin Interneuron Deficits in Schizophrenia ," *European Neuropsychopharmacology* *82* (2024): 44 – 52 , https://doi.org/10.1016/j.euroneuro.2024.02.010 .

90. R. N. Delgado , D. E. Allen , M. G. Keefe , et al., " Individual human Cortical Progenitors Can Produce Excitatory and Inhibitory Neurons ," *Nature* *601* , no. 7893 (2022): 397 – 403 , https://doi.org/10.1038/s41586-021-04230-7 .

91. K. Lu , Y. Hong , M. Tao , et al., " Depressive Patient-Derived GABA Interneurons Reveal Abnormal Neural Activity Associated With HTR2C ," *EMBO Molecular Medicine* *15* , no. 1 (2023): 16364 , https://doi.org/10.15252/emmm.202216364 .

92. Y. Xiang , Y. Tanaka , B. Patterson , et al., " Fusion of Regionally Specified hPSC-Derived Organoids Models Human Brain Development and Interneuron Migration ," *Cell Stem Cell* *21* , no. 3 (2017): 383 – 398 , https://doi.org/10.1016/j.stem.2017.07.007 .

93. L. Ma , Y. Du , Y. Hui , et al., " Developmental Programming and Lineage Branching of Early Human Telencephalon ," *The EMBO Journal* *40* , no. 21 (2021): 107277 , https://doi.org/10.15252/embj.2020107277 .

94. M. Torigoe , K. Yamauchi , Y. Zhu , H. Kobayashi , and F. Murakami , " Association of Astrocytes With Neurons and Astrocytes Derived From Distinct Progenitor Domains in the Subpallium ," *Scientific Reports* *5* , no. 1 (2015): 12258 , https://doi.org/10.1038/srep12258 .

95. L. Sussel , O. Marin , S. Kimura , and J. L. Rubenstein , " Loss of Nkx2.1 Homeobox Gene Function Results in a Ventral to Dorsal Molecular Respecification Within the Basal Telencephalon: Evidence for a Transformation of the Pallidum Into the Striatum ," *Development* *126* , no. 15 (1999): 3359 – 3370 , https://doi.org/10.1242/dev.126.15.3359 .

96. M. Sandberg , P. Flandin , S. Silberberg , et al., " Transcriptional Networks Controlled by NKX2-1 in the Development of Forebrain GABAergic Neurons ," *Neuron* *91* , no. 6 (2016): 1260 – 1275 , https://doi.org/10.1016/j.neuron.2016.08.020 .

97. P. Liodis , M. Denaxa , M. Grigoriou , C. Akufo-Addo , Y. Yanagawa , and V. Pachnis , " Lhx6 activity Is Required for the Normal Migration and Specification of Cortical Interneuron Subtypes ," *The Journal of Neuroscience* *27* , no. 12 (2007): 3078 – 3089 , https://doi.org/10.1523/jneurosci.3055-06.2007 .

98. S. Hijazi , A. B. Smit , and R. E. van Kesteren , " Fast-Spiking Parvalbumin-Positive Interneurons in Brain Physiology and Alzheimer's Disease ," *Molecular Psychiatry* *28* (2023): 4954 – 4967 , https://doi.org/10.1038/s41380-023-02168-y .

99. M. P. Jadi , M. M. Behrens , and T. J. Sejnowski , " Abnormal Gamma Oscillations in N-Methyl-D-Aspartate Receptor Hypofunction Models of Schizophrenia ," *Biological Psychiatry* *79* , no. 9 (2016): 716 – 726 , https://doi.org/10.1016/j.biopsych.2015.07.005 .

100. M. T. Koh , Y. Shao , A. Sherwood , and D. R. Smith , " Impaired Hippocampal-Dependent Memory and Reduced Parvalbumin-Positive Interneurons in a Ketamine Mouse Model of Schizophrenia ," *Schizophrenia Research* *171* , no. 1-3 (2016): 187 – 194 , https://doi.org/10.1016/j.schres.2016.01.023 .

101. T. Sawada , A. R. Barbosa , B. Araujo , et al., " Recapitulation of Perturbed Striatal Gene Expression Dynamics of Donors" Brains With Ventral Forebrain Organoids Derived From the Same Individuals With Schizophrenia ," *American Journal of Psychiatry* *181* , no. 6 (2024): 493 – 511 , https://doi.org/10.1176/appi.ajp.20220723 .

102. D. W. Meechan , E. S. Tucker , T. M. Maynard , and A. S. LaMantia , " Cxcr4 regulation of Interneuron Migration Is Disrupted in 22q11.2 Deletion Syndrome ," *Proceedings of the National Academy of Sciences U S A 109* , no. 45 (2012): 18601 – 18606 , https://doi.org/10.1073/pnas.1211507109 .

103. K. Brennand , J. N. Savas , Y. Kim , et al., " Phenotypic Differences in hiPSC NPCs Derived From Patients With Schizophrenia ," *Molecular Psychiatry 20* , no. 3 (2015): 361 – 368 , https://doi.org/10.1038/mp.2014.22 .

104. C. Bressan and A. Saghatelyan , " Intrinsic Mechanisms Regulating Neuronal Migration in the Postnatal Brain ," *Frontiers in Cellular Neuroscience 14* (2020): 620379 , https://doi.org/10.3389/fncel.2020.620379 .

105. S. Rumpf , N. Sanal , and M. Marzano , " Energy Metabolic Pathways in Neuronal Development and Function ," *Oxford Open Neuroscience 2* (2023): kvad004 , https://doi.org/10.1093/oons/kvad004 .

106. A.-H. BJS , E. Oler , B. Armistead , et al., " The Fetal Origins of Mental Illness ," *American Journal of Obstetrics & Gynecology 221* , no. 6 (2019): 549 – 562 , https://doi.org/10.1016/j.ajog.2019.06.013 .

107. C. Davies , G. Segre , A. Estradé , et al., " Prenatal and Perinatal Risk and Protective Factors for Psychosis: A Systematic Review and Meta-Analysis ," *The Lancet Psychiatry 7* , no. 5 (2020): 399 – 410 , https://doi.org/10.1016/s2215-0366(20)30057-2 .

108. V. Paquin , M. Lapierre , F. Veru , and S. King , " Early Environmental Upheaval and the Risk for Schizophrenia ," *Annual Review of Clinical Psychology 17* , no. 1 (2021): 285 – 311 , https://doi.org/10.1146/annurev-clinpsy-081219-103805 .

109. Y. Mao , X. Ge , C. L. Frank , et al., " Disrupted in Schizophrenia 1 Regulates Neuronal Progenitor Proliferation via Modulation of GSK3β/β-Catenin Signaling ," *Cell 136* , no. 6 (2009): 1017 – 1031 , https://doi.org/10.1016/j.cell.2008.12.044 .

110. D. van der Meer , W. Cheng , J. Rokicki , et al., " Clustering Schizophrenia Genes by Their Temporal Expression Patterns Aids Functional Interpretation ," *Schizophrenia Bulletin 50* , no. 2 (2024): 327 – 338 , https://doi.org/10.1093/schbul/sbad140 .

111. T. Shen , J. Yu , B. Xie , et al., " Protein Arginine Methyltransferase 7 Linked to Schizophrenia Through Regulation of Neural Progenitor Cell Proliferation and Differentiation ," *Cell Reports 44* , no. 2 (2025): 115279 , https://doi.org/10.1016/j.celrep.2025.115279 .

112. Y. Fan , G. Abrahamsen , J. J. McGrath , and A. Mackay-Sim , " Altered Cell Cycle Dynamics in Schizophrenia ," *Biological Psychiatry 71* , no. 2 (2012): 129 – 135 , https://doi.org/10.1016/j.biopsych.2011.10.004 .

113. G. F. Rushworth and I. L. Megson , " Existing and Potential Therapeutic Uses for N-acetylcysteine: The Need for Conversion to Intracellular Glutathione for Antioxidant Benefits ," *Pharmacology & Therapeutics 141* , no. 2 (2014): 150 – 159 , https://doi.org/10.1016/j.pharmthera.2013.09.006 .

114. T. G. Van Erp , P. A. Saleh , I. M. Rosso , et al., " Contributions of Genetic Risk and Fetal Hypoxia to Hippocampal Volume in Patients With Schizophrenia or Schizoaffective Disorder, Their Unaffected Siblings, and Healthy Unrelated Volunteers ," *American Journal of Psychiatry 159* , no. 9 (2002): 1514 – 1520 , https://doi.org/10.1176/appi.ajp.159.9.1514 .

115. I. M. Rosso , T. D. Cannon , T. Huttunen , M. O. Huttunen , J. Lönnqvist , and T. L. Gasperoni , " Obstetric Risk Factors for Early-onset Schizophrenia in a Finnish Birth Cohort ," *American Journal of Psychiatry 157* , no. 5 (2000): 801 – 807 , https://doi.org/10.1176/appi.ajp.157.5.801 .

116. M. Berk , G. S. Malhi , L. J. Gray , and O. M. Dean , " The Promise of N-acetylcysteine in Neuropsychiatry ," *Trends in Pharmacologial Sciences 34* , no. 3 (2013): 167 – 177 , https://doi.org/10.1016/j.tips.2013.01.001 .

117. C. C. Bell , " DSM-IV: Diagnostic and Statistical Manual of Mental Disorders ," *JAMA: The Journal of the American Medical Association 272* , no. 10 (1994): 828 – 829 , https://doi.org/10.1001/jama.1994.03520100096046 .

118. M. B. First , *Structured Clinical Interview for DSM-IV Axis I Disorders SCID-I* (American Psychiatric Press , 1997), 84 .

119. J. Yu , K. F. Chau , M. A. Vodyanik , J. Jiang , and Y. Jiang , " Efficient Feeder-Free Episomal Reprogramming With Small Molecules ," *PLoS One 6* , no. 3 (2011): 17557 , https://doi.org/10.1371/journal.pone.0017557 .

120. S. M. Kim , J. W. Kim , T. H. Kwak , et al., " Generation of Integration-free Induced Neural Stem Cells From Mouse Fibroblasts ," *Journal of Biological Chemistry 291* , no. 27 (2016): 14199 – 14212 , https://doi.org/10.1074/jbc.M115.713578 .

121. H. Li and R. Durbin , " Fast and Accurate Short Read Alignment With Burrows–Wheeler transform ," *Bioinformatics 25* , no. 14 (2009): 1754 – 1760 , https://doi.org/10.1093/bioinformatics/btp324 .

122. H. Li , B. Handsaker , A. Wysoker , et al., " The Sequence Alignment/Map Format and SAMtools ," *Bioinformatics 25* , no. 16 (2009): 2078 –

2079 , https://doi.org/10.1093/bioinformatics/btp352 .

123. A. McKenna , M. Hanna , E. Banks , et al., " The Genome Analysis Toolkit: A MapReduce Framework for Analyzing Next-Generation DNA Sequencing Data ," *Genome Res 20* , no. 9 (2010): 1297 – 1303 , https://doi.org/10.1101/gr.107524.110 .

124. A. Manichaikul , J. C. Mychaleckyj , S. S. Rich , K. Daly , M. Sale , and W. M. Chen , " Robust Relationship Inference in Genome-Wide Association Studies ," *Bioinformatics 26* , no. 22 (2010): 2867 – 2873 , https://doi.org/10.1093/bioinformatics/btq559 .

125. E. Talevich , A. H. Shain , T. Botton , and B. B. C. CNVkit , " CNVkit: Genome-Wide Copy Number Detection and Visualization From Targeted DNA Sequencing ," *PLOS Computational Biology 12* , no. 4 (2016): 1004873 , https://doi.org/10.1371/journal.pcbi.1004873 .

126. R. Yan , X. Cheng , and F. Guo , " Protocol for scChaRM-seq: Simultaneous Profiling of Gene Expression, DNA Methylation, and Chromatin Accessibility in Single Cells ," *STAR Protocols 2* , no. 4 (2021): 100972 , https://doi.org/10.1016/j.xpro.2021.100972 .

127. M. Martin , " Cutadapt Removes Adapter Sequences From High-throughput Sequencing Reads ," *EMBnetjournal 17* , no. 1 (2011): 10 – 12 , https://doi.org/10.14806/ej.17.1.200 .

128. A. Dobin , C. A. Davis , F. Schlesinger , et al., " STAR: Ultrafast Universal RNA-seq Aligner ," *Bioinformatics 29* , no. 1 (2013): 15 – 21 , https://doi.org/10.1093/bioinformatics/bts635 .

129. S. Anders , P. T. Pyl , and W. Huber , " HTSeq—a Python Framework to Work With High-Throughput Sequencing Data ," *Bioinformatics 31* , no. 2 (2015): 166 – 169 , https://doi.org/10.1093/bioinformatics/btu638 .

130. D. J. McCarthy , K. R. Campbell , A. T. Lun , and Q. F. Wills , " Scater: Pre-processing, Quality Control, Normalization and Visualization of Single-cell RNA-seq Data in R ," *Bioinformatics 33* , no. 8 (2017): 1179 – 1186 , https://doi.org/10.1093/bioinformatics/btw777 .

131. G. X. Y. Zheng , J. M. Terry , P. Belgrader , et al., " Massively Parallel Digital Transcriptional Profiling of Single Cells ," *Nature Communications 8* , no. 1 (2017): 14049 , https://doi.org/10.1038/ncomms14049 .

132. T. Stuart , A. Butler , P. Hoffman , et al., " Comprehensive Integration of Single-Cell Data ," *Cell 177* , no. 7 (2019): 1888 – 1902 , https://doi.org/10.1016/j.cell.2019.05.031 .

133. L. Haghverdi , A. T. L. Lun , M. D. Morgan , and J. C. Marioni , " Batch Effects in Single-cell RNA-sequencing Data Are Corrected by Matching Mutual Nearest Neighbors ," *Nature Biotechnology 36* , no. 5 (2018): 421 – 427 , https://doi.org/10.1038/nbt.4091 .

134. G. Yu , L.-G. Wang , Y. Han , and Q.-Y. He , " clusterProfiler: An R Package for Comparing Biological Themes Among Gene Clusters ," *Omics: a Journal of Integrative Biology 16* , no. 5 (2012): 284 – 287 , https://doi.org/10.1089/omi.2011.0118 .

135. J. van de Leemput , C. Boles Nathan , and R. Kiehl Thomas , " CORTECON: A Temporal Transcriptome Analysis of In Vitro Human Cerebral Cortex Development from Human Embryonic Stem Cells ," *Neuron 83* , no. 1 (2014): 51 – 68 , https://doi.org/10.1016/j.neuron.2014.05.013 .

136. V. K. Mootha , C. M. Lindgren , K. F. Eriksson , et al., " PGC-1alpha-responsive Genes Involved in Oxidative Phosphorylation Are Coordinately Downregulated in Human Diabetes ," *Nat Genet 34* , no. 3 (2003): 267 – 273 , https://doi.org/10.1038/ng1180 .

137. A. Subramanian , P. Tamayo , V. K. Mootha , et al., " Gene Set Enrichment Analysis: A Knowledge-based Approach for Interpreting Genome-Wide Expression Profiles ," *Proceedings of the National Academy of Sciences U S A 102* , no. 43 (2005): 15545 – 15550 , https://doi.org/10.1073/pnas.0506580102 .

138. D. M. Howard , M. J. Adams , T. K. Clarke , et al., " Genome-Wide Meta-Analysis of Depression Identifies 102 Independent Variants and Highlights the Importance of the Prefrontal Brain Regions ," *Nature Neuroscience 22* , no. 3 (2019): 343 – 352 , https://doi.org/10.1038/s41593-018-0326-7 .

139. E. A. Stahl , G. Breen , A. J. Forstner , et al., " Genome-Wide Association Study Identifies 30 Loci Associated With Bipolar Disorder ," *Nature Genetics 51* , no. 5 (2019): 793 – 803 , https://doi.org/10.1038/s41588-019-0397-8 .

140. C. A. de Leeuw , J. M. Mooij , T. Heskes , and D. Posthuma , " MAGMA: Generalized Gene-set Analysis of GWAS Data ," *PLoS Computational Biology 11* , no. 4 (2015): 1004219 , https://doi.org/10.1371/journal.pcbi.1004219 .

141. C. Trapnell , D. Cacchiarelli , J. Grimsby , et al., " The Dynamics and Regulators of Cell Fate Decisions Are Revealed by Pseudotemporal Ordering of Single Cells ," *Nature Biotechnology 32* , no. 4 (2014): 381 – 386 , https://doi.org/10.1038/nbt.2859 .

142. X. Qiu , A. Hill , J. Packer , D. Lin , Y. A. Ma , and C. Trapnell , " Single-cell mRNA Quantification and Differential Analysis With Census ," *Nature Methods 14* , no. 3 (2017): 309 – 315 , https://doi.org/10.1038/nmeth.4150 .

143. S. Kanton , M. J. Boyle , Z. He , et al., " Organoid Single-Cell Genomic Atlas Uncovers Human-Specific Features of brain development ," *Nature 574* , no. 7778 (2019): 418 – 422 , https://doi.org/10.1038/s41586-019-1654-9 .

144. D. M. Halliday , J. R. Rosenberg , P. Breeze , and B. A. Conway , " Neural Spike Train Synchronization Indices: Definitions, Interpretations, and Applications ," *IEEE Transactions on Biomedical Engineering 53* , no. 6 (2006): 1056 – 1066 , https://doi.org/10.1109/tbme.2006.873392 .

-----------------------End of Document-----------------------
