# Supplementary material for: Gestational Hypoxia Disrupts Medial Ganglionic Eminence Progenitor Dynamics and Interneuron Development in Schizophrenia
Source: Adv Sci (Weinh). 2026 Aug 12:e76936. Online ahead of print. doi: 10.1002/advs.76936 (PMC13464456; doi:10.1002/advs.76936)
Supplement: Supplementary file 3 [file ADVS-9999-e76936-s004.zip › Supplementary Table/Table S2. Short tandem repeat of all PBMC and iPSC lines.docx]

| **STR** | **HC1** | | **HC2** | | **HC3** | | **HC4** | | **HC5** | | **HC6** | | **FES1** | | **FES2** | | **FES3** | | **FES4** | |
| --- | --- | --- | --- | --- | --- | --- | --- | --- | --- | --- | --- | --- | --- | --- | --- | --- | --- | --- | --- | --- |
|  | **PBMCs** | **iPSCs** | **PBMCs** | **iPSCs** | **PBMCs** | **iPSCs** | **PBMCs** | **iPSCs** | **PBMCs** | **iPSCs** | **PBMCs** | **iPSCs** | **PBMCs** | **iPSCs** | **PBMCs** | **iPSCs** | **PBMCs** | **iPSCs** | **PBMCs** | **iPSCs** |
| **AMEL** | X | X | X | X | X/Y | X/Y | X/Y | X/Y | X/Y | X/Y | X | X | X | X | X | X | X | X | X/Y | X/Y |
| **Yindel** | - | - | - | - | - | - | 1 | 1 | 1 | 1 | - | - | - | - | - | - | - | - | 2 | 2 |
| **D3S1358** | 15/17 | 15/17 | 15/17 | 15/17 | 15/16 | 15/16 | 16 | 16 | 14/15 | 14/15 | 16/17 | 16/17 | 15 | 15 | 16 | 16 | 15/17 | 15/17 | 15/16 | 15/16 |
| **D13S317** | 10/12 | 10/12 | 8/11 | 8/11 | 10/12 | 10/12 | 8/10 | 8/10 | 8/10 | 8/10 | 9/11 | 9/11 | 8/10 | 8/10 | 10/11 | 10/11 | 8/9 | 8/9 | 9/11 | 9/11 |
| **D7S820** | 9 | 9 | 11/12 | 11/12 | 10/12 | 10/12 | 10/12 | 10/12 | 12 | 12 | 11 | 11 | 11 | 11 | 11/12 | 11/12 | 10 | 10 | 11/12 | 11/12 |
| **D16S539** | 9/12 | 9/12 | 9/12 | 9/12 | 9/11 | 9/11 | 10/12 | 10/12 | 9/12 | 9/12 | 9 | 9 | 10/12 | 10/12 | 12/14 | 12/14 | 10/11 | 10/11 | 11/12 | 11/12 |
| **D8S1179** | 10/14 | 10/14 | 15/16 | 15/16 | - | - | 10/15 | 10/15 | 12/14 | 12/14 | 12/15 | 12/15 | - | - | 10/11 | 10/11 | 10/14 | 10/14 | 15/16 | 15/16 |
| **Penta D** | 10/13 | 10/13 | 9/12 | 9/12 | 8/9 | 8/9 | 8/9 | 8/9 | 10/11 | 10/11 | 9 | 9 | 11/12 | 11/12 | 10/14 | 10/14 | 9/12 | 9/12 | 9/12 | 9/12 |
| **D19S433** | 13/14 | 13/14 | 14/15 | 14/15 | 14 | 14 | 13/15 | 13/15 | 13/15.2 | 13/15.2 | 13/14 | 13/14 | 15/15.2 | 15/15.2 | 13/13.2 | 13/13.2 | 14/15.2 | 14/15.2 | 13/14.2 | 13/14.2 |
| **D5S818** | 12/13 | 12/13 | 9/11 | 9/11 | 10/12 | 10/12 | 12 | 12 | 7/12 | 7/12 | 11 | 11 | 10/13 | 10/13 | 12/13 | 12/13 | 11/13 | 11/13 | 11/13 | 11/13 |
| **D21S11** | 31/32.2 | 31/32.2 | 30 | 30 | 30/31 | 30/31 | 29/32.2 | 29/32.2 | 30 | 30 | 29/30 | 29/30 | 29/32 | 29/32 | 29/30 | 29/30 | 29/31 | 29/31 | 29/31 | 29/31 |
| **TPOX** | 8/9 | 8/9 | 9/11 | 9/11 | - | - | 8 | 8 | 9 | 9 | 9 | 9 | - | - | 8 | 8 | 8/11 | 8/11 | 9/11 | 9/11 |
| **D1S1656** | 12/16 | 12/16 | 15/16 | 15/16 | - | - | 14/16 | 14/16 | 15 | 15 | 15/16 | 15/16 | - | - | 13/17 | 13/17 | 15/17 | 15/17 | 16/17 | 16/17 |
| **D6S1043** | 18/19 | 18/19 | 10/18 | 10/18 | 13 | 13 | 14/17.3 | 14/17.3 | 14/21 | 14/21 | 11/14 | 11/14 | 10/12 | 10/12 | 11/12 | 11/12 | 12/18 | 12/18 | 18 | 18 |
| **D2S441** | 12 | 12 | 10 | 10 | 11 | 11 | 11/12 | 11/12 | 11 | 11 | 10/15 | 10/15 | 9.1/10 | 9.1/10 | 10/11 | 10/11 | 14 | 14 | 12/14 | 12/14 |
| **D12S391** | 20/21 | 20/21 | 18/23 | 18/23 | 18/20 | 18/20 | 17/19 | 17/19 | 15/19 | 15/19 | 21 | 21 | 18/19 | 18/19 | 17/22 | 17/22 | 19 | 19 | 22/23 | 22/23 |
| **D2S1338** | 17 | 17 | 19 | 19 | 17/24 | 17/24 | 19/23 | 19/23 | 18/23 | 18/23 | 19/23 | 19/23 | 22/25 | 22/25 | 22/25 | 22/25 | 19/23 | 19/23 | 22/23 | 22/23 |
| **vWA** | 14/18 | 14/18 | 14/17 | 14/17 | - | - | 14/16 | 14/16 | 15/18 | 15/18 | 14/17 | 14/17 | - | - | 14/17 | 14/17 | 17 | 17 | 14/17 | 14/17 |
| **Penta E** | 17/21 | 17/21 | 5/20 | 5/20 | 13/14 | 13/14 | 12/24 | 12/24 | 9/16 | 9/16 | 17/18 | 17/18 | 17/19 | 17/19 | 12 | 12 | 13/18 | 13/18 | 11/16 | 11/16 |
| **TH01** | 6/9 | 6/9 | 7/9 | 7/9 | - | - | 7/9 | 7/9 | 9 | 9 | 9/9.3 | 9/9.3 | - | - | 9 | 9 | 7/9 | 7/9 | 7/9 | 7/9 |
| **D18S51** | 15 | 15 | 17/18 | 17/18 | - | - | 15/17 | 15/17 | 11/20 | 11/20 | 13 | 13 | - | - | 15/20 | 15/20 | 13 | 13 | 18/24 | 18/24 |
| **CSF1PO** | 10 | 10 | 10/11 | 10/11 | - | - | 11 | 11 | 10/11 | 10/11 | 11/12 | 11/12 | - | - | 10/12 | 10/12 | 10/12 | 10/12 | 10/11 | 10/11 |
| **FGA** | 21/22 | 21/22 | 23 | 23 | - | - | 24/24.2 | 24/24.2 | 23/24 | 23/24 | 24/27 | 24/27 | - | - | 21/26 | 21/26 | 19/23 | 19/23 | 23/24 | 23/24 |
| **D18S1364** | - | - | - | - | 16 | 16 | - | - | - | - | - | - | 14/16 | 14/16 | - | - | - | - | - | - |
| **D22S1045** | - | - | - | - | 16/17 | 16/17 | - | - | - | - | - | - | 16/17 | 16/17 | - | - | - | - | - | - |
| **Y-GATA-HA** | - | - | - | - | 10 | 10 | - | - | - | - | - | - | - | - | - | - | - | - | - | - |
| **D11S2368** | - | - | - | - | 20/21 | 20/21 | - | - | - | - | - | - | 17/20 | 17/20 | - | - | - | - | - | - |
| **D13S325** | - | - | - | - | 18/19 | 18/19 | - | - | - | - | - | - | 19/20 | 19/20 | - | - | - | - | - | - |

**Table S2. Short tandem repeat (STR) of all iPSC lines.**

**(Related to Results section).**

(PBMCs: Peripheral blood mononuclear cell; iPSCs: Induced pluripotent stem cells; HC: Healthy controls; FES: First episode schizophrenia.)
