# Supplementary material for: Gestational Hypoxia Disrupts Medial Ganglionic Eminence Progenitor Dynamics and Interneuron Development in Schizophrenia
Source: Adv Sci (Weinh). 2026 Aug 12:e76936. Online ahead of print. doi: 10.1002/advs.76936 (PMC13464456; doi:10.1002/advs.76936)
Supplement: Supplementary file 3 [file ADVS-9999-e76936-s004.zip › Supplementary Table/Table S3. Pathogen Detection.docx]

| **Pathogen detection** | **HC1** | **HC2** | **HC3** | **HC4** | **HC5** | **HC6** | **FES1** | **FES2** | **FES3** | **FES4** |
| --- | --- | --- | --- | --- | --- | --- | --- | --- | --- | --- |
| **HIV antibody** | - | - | - | - | - | - | - | - | - | - |
| **HBV**  **HBsAg**  **HBsAb**  **HBeAg**  **HBeAb**  **HBcAb** | -  +  -  -  - | -  +  -  -  - | -  -  -  -  - | -  -  -  -  - | -  +  -  -  - | -  +  -  -  - | -  +  -  -  - | -  -  -  -  - | -  -  -  -  - | -  -  -  -  - |
| **TPPA** | - | - | - | - | - | - | - | - | - | - |
| **HCV antibodies**  **HCV-IgM**  **HCV-IgG** | -  - | -  - | -  - | -  - | -  - | -  - | -  - | -  - | -  - | -  - |

**Table S3. Pathogen Detection.**

**(Related to Results section).**

(HIV: Human immunodeficiency virus; HBV: Hepatitis B virus; TPPA: Treponema pallidum-specific antibody; HCV: Hepatitis C virus.)
