# Supplementary material for: Gestational Hypoxia Disrupts Medial Ganglionic Eminence Progenitor Dynamics and Interneuron Development in Schizophrenia
Source: Adv Sci (Weinh). 2026 Aug 12:e76936. Online ahead of print. doi: 10.1002/advs.76936 (PMC13464456; doi:10.1002/advs.76936)
Supplement: Supplementary file 3 [file ADVS-9999-e76936-s004.zip › Supplementary Table/Table S6. Detailed sample information for scRNA-seq.docx]

| **Cell numbers** | | **0W** | | **3W** | | **8W** | | **16W** | |
| --- | --- | --- | --- | --- | --- | --- | --- | --- | --- |
|  |  | **Origin** | **After QC** | **Origin** | **After QC** | **Origin** | **After QC** | **Origin** | **After QC** |
| **HC** | **HC1** | 96 | 57 | 288 | 211 | 6518 | 5651 | 0 | 0 |
|  | **HC2** | 96 | 55 | 480 | 391 | 5282 | 5114 | 3086 | 2203 |
|  | **HC3** | 4762 | 3505 | 5872 | 5655 | 6681 | 5255 | 3695 | 1633 |
|  | **HC4** | 96 | 70 | 192 | 117 | 8348 | 6849 | 1785 | 1022 |
|  | **HC5** | 96 | 72 | 288 | 220 | 4790 | 4506 | 2920 | 1945 |
|  | **HC6** | 96 | 65 | 288 | 232 | 9796 | 7742 | 3469 | 2194 |
| **Total** | | **5242** | **3824** | **7408** | **6826** | **41415** | **35117** | **14955** | **8997** |
| **FES** | **FES1** | 96 | 75 | 288 | 192 | 5444 | 5169 | 4538 | 3623 |
|  | **FES2** | 96 | 48 | 96 | 60 | 6783 | 5355 | 0 | 0 |
|  | **FES3** | 96/10000 | 62/9470 | 480 | 379 | 5740 | 5326 | 1918 | 1105 |
|  | **FES4** | 96 | 71 | 288 | 80 | 7085 | 4827 | 4419 | 3690 |
| **Total** | | **10384** | **9726** | **1152** | **711** | **25052** | **20677** | **10875** | **8418** |

**Table S6. Detailed sample information for scRNA-seq.**

**(Related to Figure 2A).**

(Cells were processed using SCRB-seq (normal) and/or 10x Genomics platform (underline). Quality control (QC) criteria for 10x data: 1,000 < nCount_RNA < 25,000, 1,000 < nFeature_RNA < 6,000, and percent mitochondrial reads (percent.mt) < 10%. QC criteria for SCRB-seq data: 1,000 < nFeature_RNA < 7,000, 10,000 < nCount_RNA < 300,000, percent.mt < 15%, and mapping rate > 70%).
